# Treatment of acute pharyngitis in children: an Italian intersociety consensus (SIPPS-SIP-SITIP-FIMP-SIAIP-SIMRI-FIMMG)

**DOI:** 10.1186/s13052-024-01789-5

**Published:** 2024-11-06

**Authors:** Elena Chiappini, Giovanni Simeone, Marcello Bergamini, Roberta Pellegrino, Alfredo Guarino, Annamaria Staiano, Susanna Esposito, Guido Castelli Gattinara, Andrea Lo Vecchio, Stefania Stefani, Iride Dello Iacono, Immacolata Scotese, Giovanna Tezza, Giulio Dinardo, Simona Riccio, Sofia Pellizzari, Sonia Iavarone, Giulia Lorenzetti, Elisabetta Venturini, Daniele Donà, Luca Pierantoni, Mattia Doria, Silvia Garazzino, Fabio Midulla, Claudio Cricelli, Luigi Terracciano, Annalisa Capuano, Eugenia Bruzzese, Daniele Ghiglioni, Lara Fusani, Eleonora Fusco, Paolo Biasci, Lamberto Reggiani, Luigi Matera, Enrica Mancino, Elisa Barbieri, Antonio D’Avino, Laura Cursi, Maria Giuseppa Sullo, Silvestro Scotti, Gian Luigi Marseglia, Giuseppe Di Mauro, Nicola Principi, Luisa Galli, Maria Carmen Verga

**Affiliations:** 1 Department of Health Sciences, University of Florence, Florence, Italy; 2 Infectious Diseases Unit, Meyer Children’s Hospital IRCCS, Florence, Italy; 3 Primary Care Pediatrician, Mesagne, Brindisi, Italy; 4 Department of Translational Medical Sciences, University of Naples Federico II, Naples, Italy; 5 Paediatric Clinic, Department of Medicine and Surgery, University Hospital, University of Parma, Parma, Italy; 6 University Hospital Paediatric Department, Bambino Gesù Children’s Hospital, IRCCS, Rome, Italy; 7 Department of Biomedical and Biotechnological Sciences (BIOMETEC), Section of Microbiology, University of Catania, Catania, Italy; 8 Pediatric Unit, Fatebenefratelli Hospital, Benevento, Italy; 9 Department of Pediatrics, Ospedale San Maurizio, Bolzano, Italy; 10 Department of Woman, Child and of General and Specialized Surgery, University of Campania ’Luigi Vanvitelli’, Naples, Italy; 11 Department of Surgical Sciences, Dentistry, Gynecology and Pediatrics, Pediatric Clinic, University of Verona, Verona, Italy; 12 Onco-Hematology, Cell and Gene Therapy and Bone Marrow Transplant Clinic Area, Bambino Gesù Children’s Hospital, IRCCS, Rome, Italy; 13 Department of Pediatrics, University of Rome Tor Vergata, Bambino Gesù Children’s Hospital, IRCCS, Rome, Italy; 14 Division of Paediatric Infectious Diseases, Department of Women’s and Children’s Health, University of Padua, Padua, Italy; 15 Pediatric Emergency Unit, IRCCS Azienda Ospedaliero Universitaria Di Bologna, Bologna, Italy; 16 Department of Paediatrics, Infectious Diseases Unit, Regina Margherita Children’s Hospital, University of Turin, Turin, Italy; 17 Department of Maternal Child and Urological Sciences, Sapienza University of Rome, Rome, Italy; 18 Italian College of General Practitioners and Primary Care, (SIMG), Florence, Italy; 19 Pediatric Primary Care, National Pediatric Health Care System, Caserta, Italy; 20 Department of Experimental Medicine, University of Campania Luigi Vanvitelli, Naples, Italy; 21 Regional Center of Pharmacovigilance and Pharmacoepidemiology of Campania Region, Naples, Italy; 22 Pediatric Primary Care, Fondazione IRCCS Ca’ Granda Ospedale Maggiore Policlinico di Milano, National Pediatric Health Care System, 26, Rome, Italy; 23 FIMP (Federazione Italiana Medici Pediatri), Rome, Italy; 24 Primary Care Pediatricians, Azienda Unità Sanitaria Locale (AUSL), Imola, Italy; 25 Department of Pediatrics, Foundation IRCCS Policlinico San Matteo, University of Pavia, Pavia, Italy; 26 Paediatric Department I, University of Milan, Milan, Italy; 27 Primary Care Pediatrician, Vietri sul Mare, Salerno, Italy; 28 Department of Sciences for Health Sciences, Anna Meyer Children’s University Hospital, IRCCS, University of Florence, Viale Pieraccini, 24, Florence, 50100 Italy

**Keywords:** Pharyngitis, Streptococcus pyogenes, Tonsillitis, Group A Streptococcus

## Abstract

Sore throat represents one of the main causes of antibiotic overprescription in children. Its management is still a matter of debate, with countries considering streptococcal pharyngotonsillitis a benign and self-limiting condition and others advocating for its antibiotic treatment to prevent suppurative complications and acute rheumatic fever. Italian paediatricians frequently prescribe antibiotics on a clinical basis regardless of microbiological results. Moreover, broad-spectrum antibiotics are inappropriately prescribed for this condition. In this regard, an intersociety consensus conference was issued to promote the judicious use of antibiotic therapy in paediatric outpatient settings. A systematic review of the literature was performed, and updated recommendations were developed according to the GRADE methodology. Antibiotic treatment with amoxicillin (50 mg/kg/day) for 10 days is recommended in all children with proven streptococcal pharyngitis. Benzathine-penicillin could be prescribed in children with impaired intestinal absorption or inability to tolerate enteral intake and in those at high risk of suppurative complications with low compliance to oral therapy. In children with suspected amoxicillin allergy, third-generation cefalosporins for five days are recommended in low-risk patients, and macrolides are recommended in high-risk ones. Candidates for tonsillectomy due to recurrent pharyngitis could be treated with amoxicillin-clavulanic acid, clindamycin, or combined therapy with amoxicillin plus rifampicin for four days, in an attempt to avoid surgery.

**Supplementary Information:**

The online version contains supplementary material available at 10.1186/s13052-024-01789-5.

## Introduction

The management of acute pharyngotonsillitis in children is still a matter of debate, and current national and international guidelines recommend different diagnostic and treatment strategies [1–12]. Viral infections, including Adenovirus, Epstein-Barr virus, and Coxsackievirus, are the most common causes of acute pharyngitis [13]. *Streptococcus pyogenes* (Group A β-haemolytic streptococcus [GABHS]) is found in 1 out of 4 children with acute pharyngitis, although 10–21% of the children with microbiological GABHS evidence are carriers [14, 15]. Consequently, making an accurate diagnosis of streptococcal pharyngitis can be challenging due to the overlapping in the clinical picture between viral and bacterial illnesses [13]. This diagnostic conundrum leads to antibiotic overprescriptions. In particular, in Italy, acute pharyngitis is the second most common cause of antibiotic prescriptions among primary care paediatricians after upper respiratory tract infections [16, 17]. Streptococcal pharyngitis can lead to suppurative (i.e., peritonsillar/parapharyngeal abscess, otitis, sinusitis) or non-suppurative complications (i.e., acute rheumatic fever [ARF] and acute post-streptococcal glomerulonephritis [APSGN]). Mainly, two approaches can be identified worldwide: one advocating for the diagnosis and treatment of GABHS pharyngitis to prevent ARF; [1–8] the other supporting an unfavourable cost-benefit ratio of antibiotic treatment due to the low incidence of ARF in high-income countries and the benign and self-limiting nature of GABHS pharyngitis [9–12]. The guidelines from the Italian National Institute of Health Guidelines recommend that only children with clinical and microbiological evidence of GABHS pharyngitis should receive antibiotics [3]. However, in almost half of the cases, Italian paediatricians prescribe antibiotics in children solely on a clinical basis, disregarding microbiological results [18, 19]. Furthermore, nationwide surveys showed that broad-spectrum antibiotics are inappropriately prescribed in this context. Amoxicillin-clavulanic acid was prescribed in about 25% of Italian emergency units to treat acute pharyngitis in children, and third-generation cephalosporins were prescribed in about 28% of the cases in pediatric primary care [18, 19]. 

To promote appropriate antibiotic prescription and use in paediatric outpatient settings, an intersociety consensus document was developed providing updated recommendations for the treatment of acute pharyngitis in children and adolescents.

## Methods

A systematic review of the literature was conducted to issue recommendations regarding acute pharyngotonsillitis treatment. The review was performed according to the GRADE methodology and the PRISMA checklist (Preferred Reporting Items for Systematic Reviews and Meta-Analyses) as reported in the Additional file 1 [20, 21]. The following databases Embase, Scopus, PubMed, and Cochrane were systematically screened with a date restriction from 2012 to April 2024. The following terms were combined as reported in Additional file 2: “child”, “pharyngitis”, “tonsillitis”, “sore throat”, “streptococcus pyogenes”. Randomized controlled trials, observational studies, and systematic reviews with or without meta-analysis on antibiotic therapy in children older than one month with acute pharyngotonsillitis were included. Not pertinent studies were excluded. (Figures A2.1 and A2.2 in the additional file 2). The quality assessment of evidence is provided in Additional File 3 and 4. The following outcomes were considered:

1. Persistence of symptoms on the third day and at the end of therapy (important outcome).
2. Complications: acute rheumatic fever [ARF], acute post-streptococcal glomerulonephritis [APSGN] within two months, peritonsillar abscess within two months (critical outcome).
3. Risk of recurrence within 28–42 days (important outcome).

## Results

### Question 1. Should Group A β-haemolytic streptococcus (GABHS) pharyngotonsillitis be treated with antibiotics?

#### Summary of evidence

One systematic review (SR) aiming to assess the benefit of antibiotics for sore throat in paediatric and adult patients in primary care was included [22]. The SR was of moderate methodological quality and included randomised controlled trials (RCTs) or quasi-RCTs. Twenty-seven studies with 12,835 cases of sore throat were included in the SR: 11 evaluated the antibiotic effect in GABHS-positive patients [23–33], one study included both GABHS-positive and negative patients and reported results separately [34], and 2 studies excluded GABHS-positive patients [35, 36]. The meta-analyses by Spinks et al. showed that antibiotics were more effective in reducing symptoms (sore throat, fever and headache) on day 3 in patients with a positive GABHS pharyngeal swab (15 studies, 3621 patients; relative risk [RR] = 0.58; 95% confidence interval [CI] 0.48 to 0.71; number needed to treat [NNT] = 6). The efficacy was lower in GABHS-negative cases (6 RCTs, 736 patients; RR = 0.78; 95% CI 0.63 to 0.97; NNT = 21). Similarly, at week 1, the RR was 0.29 (95% CI 0.12 to 0.70) in patients with GABHS pharyngotonsillitis (7 RCTs, 1117 patients) [22]. Compared with patients receiving placebo, those treated with antibiotics showed a reduced risk of developing acute otitis media (AOM) within 14 days (RR = 0.30; 95% CI 0.15 to 0.58) or peritonsillar abscess within 2 months (RR = 0.15; 95% CI 0.05 to 0.47). On the contrary, no risk reduction was showed considering acute sinusitis within 14 days (RR = 0.48; 95% CI 0.08 to 2.76 – not statistically significant). Concerning non-suppurative complications, in 14 studies, including 8,175 patients, antibiotics showed to reduce the ARF risk by more than two-thirds within two months (RR = 0.27; 95% CI 0.14 to 0.50). Conversely, the preventive effect of antibiotics on the risk of APSGN was not significant, and results were limited by the small number of cases (low-quality RCTs, 2 cases out of 5,147 patients).

#### Conclusion

In GABHS pharyngotonsillitis, antibiotic therapy is associated with reduction in acute symptoms by day 3, with a RR of 0.58 (95% CI 0.48 to 0.71; NNT = 6), and by day 7, with a RR of 0.29 (95% CI, 0.12 to 0.70). This therapeutic approach also significantly reduces the likelihood of developing suppurative complications, such as AOM and peritonsillar abscess, and ARF.

#### Recommendation

1. Antibiotic therapy is recommended in children with GABHS pharyngotonsillitis for a faster resolution of symptoms and a reduction in the risk of suppurative and non-suppurative complications (Quality of evidence high. Strong recommendation in favour of intervention).

### Question 2. Should amoxicillin be considered the antibiotic of choice in the treatment of GABHS pharyngotonsillitis besides penicillin V?

#### Summary of evidence

Since penicillin V is not available in Italy, the panel decided to explore the available evidence on the use of amoxicillin in children with GABHS pharyngotonsillitis. Our analysis included 2 SRs [37, 38] and two RCTs [39, 40], comparing the efficacy and safety of several oral antibiotics, administered for 10 days or shorter, to penicillin V therapy for 10 days. The SRs were of high methodological quality, whereas the RCTs were of low to moderate quality [37–40]. 

The SR by van Driel et al. included studies conducted on adult and paediatric outpatients [38]. Whereas, the SR by Altamimi et al. and the RCT by Li et al. were limited to the paediatric population [37, 40]. Details of the characteristics of the studies and results are provided in Tables A5.2 and A5.3 in the Additional file 5.

The SR by Altamimi et al. evaluated 20 RCTs, including 13,102 cases of GABHS pharyngotonsillits. Of these, only 3 studies assessed the complication rates [37]. 

Azithromycin (AZT) administered at 20 mg/kg was associated with lower rates of early treatment failure compared to the standard regimen with penicillin V (OR: 0.08; 95% CI: 0.01 to 0.64). Conversely, no statistically significant differences were observed among AZT administered at 10 mg/kg, clarithromycin, cefuroxime, or other antibiotics.

Regarding the risk of recurrence, the comparative analysis of various molecules yielded no statistically significant differences (OR = 0.95 [95% CI:0.83 to 1.08]). Three of the included studies reported complications related to GABHS infection [39–41]. The meta-analysis reported no risk difference (RD) in the development of ARF and/or APSGN between patients treated with antibiotics different from penicillin V and controls (OR = 0.53 [95% CI:0.17 to 1.64]) [39–41]. 

The SR by Van Driel et al. included 5,839 participants with confirmed GABHS infection, aged from 1 month to 80 years [38]. Most of the included studies were conducted within outpatient settings. Six studies compared penicillin with cephalosporins, six with macrolides, three with carbacephem, one with sulphonamides, one compared clindamycin with ampicillin, and one azithromycin with amoxicillin in children. They evaluated the efficacy in: (a) alleviating symptoms (pain, fever); (b) shortening the duration of the illness; (c) reducing clinical relapses (i.e., the resurgence of symptoms post-initial resolution); and (d) avoiding complications (suppurative complications, ARF, and APSGN). Furthermore, the incidence of adverse effects and the risk-benefit ratio associated with the diverse antibiotic regimens were also assessed [38].

##### Cephalosporins versus penicillin

No significant differences were reported about symptom resolution (2 to 15 days) for cephalosporins compared to penicillin (OR for lack of symptom resolution 0.79, 95%CI = 0.55 to 1.12; 5 studies; 2018 participants; low-quality evidence). The clinical recurrence was not significantly lower for cephalosporins compared to penicillin (OR = 0.55, 95% CI 0.30 to 0.99; NNT = 50; 4 studies; 1,386 participants; low-quality evidence). In the intention-to-treat subgroup analysis (855 children), the risk of treatment failure did not differ significantly (OR = 0.83, 95% CI = 0.40 to 1.73). No difference in the adverse event rates among groups was reported [38].

##### Macrolides versus penicillin

The difference in symptom resolution between macrolides and penicillin was not statistically significant across groups (OR = 1.11, 95%CI = 0.92 to 1.35; 6 studies; 1,728 participants; low-quality evidence). Similarly, the risk of clinical recurrence did not differ significantly (OR = 1.21, 95% CI 0.48 to 3.03; 6 studies; 802 participants; low-quality evidence) [38].

##### Azithromycin versus amoxicillin

Symptom resolution did not show a significant difference when comparing a single dose of Azithromycin (AZT) with a 10-day course of amoxicillin (OR = 0.76, 95% CI 0.55 to 1.05; 1 study; 673 participants; very low-quality evidence). No significant difference was observed considering recurrence rate (OR = 0.88, 95% CI = 0.43 to 1.82; 1 study; 422 participants; very low quality of evidence). However, adverse events were more frequently associated with AZT than amoxicillin treatment (OR = 2.67, 95%CI = 1.78 to 3.99; 1 study; 673 participants; very low quality of evidence) [38].

Kuroki et al. assessed the efficacy in GABHS eradication comparing a 3-days course of amoxicillin-clavulanate with amoxicillin given for 10 days in a low-quality RCT including 119 children (2–13 years, mean age 5.6 years) with proven GABHS pharyngotonsillitis [39].

The eradication rate of GABHS at 1–2 weeks after the end of treatment was 65.4% in the amoxicillin/clavulanate group and 85.4% in the amoxicillin group (*p* < 0.05). However, there was no statistically significant difference in clinical resolution, and clinical relapse/recurrence was rarely observed even in patients with subsequent GABHS isolation [39].

Li et al. evaluated the clinical impact of AZT (10 mg/kg once daily for 3 days), cefaclor (20 mg/kg/ day in 3 doses for 5 days) and amoxicillin (30 mg/kg/day in 3 doses for 10 days) in 256 children with proven GABHS pharyngotonsillitis with good adherence to therapy and clinical and microbiological assessments at the end of treatment (day 14) and at follow-up (day 30) [40]. Clinical success rate was 96.4% in children treated with AZT, 92.4% in those receiving cefaclor, and 91.0% in the amoxicillin group. However, no statistically significant difference was found among the 3 groups (AZT vs. cefaclor vs. amoxicillin) considering bacteriological eradication rate at the end of therapy (94.0%, 89.9% and 88.5%), and relapse rate (2.6%, 7.0% and 5.9%) [40].

#### Conclusion

The considered antibiotic regimens showed similar efficacy, and no relevant differences in the adverse event rates. No conclusion could be drawn for long-term complications since they were rarely reported. AZT at a dosage of 10 mg/kg/day was associated with an increased risk of recurrence and failure to eradicate GABHS. In contrast, AZT administration at 20 mg/kg/day was associated with increased clinical and microbiological success rate compared to a 10-day course of penicillin V. However, this higher dosage AZT regimen was associated with an increased incidence of adverse events, without a significant impact on recurrence risk. Notably, all studies were conducted in high-income countries, characterised by a low prevalence of streptococcal complications. Consequently, the applicability of these findings to regions with a high incidence of ARF is limited. Collectively, the research endorses the selection of amoxicillin as the preferred antibiotic drug for GABHS pharyngotonsillitis, when penicillin V is not available.

#### Recommendations

2. Amoxicillin is recommended as first-choice antibiotic drug in children with GABHS pharyngotonsillitis. (Quality of evidence high. Strong recommendation in favour of intervention).

### Question 3. Should the duration of antibiotic therapy for GABHS pharyngotonsillitis be shorter than 10 days?

#### Summary of evidence

Most guidelines advocate for a 10-day treatment with penicillin V or amoxicillin for patients with GABHS pharyngotonsillitis; the exceptions are the 2018 NICE (National Institute for Health and Care Excellence) guidelines (5–10 days) and the 2021 German guidelines (5–7 days) [10, 12, 17]. A Cochrane systematic review did not find evidence of the efficacy of a 10-day antibiotic course in preventing suppurative and non-suppurative complications [22]. The primary outcome for most studies was the eradication rate of GABHS, while the difference in ARF incidence was not analyzed due to the low occurrence rate [22]. The SR by Altamimi et al. included a study comparing amoxicillin (25 mg/kg/dose twice a day) for 6 days to penicillin V for 10 days [37]. This study included 321 patients aged 3–15 years (mean age 5.9 years) of these 318 were evaluated in the safety analyses and 277 (86.3%) in the efficacy analyses. The patients were followed-up for one month after the end of therapy and the results showed that the efficacy and safety of 6-days course of amoxicillin (50 mg/kg/day twice a day) were not significantly different from those of 10-days course of penicillin V (45 mg/kg/day three times a day) for GABHS pharyngotonsillitis (efficacy odds ratio (OR) = 0.82; 95% CI 0.37 to 1.79) [37]. 

#### Conclusion

To date, only one study assessed a shorter duration of amoxicillin therapy compared to the standard regimen (penicillin V for 10 days), showing that it was equally effective for symptoms resolution. However, the latter results have not been reproduced, and no conclusion regarding long-term complications could be drawn due to a follow-up limited to 30 days. Therefore, the results do not provide sufficient evidence of efficacy and safety regarding critical outcomes such as the risk of suppurative and non-suppurative complications. Furthermore, the results cannot be generalised to settings with different ARF incidence rates. A therapy shorter than 10 days has been associated to a decreased risk of non-adherence to treatment.

Given the lack of robust evidence, the recommendation for a 10-day course of therapy is conservatively upheld, particularly in light of the increased risk of ARF in several Italian regions. Some authors suggested that the focus should be on minimising unnecessary prescriptions rather than on reducing the duration of antibiotic therapy [42]. 

#### Recommendation

3. Given the lack of robust evidence regarding the efficacy and safety of different therapy durations in reducing the risk of suppurative and non-suppurative complications, amoxicillin for 10 days is recommended in children with GABHS pharyngotonsillitis. The latter regimen is recommended also in regions with a low ARF incidence. (Quality of evidence very low. Weak recommendation in favour of intervention).

### Question 4. In children allergic to penicillin, which antibiotics can be administered for the treatment of GABHS pharyngotonsillitis?

#### Summary of evidence

The prevalence of proven penicillin allergy is low, ranging from 0.7 to 1%, and the anaphylaxis prevalence is about 0.015 − 0.004%. Nonetheless, allergic reactions to penicillin are frequently suspected and lead to inappropriate antibiotics prescriptions [41, 43, 44]. 

A maculopapular rash, urticaria, and vomiting are common signs of both allergic reactions and infections, particularly viral ones. Pediatricians often need to decide whether these symptoms in patients treated with penicillin or amoxicillin are due to an allergic reaction. This assessment is crucial for deciding whether an alternative antibiotic is necessary, often without the support of an allergological consultation. To avoid inappropriate therapies, it is important to consider specific diagnostic criteria as stated by the the European Academy of Allergy & Clinical Immunology (EAACI) Position Paper [45]. The choice of the alternative antibiotic should be guided by the risk stratification for a severe reaction (2nd-3rd generation cephalosporins in low-risk patients, macrolide in high-risk patients) and its efficacy for a given disease [38, 40]. Macrolide-resistant GABHS strains are globally reported, with rates varying by region, reaching up to 18% in Italy in recent years. Therefore, local epidemiological data on macrolide-resistant GABHS prevalence should be considered when choosing the optimal management of penicilllin-allergic children [46]. 

#### Conclusions

In patients with GABHS pharyngotonsillitis and suspected penicillin allergy, who have not undewent an allergological work-up, the alternative antibiotic regimen (2nd-3rd generation cephalosporin or macrolides) should be prescribed based on the stratification of the risk for severe reactions and on local macrolide-resistant GABHS strains prevalence.

#### Recommendations

- 4a. In patients with GABHS pharyngotonsillitis with suspected amoxicillin allergy who have not uderwent an allergological work-up, the choice of alternative antibiotic (3rd generation cephalosporins or macrolides) must be based on careful risk stratification (Quality of the evidence very low. Opinion of the experts. Strong recommendation in favour of intervention).
- 4b. In patients with GABHS pharyngotonsillitis with suspected amoxicillin allergy and a low risk of an allergic reaction, a 3rd generation cephalosporin for 5 days should be recommended as an alternative therapy, restricting the use of macrolides to patients at high risk of severe allergic reactions (Quality of evidence very low. Weak recommendation in favour of intervention).

### Question 5: Which antibiotic(s) should be recommended as first-choice therapy for relapsing GABHS pharyngotonsillitis despite several courses of amoxicillin?

The current medical consensus lacks a clear definition of relapsing pharyngotonsillitis. However, the 1981 “Paradise’s criteria” are often used and include having seven episodes in one year, five per year for two years, or three episodes per year for three years [47]. Considering that GABHS carriers can be misdiagnosed with relapsing GABHS pharyngotonsillitis due to other etiologies, an accurate diagnosis is crucial.

Tonsillectomy is the only treatment proven to significantly reduce the frequency of episodes. However, it may potentially result in postoperative complications, including hemorrhage, adverse effects from opioid analgesics, and an increased risk of infections [48]. As a result, antibiotics have been proposed as an alternative approach.

A Cochrane review by Ng et al. on antibiotic therapy in recurrent pharyngotonsillitis could not draw any conclusions as none of the RCTs met the inclusion criteria [49]. A SR by Munck et al. in 2018 compared the efficacy of several antibiotics to oral penicillin in reducing pharyngotonsillitis incidence [50]. Efficacy was assessed considering three different clinical scenarios in children and adults: patients with relapsing pharyngotonsillitis without ongoing infection (Q1), those with ongoing infection (Q2), and those with recurrence within four weeks post-antibiotic therapy (Q3) [50]. Three RCTs were included in the SR for Q1, two for Q2, and none for Q3. Lildholdt et al. found no significant difference in the reduction of relapses between azithromycin and a placebo [51]. Two studies by Brook et al. in 1989 demonstrated the superiority of clindamycin and amoxicillin/clavulanic acid over oral penicillin in symptom resolution and eradication of beta-lactamase-producing bacteria, suggesting that they may play a role in the relapse pathogenesis (RR = 0.15, CI 95% 0.04–0.56, *p* = 0.005 for clindamycin; RR = 0.19, CI 95% 0.05–0.75, *p* = 0.018 for amoxicillin/clavulanic acid) [52, 53]. However, these RCTs had a high risk of bias and significant heterogeneity in patients’ age, number of episodes per year, and antibiotic treatments. The SR concluded that penicillin might be inadequate for relapsing pharyngotonsillitis treatment. Whereas, amoxicillin/clavulanic acid or clindamycin, which are effective against beta-lactamase-producing bacteria, could prevent further relapses, although current evidence supporting their efficacy is limited [50]. Similarly, in 2024 a SR by Hung et al. assessed the efficacy of different antibiotics for the eradication of GABHS in asymptomatic carriers [54]. Three RCTs, published between 1985 and 1991, were included. Negativization of pharyngeal swab culture was used to evaluate the eradication in all these studies [55–57]. Brook et al. included both children and young adults (age range 8–24 years) [55], the remaining studies were restricted to the pediatric population [56, 57]. The combined antibiotic regimen based on a single dose of intramuscular penicillin G followed by 4 days of oral rifampicin was assessed in 2 studies, and the eradication ranged between 68% [95% CI: 49–88%] and 93% [95% CI: 79–100%] at 3 weeks post-treatment [56, 57]. Penicillin monotherapy, either intramuscular or oral, and oral erythromycin reported eradication rates comparable to no treatment: 30% (95% CI 1.6–58%), 14% (95% CI 0–33%), 43%(95% CI 17–69%), and 23% (95% CI 0.2–46%) [54]. On the contrary, the 10-day clindamycin regimen, evaluated in 2 studies, was the most efficacious strategy achieving eradication in 93% (95% CI 81–100%) of cases at 10 days and in 100% (95% CI 79–100%) at 3 weeks after treatment [54]. However, Hung et al. advised caution in the widespread use of clindamycin due to the increasing resistance of GABHS to macrolides and clindamycin [54]. 

#### Conclusion

Given the evidence, it’s not possible to recommend a specific antibiotic therapy for recurrent GABHS pharyngotonsillitis. If avoiding tonsillectomy is a priority, amoxicillin-clavulanate, clindamycin, or a combination therapy with amoxicillin plus rifampicin might be considered as alternatives. However, these recommendations are issued with the caveat that the evidence supporting their use is currently limited.

#### Recommendation

5. In the case of relapsing GABHS tonsillitis, antibiotic therapy should be recommended only in patients candidated to tonsillectomy, as an attempt to avoid surgery. In these cases, amoxicillin-clavulanic acid, clindamycin, or a combination of amoxicillin plus rifampicin in the last 4 days of treatment should be prescribed. (Quality of evidence low. Weak recommendation).

### Question 6: which is the appropriate dosage of Amoxicillin in the treatment of GABHS pharyngotonsillitis?

In the management of acute GABHS pharyngotonsillitis, amoxicillin dosage for children under 40 kg varies from 40 to 90 mg/kg/day, not exceeding 3 g/day, and is typically divided into two or three doses. A good-quality RCT, including 517 children aged 2–12 years with confirmed GABHS pharyngotonsillitis, compared the efficacy and safety of a standard amoxicillin regimen of 40 mg/kg/day in 3 doses with a twice-daily regimen (45 mg/kg/day divided in 2 doses) [58]. A positive clinical response in over 96% of patients at the end of treatment (Day 11) was registered in both groups, with successful bacteriological responses in more than 94% of patients, showing that a twice-daily regimen was as effective and as well tolerated as the standard one [58]. Nakao et al. showed that a single daily dose of amoxicillin, ranging from 40 to 50 mg/kg/day for ten days, was as effective as multiple daily doses in children with streptococcal pharyngotonsillitis older than three years [59]. 

The Summary of Product Characteristics (SPC) provided by the European and Italian Medicines Agencies recommend a dosage of 50 mg/kg/day divided in 2 doses for the treatment of streptococcal pharyngotonsillitis [60, 61]. 

#### Conclusion

Amoxicillin administered at 50 mg/kg/day in two doses is as effective and as well-tolerated as 40 mg/kg/day given three times a day for treating acute GABHS pharyngotonsillitis. It is thus reasonable to conclude that a twice-daily regimen may be associated to enhanced adherence to treatment.

#### Recommendation

6. In children with GABHS pharyngotonsillitis, a daily dose of amoxicillin (50 mg/kg/day) divided into 2 administrations may be recommended to improve treatment adherence. (Quality of evidence low. Weak recommendation in favour of intervention.)

### Question 7: May parenteral antibiotics, specifically intramuscular benzathine-penicillin, be recommended as treatment alternative to oral amoxicillin in selected GABHS pharyngotonsillitis patients?

Parenteral antibiotic therapy is usually considered in children with low adherence to oral treatment. A RCT conducted in Ghana, including 99 paediatric patients, compared the efficacy of amoxicillin and benzathine-penicillin in the treatment of GABHS pharyngitis [62]. Treatment failure rate was higher in those treated with amoxicillin than in the penicillin group (18.9% vs. 6.4%, respectively [*p* ≥ 0.05]). However, amoxicillin was administered as a single daily dose [62]. In a recent retrospective, multicenter study conducted in Israel, involving 242,366 pediatric patients enrolled over a decade (2010–2019), treatment with amoxicillin or penicillin V was associated with a significantly lower risk of streptococcal complications (OR = 0.68, 95% CI 0.52–0.89; *P* < 0.01) and reduced rates of medical re-evaluation compared to benzathine penicillin treatment [63]. Given the overlapping efficacy, oral treatment with amoxicillin should be preferred to parenteral antibiotics because it is less invasive and stressful for the child. Furthermore, benzathine-penicillin must be administered by trained healthcare workers, it can be painful and has an overall higher cost than oral amoxicillin.

#### Conclusion

Benzatin-penicillin antibiotic therapy should be limited to patients with reduced gastrointestinal absorption, inability to take oral therapies or poor adherence to oral amoxicillin.

#### Recommendations

- 7a. In children with GABHS pharyngotonsillitis, benzathine-penicillin therapy should only be recommended for subjects with reduced gastrointestinal absorption or inability to take oral therapies. (High quality of evidence. Weak recommendation in favour of intervention)
- 7b. In children with GABHS pharyngotonsillitis, benzathine-penicillin therapy may be recommended in case of poor adherence to oral amoxicillin, especially in patients at high risk of suppurative complications (such as in cases of primary or secondary immunodeficiency). (Very low quality of evidence, expert opinion. Weak recommendation in favour of intervention)

### Question 8. Is it recommended to treat non-streptococcal bacterial pharyngotonsillitis (Fusobacterium spp., other anaerobes, Staphylococcus aureus, etc.) with antibiotic therapy?

Pharyngotonsillitis often leads to antibiotic prescriptions, regardless of proven GABHS infection, especially if the patient is febrile. However, antibiotic therapy is highly controversial in non-streptococcal bacterial pharyngotonsillitis. In this regard, 2 high-quality SRs of RCTs with meta-analysis were retrieved [22, 64]. The SR by Spinks et al. included studies evaluating the benefits of antibiotics for sore throats in children and/or adult patients [22]. Of these, one study reported outcome differences between GABHS-positive and negative patients [34] and two studies specifically excluded GABHS-positive patients [35, 36]. In GABHS-negative patients, antibiotics were not effective in reducing symptoms by day 3 (RR = 0.78; 95% CI 0.63–0.97 – NNT = 21 [15 RCTs, high quality, 3,621 patients]) and day 7 (RR = 0.73; CI 95% 0.50 to 1.07. Not statistically significant [5 RCTs, high quality, 541 patients]) [22]. Concerning the risk of suppurative complications, only one study reported a significant reduction in the incidence of acute otitis media within 14 days (RR = 0.06 [CI 95% from 0.1 to 03]) [36]. The SR by Spurling et al. assessed the clinical benefits, bacterial resistance, and patient satisfaction with delayed/no use of antibiotics for upper respiratory tract infections (fever, sore throat, cough, etc.) in primary care patients and emergency departments [64]. Specifically, four studies evaluated ‘sore throat’ in children [28, 65–67]. Of these, only Little et al. included patients without GABHS infection [67]. The clinical outcomes were heterogeneous, and in most cases, no significant difference was observed between delayed antibiotic therapy, immediate antibiotic administration, or no antibiotic therapy [64]. Due to insufficient data, it was not possible to aggregate study data for the comparison between delayed antibiotic administration and no antibiotic therapy.

#### Conclusions

In patients without comorbidities, non-GABHS pharyngotonsillitis has limited clinical significance. The study findings showed that the disease is generally self-limiting and that antibiotic therapy does not significantly reduce the duration of symptoms or the risk of complications. Therefore, a microbiological test to identify the aetiological agent is not recommended.

#### Recommendations

- 8a. Antibiotic therapy is not recommended in patients without comorbidities with pharyngotonsillitis and a culture and/or molecular test positive for bacteria other than GABHS. (Quality of evidence moderate. Strong recommendation against intervention)
- 8b. If antibiotic therapy for pharyngotonsillitis with a culture and/or molecular test positive for bacteria other than GABHS is considered appropriate, infectious disease counseling is recommended. (Quality of evidence very low. Expert opinion. Strong recommendation in favour of intervention)

## Discussion

Acute pharyngitis is a common condition among children, with 288.6 million episodes of streptococcal pharyngitis occurring globally each year [68]. It has been estimated that in 2020 sore throat led to 8.6 million courses of antibiotics in children (5–14 years) [69]. In this context, broad-spectrum antibiotics are frequently prescribed in the Italian pediatric outpatient setting [18, 19]. This consensus document aims to guide healthcare providers in the judicious use of antibiotics for acute pharyngotonsillitis in children and adolescents. Antibiotic treatment with amoxicillin (50 mg/kg/day administered in 2 divided doses) for 10 days is recommended in all children with confirmed streptococcal pharyngitis. Intramuscular benzathine-penicillin could be considered in children with impaired intestinal absorption. Similarly, it represents a valid therapeutic option in those at high risk for suppurative complications, such as immunodeficient children, with low compliance to oral therapy. In children with a suspected amoxicillin allergy, the risk of severe allergic reaction should be evaluated according to the EAACI (European Academy of Allergy & Clinical Immunology) position paper [45]. Macrolides could be prescribed as an alternative therapy in high-risk patients and third-generation cefalosporins for five days in low-risk ones. Data regarding the treatment of recurrent streptococcal pharyngitis are lacking. However, in children candidates to tonsillectomy, antibiotics could be prescribed as an attempt to avoid surgery. In the latter case, amoxicillin-clavulanic acid, clindamycin, or a combined therapy with amoxicillin plus 4-days rifampicin should be used. The panel recommends against antibiotic treatment in previously healthy patients with acute pharyngotonsillitis and evidence of non-GABHS bacterial infection. However, antibiotic therapy could be considered in children with underlying conditions, in which case a consultation with an infectious disease specialist is warranted.

## Supplementary Information

Additional file 1: Structure and Methodology of the Document. A thourough description of the structure and methodology of the consensus and is provided is this additional file.

Additional file 2: Search strategy. A thourough description of the search strategy is provided in this addiotional file, including the PICO questions, Keywords and the search strings used for each database. The flow diagrams of literature search and data extraction for systematic reviews and clinical studies are provided as Figure A2.1 and A2.2.

Additional file 3: Quality assessment of systematic reviews. The quality of systematin reviews has been assessed through the AMSTAR 2 checklist. The results are provided in the Additional file 3.

Additional file 4: Quality of evidence. For each question the quality of evidence was assessed according to the GRADE methodology and results are provided as tables in the Additional file 4 (Tables A4.1- A4.7).

Additional file 5: Characteristics, Results and Conclusions of the included studies. Characteristics, Results and Conclusions of the included studies for each question are summarized in tables A3.1 – A3.8.

## Data Availability

All the data that support the findings of this study are available from the corresponding author upon reasonable request.

## Abbreviations

AOM: Acute otitis media
ARF: Acute rheumatic fever
APSGN: Acute post-streptococcal glomerulonephritis
AZT: Azithromycin
CI: Confidence interval
GABHS: Group A β-haemolytic streptococcus
GL: Guideline
NNT: Number needed to treat
OR: Odds ratio
SPC: Summary of Product Characteristics
SR: Systematic review
RCT: Randomized controlled trial
RD: Risk difference
RR: Relative risk

## References

[1] Shulman ST, Bisno AL, Clegg HW, Gerber MA, Kaplan EL, Lee G, et al. Clinical practice guideline for the diagnosis and management of group A streptococcal pharyngitis: 2012 update by the Infectious Diseases Society of America. Clin Infect Dis. 2012;55:e86-102. 10.1093/cid/cis629. 22965026 10.1093/cid/cis629 PMC7108032

[2] Gerber MA, Baltimore RS, Eaton CB, Gewitz M, Rowley AH, Shulman ST, et al. Prevention of rheumatic fever and diagnosis and treatment of acute streptococcal pharyngitis. Circulation Am Heart Association. 2009;119:1541–51. 10.1161/CIRCULATIONAHA.109.191959. 10.1161/CIRCULATIONAHA.109.191959 19246689

[3] Chiappini E, Principi N, Mansi N, Serra A, De Masi S, Camaioni A, et al. Management of acute pharyngitis in children: summary of the Italian National Institute of Health guidelines. Clin Ther United States. 2012;34:1442-1458.e2. 10.1016/j.clinthera.2012.04.028. 10.1016/j.clinthera.2012.04.028 22691611

[4] Canadian Paediatric Society. Group A streptococcal (GAS) pharyngitis: A practical guide to diagnosis and treatment | Canadian Paediatric Society. 2021 Accessed 2022; https://cps.ca/en/documents//position//group-a-streptococcal/.

[5] Cohen R, Azria R, Barry B, Bingen E, Cavallo JD, Chidiac C, et al. Antibiotherapie par voie generale en pratique courante dans les infections respiratoires hautes de l’adulte et l’enfant. 2011. Available from: https://www.infectiologie.com/UserFiles/File/medias/Recos/2011-infections-respir-hautes-recommandations.pdf. Accessed 29 Apr 2022.

[6] Pelucchi C, Grigoryan L, Galeone C, Esposito S, Huovinen P, Little P, et al. Guideline for the management of acute sore throat. Clin Microbiol Infect. 2012;18:1–27. 10.1111/j.1469-0691.2012.03766.x. 22432746 10.1111/j.1469-0691.2012.03766.x

[7] Piñeiro Pérez R, Álvez González F, Baquero-Artigao F, Cruz Cañete M, Fernández J, Landaluce A, et al. Actualización del documento de consenso sobre el diagnóstico y tratamiento de la faringoamigdalitis aguda. Anales D Pediatría. 2020;93:206.e1-206.e8. 10.1016/j.anpedi.2020.05.004. 10.1016/j.anpedi.2020.05.004 32605870

[8] Finnish Medical Society Duodecim, Finnish Association for Central Practice, Finnish Otolaryngological Society, Infectious Diseases Society of Finland, Clinical Microbiologists Society. Sore throat. Current Care Summary. 2020; https://www.kaypahoito.fi/en/ccs00095. Accessed 11 Jun 2022.

[9] Scottish Intercollegiate Guidelines Network. Management of sore throat and indications for tonsillectomy: a national clinical guideline. 2010. Available from: https://www.sign.ac.uk/media/1055/sign117.pdf. Accessed 29 Apr 2022.

[10] National Institute for Health and Care Excellence. Sore throat (acute): antimicrobial prescribing [NG84]. 2018. Available from: https://www.nice.org.uk/guidance/ng84. Accessed 26 Apr 2022.

[11] de Jongh E, Opstelten W, Werkgroep NHG-Standaard Acute keelpijn. [Revision of the Dutch College of General Practitioners practice guideline ’acute sore throat’]. Ned Tijdschr Geneeskd. 2015;159:A9456. 26332822

[12] Krüger K, Töpfner N, Berner R, Windfuhr J, Oltrogge JH. Sore throat. Deutsches Ärzteblatt Int. 2021. 10.3238/arztebl.m2021.0121. 10.3238/arztebl.m2021.0121 PMC8245861 33602392

[13] Wessels MR. Streptococcal pharyngitis. N Engl J Med. 2011;364:648–55. 10.1056/NEJMcp1009126. 21323542 10.1056/NEJMcp1009126

[14] Oliver J, Malliya Wadu E, Pierse N, Moreland NJ, Williamson DA, Baker MG. Group A Streptococcus pharyngitis and pharyngeal carriage: a meta-analysis. Somily AM, editor. PLoS Negl Trop Dis. 2018;12:e0006335. 10.1371/journal.pntd.0006335. 29554121 10.1371/journal.pntd.0006335 PMC5875889

[15] Rick A-M, Zaheer HA, Martin JM. Clinical features of group a streptococcus in children with pharyngitis: carriers versus acute infection. Pediatr Infect Dis J. 2020;39:483–8. 10.1097/INF.0000000000002602. 32040013 10.1097/INF.0000000000002602 PMC8363186

[16] Barbieri E, di Chiara C, Costenaro P, Cantarutti A, Giaquinto C, Hsia Y, et al. Antibiotic prescribing patterns in paediatric primary care in Italy: findings from 2012–2018. Antibiot Multidisciplinary Digit Publishing Inst. 2022;11: 18. 10.3390/antibiotics11010018. 10.3390/antibiotics11010018 PMC8773435 35052895

[17] Pellegrino R, Timitilli E, Verga MC, Guarino A, Iacono ID, Scotese I, et al. Acute pharyngitis in children and adults: descriptive comparison of current recommendations from national and international guidelines and future perspectives. Eur J Pediatr Ger. 2023. 10.1007/s00431-023-05211-w. 10.1007/s00431-023-05211-w PMC10746578 37819417

[18] Milani GP, Rosa C, Tuzger N, Alberti I, Ghizzi C, Zampogna S, et al. Nationwide survey on the management of pediatric pharyngitis in Italian emergency units. Ital J Pediatr. 2023;49:114. 10.1186/s13052-023-01514-8. 37670391 10.1186/s13052-023-01514-8 PMC10481466

[19] Barbieri E, Donà D, Cantarutti A, Lundin R, Scamarcia A, Corrao G, et al. Antibiotic prescriptions in acute otitis media and pharyngitis in Italian pediatric outpatients. Ital J Pediatr. 2019;45:103. 10.1186/s13052-019-0696-9. 31420054 10.1186/s13052-019-0696-9 PMC6697973

[20] Page MJ, McKenzie JE, Bossuyt PM, Boutron I, Hoffmann TC, Mulrow CD, et al. The PRISMA 2020 statement: an updated guideline for reporting systematic reviews. BMJ. 2021;n71. 10.1136/bmj.n71. 10.1136/bmj.n71 PMC8005924 33782057

[21] Guyatt G, Oxman AD, Akl EA, Kunz R, Vist G, Brozek J, et al. GRADE guidelines: 1. Introduction-GRADE evidence profiles and summary of findings tables. J Clin Epidemiol. 2011;64:383–94. 10.1016/j.jclinepi.2010.04.026. 21195583 10.1016/j.jclinepi.2010.04.026

[22] Spinks A, Glasziou PP, Del Mar CB. Antibiotics for treatment of sore throat in children and adults. Cochrane Database Syst Rev. 2021;12:CD000023. 10.1002/14651858.CD000023.pub5. 34881426 10.1002/14651858.CD000023.pub5 PMC8655103

[23] Brink WR, Rammelkamp CH, Denny FW, Wannamaker LW. Effect in penicillin and aureomycin on the natural course of streptococcal tonsillitis and pharyngitis. Am J Med. 1951;10:300–8. 10.1016/0002-9343(51)90274-4. 14819035 10.1016/0002-9343(51)90274-4

[24] Brumfitt W, Slater JD. Treatment of acute sore throat with penicillin; a controlled trial in young soldiers. Lancet. 1957;272:8–11. 10.1016/s0140-6736(57)92432-7. 13386263 10.1016/s0140-6736(57)92432-7

[25] Chapple PA, Franklin LM, Paulett JD, Tuckman E, Woodall JT, Tomlinson AJ, et al. Treatment of acute sore throat in general practice; therapeutic trial, with observations on symptoms and bacteriology. Br Med J. 1956;1:705–8. 10.1136/bmj.1.4969.705. 13304304 10.1136/bmj.1.4969.705 PMC1979262

[26] De Meyere M, Mervielde Y, Verschraegen G, Bogaert M. Effect of penicillin on the clinical course of streptococcal pharyngitis in general practice. Eur J Clin Pharmacol. 1992;43:581–5. 10.1007/BF02284954. 1493837 10.1007/BF02284954

[27] Denny FW, Wannamaker LW, Hahn EO. Comparative effects of penicillin, aureomycin and terramycin on streptococcal tonsillitis and pharyngitis. Pediatrics. 1953;11:7–13. 13026346

[28] el-Daher NT, Hijazi SS, Rawashdeh NM, al-Khalil IA, Abu-Ektaish FM, Abdel-Latif DI. Immediate vs. delayed treatment of group a beta-hemolytic streptococcal pharyngitis with penicillin V. Pediatr Infect Dis J. 1991;10:126–30. 10.1097/00006454-199102000-00010. 1905799 10.1097/00006454-199102000-00010

[29] Krober MS, Bass JW, Michels GN. Streptococcal pharyngitis. Placebo-controlled double-blind evaluation of clinical response to penicillin therapy. JAMA. 1985;253:1271–4. 10.1001/jama.253.9.1271. 3918190 10.1001/jama.253.9.1271

[30] Macdonald TC, Watson IH. Sulphonamides and acute tonsillitis. Br Med J. 1951;1:323–6. 14821406 10.1136/bmj.1.4702.323 PMC2068133

[31] Middleton DB, D’Amico F, Merenstein JH. Standardized symptomatic treatment versus penicillin as initial therapy for streptococcal pharyngitis. J Pediatr. 1988;113:1089–94. 10.1016/s0022-3476(88)80588-2. 10.1016/s0022-3476(88)80588-2 3057159

[32] Zwart S, Sachs AP, Ruijs GJ, Gubbels JW, Hoes AW, de Melker RA. Penicillin for acute sore throat: randomised double blind trial of seven days versus three days treatment or placebo in adults. BMJ. 2000;320:150–4. 10.1136/bmj.320.7228.150. 10634735 10.1136/bmj.320.7228.150 PMC27262

[33] Zwart S, Rovers MM, de Melker RA, Hoes AW. Penicillin for acute sore throat in children: randomised, double blind trial. BMJ. 2003;327: 1324. 10.1136/bmj.327.7427.1324. 14656841 10.1136/bmj.327.7427.1324 PMC286321

[34] Dagnelie CF, van der Graaf Y, De Melker RA. Do patients with sore throat benefit from penicillin? A randomized double-blind placebo-controlled clinical trial with penicillin V in general practice. Br J Gen Pract. 1996;46:589–93. 8945796 PMC1239783

[35] Petersen K, Phillips RS, Soukup J, Komaroff AL, Aronson M. The effect of erythromycin on resolution of symptoms among adults with pharyngitis not caused by group a streptococcus. J Gen Intern Med. 1997;12:95–101. 10.1046/j.1525-1497.1997.00013.x. 9051558 10.1046/j.1525-1497.1997.00013.x PMC1497066

[36] Taylor B, Abbott GD, Kerr MM, Fergusson DM. Amoxycillin and co-trimoxazole in presumed viral respiratory infections of childhood: placebo-controlled trial. Br Med J. 1977;2:552–4. 10.1136/bmj.2.6086.552. 329949 10.1136/bmj.2.6086.552 PMC1631450

[37] Altamimi S, Khalil A, Khalaiwi KA, Milner RA, Pusic MV, Al Othman MA. Short-term late-generation antibiotics versus longer term penicillin for acute streptococcal pharyngitis in children. Cochrane Database Syst Rev. 2012:CD004872. 10.1002/14651858.CD004872.pub3. 10.1002/14651858.CD004872.pub3 PMC11984625 22895944

[38] van Driel ML, De Sutter AI, Thorning S, Christiaens T. Different antibiotic treatments for group a streptococcal pharyngitis. Cochrane Database Syst Rev. 2021;3:CD004406. 10.1002/14651858.CD004406.pub5. 33728634 10.1002/14651858.CD004406.pub5 PMC8130996

[39] Kuroki H, Ishiwada N, Inoue N, Ishikawa N, Suzuki H, Himi K, et al. Comparison of clinical efficacy between 3-day combined clavulanate/amoxicillin preparation treatment and 10-day amoxicillin treatment in children with pharyngolaryngitis or tonsillitis. J Infect Chemother. 2013;19:12–9. 10.1007/s10156-012-0444-1. 22760341 10.1007/s10156-012-0444-1

[40] Li P, Jiang G, Shen X. Evaluation of 3-day azithromycin or 5-day cefaclor in comparison with 10-day amoxicillin for treatment of tonsillitis in children. Can J Physiol Pharmacol. 2019;97:939–44. 10.1139/cjpp-2019-0087. 31365280 10.1139/cjpp-2019-0087

[41] Demoly P, Adkinson NF, Brockow K, Castells M, Chiriac AM, Greenberger PA, et al. International consensus on drug allergy. Allergy. 2014;69:420–37. 10.1111/all.12350. 24697291 10.1111/all.12350

[42] National Heart Foundation of New Zealand. Group A streptococcal sore throat management guideline. 2019 update. Auckland; 2019. https://www.heartfoundation.org.nz/resources/group-a-streptococcal-sore-throat-management. Accessed 5 May 2022.

[43] Gomes ER, Brockow K, Kuyucu S, Saretta F, Mori F, Blanca-Lopez N, et al. Drug hypersensitivity in children: report from the pediatric task force of the EAACI Drug Allergy Interest Group. Allergy. 2016;71:149–61. 10.1111/all.12774. 26416157 10.1111/all.12774

[44] Broyles AD, Banerji A, Barmettler S, Biggs CM, Blumenthal K, Brennan PJ, et al. Practical guidance for the evaluation and management of drug hypersensitivity: specific drugs. J Allergy Clin Immunol Pract. 2020;8:S16-116. 10.1016/j.jaip.2020.08.006. 33039007 10.1016/j.jaip.2020.08.006

[45] Romano A, Atanaskovic-Markovic M, Barbaud A, Bircher AJ, Brockow K, Caubet J-C, et al. Towards a more precise diagnosis of hypersensitivity to beta-lactams — an EAACI position paper. Allergy. 2020;75:1300–15. 10.1111/all.14122. 31749148 10.1111/all.14122

[46] Berbel D, González-Díaz A, López de Egea G, Càmara J, Ardanuy C. An overview of macrolide resistance in streptococci: prevalence, mobile elements and dynamics. Microorganisms. 2022;10: 2316. 10.3390/microorganisms10122316. 36557569 10.3390/microorganisms10122316 PMC9783990

[47] Paradise JL, Bluestone CD, Bachman RZ, Colborn DK, Bernard BS, Taylor FH, et al. Efficacy of tonsillectomy for recurrent throat infection in severely affected children. Results of parallel randomized and nonrandomized clinical trials. N Engl J Med. 1984;310:674–83. 10.1056/NEJM198403153101102. 6700642 10.1056/NEJM198403153101102

[48] Della Vecchia L, Passali FM, Coden E. Complications of adenotonsillectomy in pediatric age. Acta Biomed. 2020;91:48–53. 10.23750/abm.v91i1-S.9256. 32073561 10.23750/abm.v91i1-S.9256 PMC7947737

[49] Ng GJ, Tan S, Vu AN, Mar CBD, van Driel ML. Antibiotics for preventing recurrent sore throat. Cochrane Database Syst Reviews. 2015. 10.1002/14651858.cd008911.pub2. 10.1002/14651858.CD008911.pub2 PMC8860619 26171901

[50] Munck H, Jørgensen AW, Klug TE. Antibiotics for recurrent acute pharyngo-tonsillitis: systematic review. Eur J Clin Microbiol Infect Dis. 2018;37:1221–30. 10.1007/s10096-018-3245-3. 29651614 10.1007/s10096-018-3245-3

[51] Lildholdt T, Doessing H, Lyster M, Outzen K. The natural history of recurrent acute tonsillitis and a clinical trial of azithromycin for antibiotic prophylaxis. Clin Otolaryngol Allied Sci. 2003;28:371–3. 10.1046/j.1365-2273.2003.00728.x. 12871256 10.1046/j.1365-2273.2003.00728.x

[52] Brook I. Medical treatment of non-streptococcal recurrent tonsillitis. Am J Otolaryngol. 1989;10:227–33. 10.1016/0196-0709(89)90001-x. 2764234 10.1016/0196-0709(89)90001-x

[53] Brook I. Treatment of patients with acute recurrent tonsillitis due to group a beta-haemolytic streptococci: a prospective randomized study comparing penicillin and amoxycillin/clavulanate potassium. J Antimicrob Chemother. 1989;24:227–33. 10.1093/jac/24.2.227. 2676941 10.1093/jac/24.2.227

[54] Hung T-Y, Phuong LK, Grobler A, Tong SYC, Freeth P, Pelenda A, et al. Antibiotics to eradicate Streptococcus pyogenes pharyngeal carriage in asymptomatic children and adults: a systematic review. J Infect. 2024;88:106104. 10.1016/j.jinf.2024.01.003. 38360357 10.1016/j.jinf.2024.01.003

[55] Brook I, Hirokawa R. Treatment of patients with a history of recurrent tonsillitis due to group a beta-hemolytic streptococci. A prospective randomized study comparing penicillin, erythromycin, and clindamycin. Clin Pediatr (Phila). 1985;24:331–6. 10.1177/000992288502400606. 3888491 10.1177/000992288502400606

[56] Tanz RR, Poncher JR, Corydon KE, Kabat K, Yogev R, Shulman ST. Clindamycin treatment of chronic pharyngeal carriage of group a streptococci. J Pediatr. 1991;119:123–8. 10.1016/s0022-3476(05)81052-2. 2066844 10.1016/s0022-3476(05)81052-2

[57] Tanz RR, Shulman ST, Barthel MJ, Willert C, Yogev R. Penicillin plus rifampin eradicates pharyngeal carriage of group a streptococci. J Pediatr. 1985;106:876–80. 10.1016/s0022-3476(85)80229-8. 3889257 10.1016/s0022-3476(85)80229-8

[58] Aguilar A, Tinoco JC, Macias M, Huicho L, Levy J, Trujillo H, et al. Clinical and bacteriologic efficacy of amoxycillin b.d. (45 mg/kg/day) versus amoxycillin t.d.s (40 mg/kg/day) in children with group a beta-hemolytic streptococcal tonsillopharyngitis. J Chemother. 2000;12:396–405. 10.1179/joc.2000.12.5.396. 11128559 10.1179/joc.2000.12.5.396

[59] Nakao A, Hisata K, Fujimori M, Matsunaga N, Komatsu M, Shimizu T. Amoxicillin effect on bacterial load in group a streptococcal pharyngitis: comparison of single and multiple daily dosage regimens. BMC Pediatr. 2019;19:205. 10.1186/s12887-019-1582-8. 31226961 10.1186/s12887-019-1582-8 PMC6587266

[60] AIFA (Agenzia Italiana del Farmaco). Summary of product characteristics - amoxicillin. 2023. Available from: https://medicinali.aifa.gov.it/it/#/it/dettaglio/0000010236. Last accessed 11 Sep 2024.

[61] European Medicines Agency (EMA). Summary of product characteristics, labelling and package leaflet- amoxil- annex III. 2015. Available from: https://www.ema.europa.eu/en/medicines/human/referrals/amoxil. Last accessed 11 Sep 2024.

[62] Eslami ST, Nassirian A, Nassirian H, Hatami E, Sobhani E, Najibpour R. Comparing performance of amoxicillin and intramuscular benzathine penicillin in relieving manifestations of streptococcal pharyngitis in children. Ghana Med J. 2014;48:185–8. 10.4314/gmj.v48i4.3. 25709132 10.4314/gmj.v48i4.3 PMC4335436

[63] Bar-Yishay M, Yehoshua I, Bilitzky A, Press Y. Treatment outcomes of acute streptococcal tonsillitis according to antibiotic treatment. A retrospective analysis of 242,366 cases treated in the community. Eur J Gen Pract. 2022;28:142–9. 10.1080/13814788.2022.2083105. 35695024 10.1080/13814788.2022.2083105 PMC9225758

[64] Spurling GK, Del Mar CB, Dooley L, Foxlee R, Farley R. Delayed antibiotic prescriptions for respiratory infections. Cochrane Database Syst Rev. 2017;9:CD004417. 10.1002/14651858.CD004417.pub5. 28881007 10.1002/14651858.CD004417.pub5 PMC6372405

[65] Pichichero ME, Disney FA, Talpey WB, Green JL, Francis AB, Roghmann KJ, et al. Adverse and beneficial effects of immediate treatment of group a beta-hemolytic streptococcal pharyngitis with penicillin. Pediatr Infect Dis J. 1987;6:635–43. 10.1097/00006454-198707000-00004. 3302916 10.1097/00006454-198707000-00004

[66] Gerber MA, Randolph MF, DeMeo KK, Kaplan EL. Lack of impact of early antibiotic therapy for streptococcal pharyngitis on recurrence rates. J Pediatr. 1990;117:853–8. 10.1016/s0022-3476(05)80121-0. 2123239 10.1016/s0022-3476(05)80121-0

[67] Little P, Williamson I, Warner G, Gould C, Gantley M, Kinmonth AL. Open randomised trial of prescribing strategies in managing sore throat. BMJ. 1997;314:722–7. 10.1136/bmj.314.7082.722. 9116551 10.1136/bmj.314.7082.722 PMC2126131

[68] Miller KM, Carapetis JR, Beneden CAV, Cadarette D, Daw JN, Moore HC, et al. The global burden of sore throat and group a Streptococcus pharyngitis: a systematic review and meta-analysis. eClinicalMedicine Elsevier. 2022;48. 10.1016/j.eclinm.2022.101458. 10.1016/j.eclinm.2022.101458 PMC9124702 35706486

[69] Miller KM, Barnett TC, Cadarette D, Bloom DE, Carapetis JR, Cannon JW. Antibiotic consumption for sore throat and the potential effect of a vaccine against group a Streptococcus: a systematic review and modelling study. eBioMedicine. 2023;98. 10.1016/j.ebiom.2023.104864. Elsevier. 10.1016/j.ebiom.2023.104864 PMC10663680 37950997

