# Supplementary material for: Treatment of acute pharyngitis in children: an Italian intersociety consensus (SIPPS-SIP-SITIP-FIMP-SIAIP-SIMRI-FIMMG)
Source: Ital J Pediatr. 2024 Nov 6;50:235. doi: 10.1186/s13052-024-01789-5 (PMC11539554; doi:10.1186/s13052-024-01789-5)
Supplement: Supplementary file 1 — Additional file 1: Structure and Methodology of the Document. A thourough description of the structure and methodology of the consensus and is provided is this additional file. [file 13052_2024_1789_MOESM1_ESM.docx]

**STRUCTURE AND METHODOLOGY OF THE DOCUMENT**

**Document Structure**

This document aims to give the generalist paediatrician (family paediatrician, outpatient or hospital specialist) a practical and up-to-date tool. The document has been designed and structured according to three possible consultation methods:

**1. Summary of the recommendations:** they contain only the final practical indications relating to the various chapters and questions

**2. Pathology sections**: deal with the topic *in extenso*. Each chapter includes an introductory narrative part, with updated summaries regarding the definition of the pathology treated, the clinical framework and the diagnostic management, followed by a second part developed according to the GRADE methodology relating to antibiotic treatment in children with this pathology. We answered the questions based on research, analysis and synthesis of the scientific evidence and the "Evidence to the Decision" (EtD) process that explained the formulation of the recommendations and graded by grading it.

This document does not address issues related to children with comorbidities or underlying chronic disease, as specified below in the paragraph "Setting and reference population", management with therapies other than antibiotics and management of complications.

**3. Boxes, Figures and Tables in the Appendix:** allow a quick search and consultation of the references, the processes used to assess the evidence, the results obtained, and the formulation of recommendations.

# **Methods**

We decided to produce a Consensus Conference (CC) to provide for an analysis of the available evidence on the subject of the judicious use of antibiotic therapy in respiratory tract infections in developmental age, issues on which there may not be a general and complete sharing of opinions or conditions of uncertainty that can lead to inhomogeneity of behaviours on a clinical level, and the management aspects of assistance.

The Panel drew up the project and defined the general aims of the document and the specific objectives. Moreover, we established the time necessary for each phase and the coordination activity, the topics, the methods of consultation, research and selection of the studies, and, finally, the processes defining the Consensus by the Panel.

**Working Groups**

- Promoting Committee of the CC, which organised and directed the different stages of development of the CC;

- The Scientific and Technical Committee critically analyzed the literature, extracted and tabulated the relevant data, and elaborated the synthesis of the scientific literature. It also supported the formulation of recommendations according to the GRADE method; finally, it prepared the questionnaire for voting on the recommendations according to the Delphi method and analysed the results;

- Multidisciplinary and multi-professional panel or Jury panel (G), which elaborated the clinical questions, discussed the efficacy tests and formulated the recommendations, divided into subgroups by pathology in some phases of work;

- Writing group, which drafted the final text of the CC.

- A group of External Auditors of paediatricians and infectious disease specialists with expertise in the specific pathologies.

The Scientific Technical Committee and the Panel Committee held periodic meetings. We recorded the dates of the meetings and all preliminary versions of the document.

The multidisciplinary and multi-professional panel included paediatricians experts in infectious diseases, in developmental age, general paediatricians and paediatricians of free choice, experts in allergology, clinical pharmacology, microbiology, epidemiology and research methodology and parent representatives.

The members of the Groups have been indicated by the Scientific Societies of the various disciplines or by the reference Associations.

We used the Delphi method to reach an agreement on the topics selected and the strength of the recommendations^[[1]](#endnote-1)^.

The external auditors did not participate in any phase of the development and drafting of the document, nor did they vote for the recommendations.

**Consensus Conference Audience**

The primary users of the CC are family paediatricians, general paediatricians working in outpatient facilities and outpatient DEAs, paediatricians experts in infectious diseases, general practitioners (GPs) and Continuity of Care doctors, otolaryngology specialists, pulmonologists, immunologists, hygienists, pharmacologists, nurses and pharmacists involved in the management of children with respiratory infections.

Parents and caregivers are also users of the document.

**Setting and target population**

This CC refers to the outpatient setting in the context of Family Paediatrics, General Practitioners, in the hospital setting limited to outpatient and emergency room management, or to hospitalised patients in the clinical conditions considered in this document, with the exclusions reported below. The CC provides recommendations on the management of children aged one month to 18 years with the following respiratory infectious diseases: pharyngotonsillitis (FT), acute and chronic sinusitis, acute otitis media (OMA) and recurrent acute otitis media (OMAR), community-acquired pneumonia (CAP). Children less than one month of age or with chronic diseases or comorbidities are excluded (known primary or secondary cystic fibrosis and/or CFTR-pathies, primary ciliary dyskinesia, non-cystic fibrosis bronchiectasis, genetic diseases, known malformations of the cardio-respiratory system, neuromuscular diseases and other pre-existing chronic lung diseases, neoplasms, asthma, diabetes mellitus), for the treatment of which we refer to the specific guidelines by pathology.

**Formulation of definitions and questions**

We reported the definition of the treated pathology at the beginning of each chapter and based on the available literature.

We identified the questions and outcomes shared and discussed by the panel. The questions were formulated by the methodology group using the "PICO" model (Patient/Population [P]; Intervention/Indicator [I]; Comparator/Control [C]; Outcome [O]) and developed according to the GRADE method.

The panel identified the outcomes a priori and then ranked and voted (from 1 to 9) on them in terms of importance in the decision-making process

Only outcomes categorised as “critical” and “important” were considered for the literature review and, subsequently, the formulation of the recommendation.

In particular, we considered relevant only outcomes related to antibiotic therapy of respiratory infections in children, while we excluded those related to diagnostic management and therapies other than antibiotic therapies. We also excluded the treatment of the specific complications of the pathologies addressed

.

The subsets of the members' panel developed the clinical questions of the different pathologies.

**5.7. Research of Scientific Evidence**

We based the bibliographic search on the principle of hierarchical selection.

Firstly, summaries of evidence, Systematic Reviews, were sought.

We have also taken into account the most valid evidence-based LGs

The research was then completed, according to the principle of theoretical saturation, with the Primary Studies published after those included in the RS and with those considered relevant.

5.7.1. General Inclusion Criteria:

- Time limit of the search:

for RS: last ten years

for primary studies: from the date of closure of the bibliography of the RS included or, failing that, not older than ten years. We included the studies considered valid and relevant, found by manual research or indicated by experts regardless of the date of publication

- Publication language:

English, Italian. We also evaluated studies published in other languages if known to the authors, found by manual research or indicated by experts and considered valid and relevant.

- Population :

paediatric and adolescent patients, older than one month, without comorbidities or risk factors, suffering from the following respiratory infectious diseases: pharyngotonsillitis (FT), acute and chronic sinusitis, acute otitis media (OMA) and recurrent acute otitis media (OMAR), community-acquired pneumonia (CAP).

- Type of studies:

*Systematic Review, Meta-Analysis, Randomized Controlled Trial, Multicentre Study, Observational Study, Cohort Study, Longitudinal Study*

- Relevance to the clinical question

- Methodological validity, evaluated based on the minimum criteria described in the chapter "Analysis of scientific evidence".

The research strategy was discussed and agreed upon among the methodologists.

At least two authors carried out the research, evaluation of scientific evidence and data extraction; in case of disagreement, we decided after a discussion among the methodologists.

Research of Systematic Reviews and Primary Studies

**1. RS databases:** *Cochrane Library*, CDSR – *Cochrane Database of Systematic Reviews*, DARE – *Database of Abstract of Review of Effects In Cochrane Reviews*, *Other Reviews*, *Trials*

**2**. **PubMed** [http:// www.ncbi.nlm.nih.gov/pubmed](http://www.ncbi.nlm.nih.gov/pubmed)

**3. EMBASE** [*https://www.embase.com*](https://www.embase.com)

**4. SCOPUS** *https://www.scopus.com/*

**5. Manual Search**

**6. Bibliography from experts**

Keywords for Population, Intervention/Exposure Factor, Outcome and Search Strings

for each question are reported in the Supplementary files.

# **Analysis of scientific evidence**

We used validated checklists and criteria to perform the evidence analysis and evaluation.

We used the validated AGREE II tool^[[2]](#endnote-2)^]

For the evaluation of other consensus documents, we used the criteria defined by the SNLG^[[3]](#endnote-3)^

- Relevance of the topic
- Publication date < 3 years
- Multidisciplinary and multi-professional composition of the panel of experts
- Clear and detailed description of the methodology adopted and in line with the standards adopted by CNEC to assess the quality of scientific evidence

We used the validated tool AMSTAR 2 (*Assessment of Multiple Systematic Reviews*)^[[4]](#endnote-4)^.

Minimum score: overall judgement of high, moderate and low methodological quality.

We assessed any *bias* of the RCTs with the validated tool of the *Cochrane Collaboration* called "*Assessment of Risk of Bias*".^[[5]](#endnote-5)^

We used the Cochrane ROBINS-I tool for assessing the non-randomized controlled intervention trials, ^[[6]](#endnote-6)^

We used *Newcastle Ottawa Scales* to assess the observational studies: cohort, case-control, and cross-sectional*.*^[[7]](#endnote-7)^

We took the biases and the confounding factors into account to assess the quality of the studies.

Minimum validity criterion: absence of bias.

**GRADE Method**^[[8]](#endnote-8),^^[[9]](#endnote-9),^^[[10]](#endnote-10)^

**Grading the quality of the evidence**

| **Quality level** | **Meaning** | **Consequence** |
| --- | --- | --- |
| High | High degree of confidence in the results | It is unlikely that further studies will change confidence in effect estimation |
| Moderate | A fair degree of confidence in the results | Further studies may likely confirm or change confidence in effect estimation |
| Low | The results are hardly credible | More research is needed to obtain reliable estimates of the positive and negative effects of the intervention |
| Very low | The data examined is unreliable | You cannot rely on available effect estimates |

**Criteria for *upgrading* or *downgrading* the quality assessment (high, moderate, low, very low) of the tests**

| **Type of Tests**  **Randomized Controlled Trial = High**  **Observational study = low**  **Any other information = very low** | |
| --- | --- |
| **At.** Decrease  of the category  Attribution  (e.g. from "high" to  "moderate") | 1. Severe (-1 level) or very severe (-2 levels) limitations in the quality of study conduct  2. Inconsistency in results between different studies on the same question (-1 or -2 levels)  3. Some (-1 level) or important (-2 level) uncertainties about the direct transferability of results (*directness*)  4. Inaccuracy or insufficient data (*sparse data*) (-1 or -2 levels)  5. Possibility of selective publication and *reporting bias* (-1 or -2 levels) |
| **B.** Increase  of the category  Attribution  (e.g. from "low"  to "moderate") | 1. Strong intervention-outcome association, i.e. with relative risk >2 (<0.5), based on concordant evidence from two or more observational studies, without any plausible confounding factor (+1 level)  2. Very strong intervention-outcome association, i.e. with relative risk >5 (<0.2) (+2 levels)  3. Presence of a dose-response gradient (+1 level)  4. All possible confounding factors that could have altered the effect estimates would have reduced the observed effect (+1 level) |

**Outline of recommendations**

Recommendations Strength Assessments

Strong recommendation *for* action

Weak recommendation *in favour* of action

Weak recommendation *against* intervention

Strong Recommendation *Against* Intervention

**Determinants of the strength of the recommendation**

1) *Balance between desirable and undesirable effects*

If the difference between the magnitude of the desired and undesirable outcomes is large, the recommendation is strong (for or against the intervention). If the difference is small, the recommendation is weak.

2) *Overall quality of evidence for the outcomes considered*

The higher the quality of evidence for the outcomes considered, the stronger a recommendation.

3) *Values and preferences*

The more the assigned values and preferences diverge or the greater the uncertainty is, the greater the chance that the recommendation will be weak.

4) Costs (resource allocation)

The higher the costs of an intervention (i.e. the more resources consumed), the less likely it is to consider a strong recommendation.

Note. The formulation of the recommendations has been the subject of great attention and in-depth discussions, both on the substantive and formal aspects. We noted that, based on the GRADE methodology, even in cases where good or at least moderate quality scientific evidence was not available, the authors tried to formulate shared recommendations, appropriate to the severity of the disease considered, taking into account the risks and benefits: consequently, it is not unusual, nor wrong, to formulate strong recommendations based on low-quality evidence or even based on expert opinion.

**Approval of recommendations**

We used the Delphi method with a blinded questionnaire

to vote on the recommendations.

We prepared five possible answers: *strongly agree, agree*, *neither agree nor disagree, disagree, strongly disagree.*

There are no unambiguous criteria for approving recommendations.

Informally, we considered that in several documents of good methodological quality, the panel approved the recommendations with a percentage of agreement equal to 70-75% *("strongly agree", "agree*").

We requested the reasons in case of *"neither agree nor disagree", "disagree", and "strongly disagree" answers*.

In any case, all the comments of the disagreeing votes were recorded and carefully considered, both on the content of the recommendation and on the formal correctness and clarity of exposition.

**GRADE-ADOLOPMENT**

The GRADE-ADOLOPMENT method is an evolution of the GRADE method that allows you to assess whether you can adapt to your context or adopt existing LG recommendations published to answer the PICO1 questions.

In this paper, we evaluated the possibility of adopting the recommendations of some LGs on the therapy of OMA and OMAR.

**Presentation, participation of Users and Users**

We presented a non-final version of this CC to the Jury of experts, nurses' and parents' associations. After in-depth discussion, the comments and observations collected, if considered appropriate, were incorporated into the document.

The Jury then defined the conclusions, and the writing committee drafted the preliminary consensus document.

The document was internally reviewed and approved by all panel members.

Four external reviewers assessed the document.

The authors approved the final draft in October 2023

**Software**

We used the RevMan 5.4.1 software^[[11]](#footnote-1)^, Nordic Cochrane Centre, The Cochrane Collaboration, 2014, to evaluate the methodological quality of the RCTs, the meta-analyses and related figures.

We used the GRADEpro GDT software, developed by the GRADE Working Group, for the overall quality of the evidence and the related tables.

**Update**

We will update the document after three years or in case of publication of new evidence that leads to changes to the recommendations.

**Implementation**

We will present the document at scientific meetings, and courses, as well as paediatric *forums and mailing lists*; in particular, we will give widespread information to family paediatricians, general paediatricians working in outpatient facilities and outpatient DEAs, paediatricians infectious disease specialists, general practitioners (GPs) and Continuity of Care doctors.

**Financing**

The SIPPS covered the costs for the document's drafting and publication (panel meetings, editing, printing and distribution). All the authors worked free of charge.

**Conflict of interest**

Each of the members of the working groups signed a declaration on possible conflicts of interest (CI) in the preliminary stages of the project and at the end of the project.

Management of any CIs:

- the members of the methodology team and the external auditors did not have CI;
- the authors with any ICs did not participate, therefore, in the systematic review of the evidence, but participated in all the other phases of implementations, contributing as far as their competence was concerned;
- the methodology group and the authors without ICs checked the correctness and consistency of each part of the document and, in particular, of the recommendations;
- each author could vote, express and justify any disagreement anonymously;
- we discussed the results of the votes and, in particular, the reasons for any disagreements collectively for the final version of the conclusions and recommendations.

1. Boulkedid R, Abdoul H, Loustau M, et al. Using and Reporting the Delphi Method for Selecting Healthcare Quality Indicators: A Systematic Review. PLoS One. 2011;6:e20476 [↑](#endnote-ref-1)
2. Brouwers M, Kho ME, Browman GP, et al. for the AGREE Next Steps Consortium. AGREE II: Advancing guideline development, reporting and evaluation in healthcare. Can Med Assoc J. 2010. Available online July 5, 2010 [↑](#endnote-ref-2)
3. SNLG. Good clinical-care practices. Available in <https://snlg.iss.it/?cat=4> (last accessed 24-07-2021) [↑](#endnote-ref-3)
4. Shea BJ, Reeves BC, Wells G, et al. AMSTAR 2: a critical appraisal tool for systematic reviews that include randomised or non-randomised studies of healthcare interventions, or both. BMJ. 2017; 358:J4008 [↑](#endnote-ref-4)
5. Higgins, J.P.T.; Thomas, J.; Chandler, J.; Cumpston, M.; Li, T.; Page, M.J.; Welch, V.A. (Eds.). Cochrane Handbook for Systematic Reviews of Interventions Version 6.2 (Updated February 2021); Cochrane: 2021. Available online: www.training.cochrane.org/handbook (accessed on 15 July 2022). [↑](#endnote-ref-5)
6. Sterne, J.A.C.; Hernán, M.A.; Reeves, B.C.; Savović, J.; Berkman, N.D.; Viswanathan, M.; Henry, D.; Altman, D.G.; Ansari, M.T.; Boutron, I.; et al. ROBINS-I: A tool for assessing risk of bias in non-randomized studies of interventions. BMJ 2016, 355, i4919e [↑](#endnote-ref-6)
7. Wells GA, Shea B, O'Connell D, et al. The Newcastle-Ottawa Scale (NOS) for assessing the quality of nonrandomized studies in meta-analyses, 2012. Available at: <http://wwwohrica/programs/clinical_epidemiology/oxfordasp> (last accessed 24-07-2021) [↑](#endnote-ref-7)
8. Schünemann HJ, Oxman AD, Brozek J, et al. GRADE Working Group. Grading quality of evidence and strength of recommendations for diagnostic tests and strategies. BMJ. 2008; 336:1106-10 [↑](#endnote-ref-8)
9. Guyatt GH, Oxman AD, Kunz R, et al. GRADE Working Group. Going from evidence to recommendations. BMJ. 2008;336:1049-51 [↑](#endnote-ref-9)
10. Guyatt GH, Oxman AD, Kunz R, et al. GRADE working group. Incorporating considerations of resource use into grading recommendations. BMJ. 2008;336:1170-73 [↑](#endnote-ref-10)
11. [↑](#footnote-ref-1)
