# Supplementary material for: Treatment of acute pharyngitis in children: an Italian intersociety consensus (SIPPS-SIP-SITIP-FIMP-SIAIP-SIMRI-FIMMG)
Source: Ital J Pediatr. 2024 Nov 6;50:235. doi: 10.1186/s13052-024-01789-5 (PMC11539554; doi:10.1186/s13052-024-01789-5)
Supplement: Supplementary file 2 — Additional file 2: Search strategy. A thourough description of the search strategy is provided in this addiotional file, including the PICO questions, Keywords and the search strings used for each database. The flow diagrams of literature search and data extraction for systematic reviews and clinical studies are provided as Figure A2.1 and A2.2. [file 13052_2024_1789_MOESM2_ESM.docx]

**Additional file 2- Search strategy**

**PICOs**

**Question 1.** **Should Group A β-haemolytic streptococcus (GABHS) pharyngotonsillitis be treated with antibiotics?**

**P** children with GABHS pharyngotonsillitis

**I** antibiotic therapy

**C** no therapy or simptomatic therapy alone

**O1** severity and duration of symptoms

**O2** relapsing pharyngotonsillitis

**O3** suppurative complications

**O4** non suppurative complications (acute rheumatic fever [ARF], acute post-streptococcal glomerulonephritis [APSGN])

**Question 2.** **Should amoxicillin be considered the antibiotic of choice in the treatment of GABHS pharyngotonsillitis besides penicillin V?**

**P** children with GABHS pharyngotonsillitis

**I** antibiotic therapy (amoxicillin-clavulanate, macrolides, cephalosporyns)

**C** amoxicillin

**O1** severity and duration of symptoms

**O2** relapsing pharyngotonsillitis

**O3** suppurative complications

**O4** non suppurative complications (acute rheumatic fever [ARF], acute post-streptococcal glomerulonephritis [APSGN])

**Question 3.** **Should the duration of antibiotic therapy for GABHS pharyngotonsillitis be shorter than 10 days?**

**P** children with GABHS pharyngotonsillitis

**I** antibiotic therapy < 10 days

**C** antibiotic therapy > 10 days

**O1** severity and duration of symptoms

**O2** relapsing pharyngotonsillitis

**O3** suppurative complications

**O4** non suppurative complications (acute rheumatic fever [ARF], acute post-streptococcal glomerulonephritis [APSGN])

**Question 4.** **In children allergic to penicillin, which antibiotics can be administered for the treatment of GABHS pharyngotonsillitis?**

**P** children with GABHS pharyngotonsillitis and penicillin allergy

**I** macrolides, cephalosporyns

**C** amoxicillin

**O1** severity and duration of symptoms

**O2** relapsing pharyngotonsillitis

**O3** suppurative complications

**O4** non suppurative complications (acute rheumatic fever [ARF], acute post-streptococcal glomerulonephritis [APSGN])

**Question 5:** **Which antibiotic(s) should be recommended as first-choice therapy for relapsing GABHS pharyngotonsillitis despite several courses of amoxicillin?**

**P** children with relapsing GABHS pharyngotonsillitis after amoxicillin (50 mg/kg/die)

**I1** Amoxicillin 80-90 mg/kg/die

**I2** Amoxicillin-clavulanate

**I3** cephalosporin

**I4** macrolides

**I5** other (i.e. clindamycin, Rifampicin, Cotrimoxazole, etc.)

**C** Phenoxymethylpenicillin (penicillin V)

**O1** severity and duration of symptoms

**O2** relapsing pharyngotonsillitis

**O3** suppurative complications

**O4** non suppurative complications (acute rheumatic fever [ARF], acute post-streptococcal glomerulonephritis [APSGN])

**Question 6:** **Which is the appropriate dosage of amoxicillin in the treatment of GABHS pharyngotonsillitis?**

**P** children with GABHS pharyngotonsillitis

**I** amoxicillin 50 mg/kg/die divided in 2 doses q12h

**C** amoxicillin 50 mg/kg/die divided in 3 doses q8h

**O1** severity and duration of symptoms

**O2** relapsing pharyngotonsillitis

**O3** suppurative complications

**O4** non suppurative complications (acute rheumatic fever [ARF], acute post-streptococcal glomerulonephritis [APSGN])

**Question 7: May parenteral antibiotics, specifically intramuscular benzathine-penicillin, be recommended as treatment alternative to oral amoxicillin in selected GABHS pharyngotonsillitis patients?**

**P** children with GABHS pharyngotonsillitis

**I** parenteral antibiotictheraoy, specifically intramuscular administration (benzathine penicillin G, ceftriaxone)

**C** oral amoxicillin

**O1** severity and duration of symptoms

**O2** relapsing pharyngotonsillitis

**O3** suppurative complications

**O4** non suppurative complications (acute rheumatic fever [ARF], acute post-streptococcal glomerulonephritis [APSGN])

**Question 8.** **Is it necessary to treat non-streptococcal bacterial pharyngotonsillitis (Fusobacterium spp., other anaerobes, Staphylococcus aureus, etc.) with antibiotics?**

**P** children with non-GABHS pharyngotonsillitis

**I** antibiotic therapy

**C** no therapy or simptomatic therapy alone

**O1** severity and duration of symptoms

**O2** relapsing pharyngotonsillitis

**O3** suppurative complications

**KEYWORDS**

**Population**

*PUBMED*

Child* OR pediatric*

Pharingytis

sore throat

"tonsillitis"[MeSH Terms] OR tonsillitis[Text Word]

"streptococcus pyogenes"[MeSH Terms] OR streptococcus pyogenes[Text Word]

*EMBASE*

'tonsillitis'/exp OR tonsillitis

('streptococcus'/exp OR streptococcus) AND pyogenes

*SCOPUS*

pharyngitis

group a streptococcus beta hemolyticus

bacterial tonsillitis

streptococcus pyogenes

streptococcal  tonsillopharyngitis

children

pediatrics

sore throat

fusobacterium

staphylococcus

*COCHRANE LIBRARY*

sore throat pediatric

children or pediatrics

pharyngitis

streptococcal tonsillopharyngitis

Streptococcal pharyngitis

Recurrent Streptococcal pharyngitis

Tonsillitis

penicillin allergy

**Comparison/ Exposure factors**

*PUBMED*

"anti-bacterial agents"[All Fields] OR "anti-bacterial agents"[MeSH Terms] OR antibiotic[Text Word]

"amoxicillin"[MeSH Terms] OR amoxicillin[Text Word]

"amoxicillin-potassium clavulanate combination"[MeSH Terms] OR amoxicillin clavulanic acid[Text Word]

"cephalosporins"[MeSH Terms] OR cephalosporin[Text Word]

"macrolides"[MeSH Terms] OR macrolide[Text Word]

"azithromycin"[MeSH Terms] OR azitrocin[Text Word],

"clarithromycin"[MeSH Terms] OR clarithromycin[Text Word],

eritro"erythromycin"[MeSH Terms] OR erythromycin[Text Word]

"clindamycin"[MeSH Terms] OR clindamycin[Text Word]

"Rifampin"[Mesh]

"trimethoprim, sulfamethoxazole drug combination"[MeSH Terms] OR cotrimoxazole[Text Word]

"Injections, Intramuscular"[Mesh]

"Penicillin V"[Mesh]

"Penicillin G Benzathine"[Mesh]

"ceftriaxone"[MeSH Terms] OR ceftriaxon[Text Word]

"anti-inflammatory agents"[All Fields] OR "anti-inflammatory agents"[MeSH Terms]

"Anti-Inflammatory Agents, Non-Steroidal"[Mesh]

"acetaminophen"[MeSH Terms] OR paracetamol[Text Word]

"administration and dosage" [Subheading]

*EMBASE*

'antiinfective agent'

'amoxicillin'

'amoxicillin plus clavulanic acid'

'cephalosporin derivative'

'macrolide'

'clindamycin'

'cotrimoxazole'

'intramuscular drug administration'

'parenteral drug administration'

'penicillin V'

'benzathine penicillin'

'ceftriaxone'

symptomatic AND therapy

'antiinflammatory agent'

'paracetamol'

'drug dose'

*SCOPUS*

antibiotic

antibiotic  therapy

amoxicillin

dose

cephalosporins

macrolide

intramuscular

*COCHRANE LIBRARY*

amoxicillin

intravenous or intramuscular

dose

interval

every 12 hours

every 8 hours

treatment

**Outcomes**

*PUBMED*

"complications"[Subheading] OR complications[Text Word]

"recurrence"[MeSH Terms] OR relapse[Text Word]

"Suppuration"[Mesh]

"rheumatic fever"[MeSH Terms] OR rheumatic fever[Text Word]

"hospitalization"[MeSH Terms] OR hospitalization[Text Word]

*EMBASE*

'complications'/exp OR complications

'recurrent disease', 'recurrence risk'

'suppuration'

'rheumatic fever'

hospitalization

*SCOPUS*

recurrence

*COCHRANE LIBRARY*

(none)

**Search strings for systematic review**

**PUBMED**

#1

(("pharyngitis"[MeSH Terms] OR "tonsillitis"[MeSH Terms] OR "sore throat"[All Fields] OR "tonsillophar*"[All Fields]) AND ("streptococc*"[All Fields] OR "pyogen*"[All Fields] OR "group A"[All Fields]) AND ("anti-bacterial agents"[MeSH Terms] OR "antibiotic*"[All Fields] OR "therapy"[All Fields]) AND ("child*"[All Fields] OR "pediatr*"[All Fields]) AND ("complicat*"[All Fields] OR "recur*"[All Fields] OR "suppur*"[All Fields] OR "rheumatic"[All Fields])) AND ((y_10[Filter]) AND (meta-analysis[Filter] OR review[Filter] OR systematicreview[Filter]) AND (english[Filter]))

#2

(("pharyngitis"[MeSH Terms] OR "tonsillitis"[MeSH Terms] OR "sore throat"[All Fields] OR "tonsillophar*"[All Fields]) AND ("streptococc*"[All Fields] OR "pyogen*"[All Fields] OR "group A"[All Fields]) AND ("anti-bacterial agents"[MeSH Terms] OR "antibiotic*"[All Fields] OR "therapy"[All Fields]) AND (“durat*” OR “day” OR “days”)) AND ("child*"[All Fields] OR "pediatr*"[All Fields]) AND ("complicat*"[All Fields] OR "recur*"[All Fields] OR "suppur*"[All Fields] OR "rheumatic"[All Fields]) AND ("amoxicillin"[MeSH Terms] OR "penicillin") AND ((y_10[Filter]) AND (meta-analysis[Filter] OR review[Filter] OR systematicreview[Filter]) AND (english[Filter]))

#3

((("child"[MeSH Terms] OR "child"[All Fields] OR "children"[All Fields] OR "child s"[All Fields] OR "children s"[All Fields] OR "childrens"[All Fields] OR "childs"[All Fields]) AND ("pharyngitis"[MeSH Terms] OR "pharyngitis"[All Fields] OR "pharyngitides"[All Fields])) OR (("palatine tonsil"[MeSH Terms] OR ("palatine"[All Fields] AND "tonsil"[All Fields]) OR "palatine tonsil"[All Fields] OR "tonsil"[All Fields] OR "tonsils"[All Fields] OR "tonsilitis"[All Fields] OR "tonsillitis"[MeSH Terms] OR "tonsillitis"[All Fields] OR "tonsillitides"[All Fields] OR "tonsills"[All Fields]) AND ("streptococcus pyogenes"[MeSH Terms] OR ("streptococcus"[All Fields] AND "pyogenes"[All Fields]) OR "streptococcus pyogenes"[All Fields]) AND ("benzylpenicillins"[All Fields] OR "penicillin g"[MeSH Terms] OR "penicillin g"[All Fields] OR "benzylpenicillin"[All Fields] OR "penicilline"[All Fields] OR "penicillines"[All Fields] OR "penicillins"[MeSH Terms] OR "penicillins"[All Fields] OR "penicillin"[All Fields]) AND ("allergie"[All Fields] OR "hypersensitivity"[MeSH Terms] OR "hypersensitivity"[All Fields] OR "allergies"[All Fields] OR "allergy"[All Fields] OR "allergy and immunology"[MeSH Terms] OR ("allergy"[All Fields] AND "immunology"[All Fields]) OR "allergy and immunology"[All Fields]) AND ("complications"[Subheading] OR complications[Text Word] OR "recurrence"[MeSH Terms] OR relapse[Text Word] OR "Suppuration"[Mesh] OR "rheumatic fever"[MeSH Terms] OR rheumatic fever[Text Word] OR "hospitalization"[MeSH Terms] OR hospitalization[Text Word])) AND ((y_10[Filter]) AND (meta-analysis[Filter] OR systematicreview[Filter]) AND (english[Filter]))

#4

((("pharyngitis"[MeSH Terms] OR "pharyngitis"[All Fields] OR "pharyngitides"[All Fields]) AND ("benzylpenicillins"[All Fields] OR "penicillin g"[MeSH Terms] OR "penicillin g"[All Fields] OR "benzylpenicillin"[All Fields] OR "penicilline"[All Fields] OR "penicillines"[All Fields] OR "penicillins"[MeSH Terms] OR "penicillins"[All Fields] OR "penicillin"[All Fields]) AND ("allergie"[All Fields] OR "hypersensitivity"[MeSH Terms] OR "hypersensitivity"[All Fields] OR "allergies"[All Fields] OR "allergy"[All Fields] OR "allergy and immunology"[MeSH Terms] OR ("allergy"[All Fields] AND "immunology"[All Fields]) OR "allergy and immunology"[All Fields])) OR (("streptococcus"[MeSH Terms] OR "streptococcus"[All Fields] OR "streptococcal"[All Fields]) AND ("pharyngitis"[MeSH Terms] OR "pharyngitis"[All Fields] OR "pharyngitides"[All Fields]) AND "penicillin amoxicillin"[All Fields] AND ("allergie"[All Fields] OR "hypersensitivity"[MeSH Terms] OR "hypersensitivity"[All Fields] OR "allergies"[All Fields] OR "allergy"[All Fields] OR "allergy and immunology"[MeSH Terms] OR ("allergy"[All Fields] AND "immunology"[All Fields]) OR "allergy and immunology"[All Fields]))) AND ((y_10[Filter]) AND (meta-analysis[Filter] OR systematicreview[Filter]) AND (english[Filter]

#5

("tonsillitis"[MeSH Terms] OR "tonsillitis"[All Fields] OR "pharyngitis"[MeSH Terms] OR "pharyngitis"[All Fields]) AND ("streptococcus pyogenes"[MeSH Terms] OR ("streptococcus"[All Fields]) AND ("recurrence"[MeSH Terms] OR "recurrence"[All Fields]) AND ("penicillin v"[MeSH Terms] OR "penicillin v"[All Fields] OR "amoxicillin-potassium clavulanate combination"[MeSH Terms] OR ("amoxicillin-potassium"[All Fields] OR "macrolides"[MeSH Terms] OR "macrolides"[All Fields] OR "cephalosporins"[MeSH Terms] OR "cephalosporins"[All Fields] OR "clindamycin"[MeSH Terms] OR "clindamycin"[All Fields] OR "clindamycine"[All Fields] OR "rifampin"[MeSH Terms] OR "rifampin"[All Fields] OR "trimethoprim, sulfamethoxazole drug combination"[MeSH Terms] OR "trimethoprim, sulfamethoxazole drug combination"[All Fields]) AND ((y_10[Filter]) AND (meta-analysis[Filter] OR systematicreview[Filter]) AND (allchild[Filter]))

#6

(("pharyngitis"[MeSH Terms] OR "pharyngitis"[All Fields] OR "pharyngitides"[All Fields] OR ("palatine tonsil"[MeSH Terms] OR ("palatine"[All Fields] AND "tonsil"[All Fields]) OR "palatine tonsil"[All Fields] OR "tonsil"[All Fields] OR "tonsils"[All Fields] OR "tonsilitis"[All Fields] OR "tonsillitis"[MeSH Terms] OR "tonsillitis"[All Fields] OR "tonsillitides"[All Fields] OR "tonsills"[All Fields]) OR ("pharyngitis"[MeSH Terms] OR "pharyngitis"[All Fields] OR ("sore"[All Fields] AND "throat"[All Fields]) OR "sore throat"[All Fields])) AND ("amoxicillin"[MeSH Terms] OR "amoxicillin"[All Fields] OR "amoxicilline"[All Fields] OR "amoxicillins"[All Fields] OR ("cephalosporine"[All Fields] OR "cephalosporines"[All Fields] OR "cephalosporins"[MeSH Terms] OR "cephalosporins"[All Fields] OR "cephalosporin"[All Fields]) OR ("macrolid"[All Fields] OR "macrolides"[MeSH Terms] OR "macrolides"[All Fields] OR "macrolide"[All Fields] OR "macrolids"[All Fields])) AND ("child"[MeSH Terms] OR "child"[All Fields] OR "children"[All Fields] OR "child s"[All Fields] OR "children s"[All Fields] OR "childrens"[All Fields] OR "childs"[All Fields] OR ("paediatrics"[All Fields] OR "pediatrics"[MeSH Terms] OR "pediatrics"[All Fields] OR "paediatric"[All Fields] OR "pediatric"[All Fields])) AND ("intraveneous"[All Fields] OR "intraveneously"[All Fields] OR "intravenous"[All Fields] OR "intravenously"[All Fields] OR "intramuscular"[All Fields])) AND (y_10[Filter])

#7

(("palatine tonsil"[MeSH Terms] OR ("palatine"[All Fields] AND "tonsil"[All Fields]) OR "palatine tonsil"[All Fields] OR "tonsil"[All Fields] OR "tonsils"[All Fields] OR "tonsilitis"[All Fields] OR "tonsillitis"[MeSH Terms] OR "tonsillitis"[All Fields] OR "tonsillitides"[All Fields] OR "tonsills"[All Fields] OR ("pharyngitis"[MeSH Terms] OR "pharyngitis"[All Fields] OR "pharyngitides"[All Fields]) OR ("pharyngitis"[MeSH Terms] OR "pharyngitis"[All Fields] OR ("sore"[All Fields] AND "throat"[All Fields]) OR "sore throat"[All Fields]) OR "BACTERIAL TONSILLITIS"[All Fields] OR ("streptococcus"[MeSH Terms] OR "streptococcus"[All Fields]) OR ("fusobacterium"[MeSH Terms] OR "fusobacterium"[All Fields]) OR ("staphylococcu"[All Fields] OR "staphylococcus"[MeSH Terms] OR "staphylococcus"[All Fields])) AND "child*"[All Fields]) AND ((y_10[Filter]) AND (meta-analysis[Filter] OR systematicreview[Filter]))

#8

("pharyngitis"[MeSH Terms] OR "pharyngitis"[All Fields] OR "pharyngitides"[All Fields]) AND ("amoxicillin"[MeSH Terms] OR "amoxicillin"[All Fields] OR "amoxicilline"[All Fields] OR "amoxicillins"[All Fields]) AND ("child"[MeSH Terms] OR "child"[All Fields] OR "children"[All Fields] OR "child s"[All Fields] OR "children s"[All Fields] OR "childrens"[All Fields] OR "childs"[All Fields] OR ("paediatrics"[All Fields] OR "pediatrics"[MeSH Terms] OR "pediatrics"[All Fields] OR "paediatric"[All Fields] OR "pediatric"[All Fields]) OR ("paediatrics"[All Fields] OR "pediatrics"[MeSH Terms] OR "pediatrics"[All Fields] OR "paediatric"[All Fields] OR "pediatric"[All Fields]))

**EMBASE**

#1

('child'/exp OR 'streptococcal pharyngitis'/exp) AND 'penicillin allergy'/exp AND ('macrolide'/exp OR 'cephalosporin'/exp) AND 'amoxicillin'/exp AND ('relapse'/exp OR 'rheumatic fever'/exp OR 'retropharyngeal abscess'/exp OR 'hospitalization'/exp) AND ('systematic review'/exp OR 'meta analysis'/exp)

#2

('tonsillitis'/exp OR tonsillitis OR 'pharyngitis'/exp OR pharyngitis) AND ('streptococcus'/exp OR streptococcus) AND pyogenes AND ([cochrane review]/lim OR [systematic review]/lim OR [meta analysis]/lim) AND [2012-2022]/py

#3

('streptococcal tonsillopharyngitis' OR (streptococcal AND ('tonsillopharyngitis'/exp OR tonsillopharyngitis))) AND ('antibiotic therapy'/exp OR 'antibiotic therapy' OR (('antibiotic'/exp OR antibiotic) AND ('therapy'/exp OR therapy))) AND ('child'/exp OR child) ('streptococcal tonsillopharyngitis' OR (streptococcal AND ('tonsillopharyngitis'/exp OR tonsillopharyngitis))) AND ('antibiotic therapy'/exp OR 'antibiotic therapy' OR (('antibiotic'/exp OR antibiotic) AND ('therapy'/exp OR therapy))) AND ('child'/exp OR child)

#4

('pharyngitis'/exp OR pharyngitis OR 'tonsillitis'/exp OR tonsillitis OR 'sore throat'/exp OR 'sore throat' OR (sore AND ('throat'/exp OR throat))) AND ('amoxicillin'/exp OR amoxicillin) AND ('children'/exp OR children OR 'pediatrics'/exp OR pediatrics) AND intramuscular AND ([cochrane review]/lim OR [systematic review]/lim) AND [2012-2022]/py

#5

('tonsilitis'/exp OR tonsilitis OR 'sore throat'/exp OR 'sore throat' OR 'bacterial tonsillitis' OR 'staphylococcus'/exp OR staphylococcus OR 'fusobacterium'/exp OR fusobacterium) AND ('child'/exp OR child) AND ([cochrane review]/lim OR [systematic review]/lim OR [meta analysis]/lim) AND [2012-2022]/py

**SCOPUS**

#1

TITLE-ABS-KEY ( pharyngitis OR group AND a AND streptococcus AND beta AND hemolyticus OR bacterial AND tonsillitis OR streptococcus AND pyogenes OR antibiotic ) AND PUBYEAR > 2011 AND ( LIMIT-TO ( DOCTYPE , "re" ) ) AND ( LIMIT-TO ( LANGUAGE , "English" ) )

#2

( TITLE-ABS-KEY ( pharyng* )  AND  TITLE-ABS-KEY ( streptococ*  AND pyogenes )  AND  TITLE-ABS-KEY ( antibiotic  AND therapy ) )  AND  PUBYEAR  >  2011  AND  ( LIMIT-TO ( DOCTYPE ,  "re" ) )

#3

TITLE-ABS-KEY ( pharyngitis  AND  streptococcus  AND  pyogenes  AND  recurrence )  AND  PUBYEAR  >  2011  AND  ( LIMIT-TO ( LANGUAGE ,  "English"))

#4

( amoxicillin  AND  dose  AND  streptococcal  AND tonsillopharyngitis  AND  children )  AND  ( LIMIT-TO ( PUBYEAR ,  2021 )  OR  LIMIT-TO ( PUBYEAR ,  2020 )  OR  LIMIT TO ( PUBYEAR ,  2016 ) )  AND  ( LIMIT-TO ( DOCTYPE ,  "re" ) )

#5

( ( streptococcus AND pyogenes OR streptococcal OR gabhs ) AND ( tonsillitis OR pharyngit* OR nasopharyngit* OR rhinopharyngit* OR tonsillopharyngit* OR sore AND throat ) AND ( amoxicillin OR beta-lactam OR amoxycillin OR ampicillin OR betalactam ) ) AND ( LIMIT-TO ( PUBYEAR , 2022 ) OR LIMIT-TO ( PUBYEAR , 2021 ) OR LIMIT-TO ( PUBYEAR , 2020 ) OR LIMIT-TO ( PUBYEAR , 2019 ) OR LIMIT-TO ( PUBYEAR , 2018 ) OR LIMIT-TO ( PUBYEAR , 2017 ) OR LIMIT-TO ( PUBYEAR , 2016 ) OR LIMIT-TO ( PUBYEAR , 2015 ) OR LIMIT-TO ( PUBYEAR , 2014 ) OR LIMIT-TO ( PUBYEAR , 2013 ) OR LIMIT-TO ( PUBYEAR , 2012 ) ) AND ( LIMIT-TO ( LANGUAGE , "English" ) ) AND ( LIMIT-TO ( DOCTYPE , "re" ) )

#6

pharyngitis OR tonsillitis OR sore AND throat ) AND ( amoxicillin OR cephalosporins OR macrolide ) AND ( children OR pediatrics ) AND intramuscular AND ( LIMIT-TO ( PUBYEAR , 2022 ) OR LIMIT-TO ( PUBYEAR , 2013 ) OR LIMIT-TO ( PUBYEAR , 2021 ) OR LIMIT-TO ( PUBYEAR , 2020 ) OR LIMIT-TO ( PUBYEAR , 2019 ) OR LIMIT-TO ( PUBYEAR , 2018 ) OR LIMIT-TO ( PUBYEAR , 2017 ) OR LIMIT-TO ( PUBYEAR , 2016 ) OR LIMIT-TO ( PUBYEAR , 2015 ) OR LIMIT-TO ( PUBYEAR , 2014 ) OR LIMIT-TO ( PUBYEAR , 2012 ) ) AND ( LIMIT-TO ( DOCTYPE , "re" ) )

#7

"TITLE-ABS-KEY ( pharyngitis OR sore AND throat OR bacterial AND tonsillitis OR fusobacterium OR staphylococcus ) AND PUBYEAR > 2011 AND ( LIMIT-TO ( LANGUAGE, ""English"" ) ) AND ( LIMIT-TO ( DOCTYPE , ""re"" ) )

**COCHRANE LIBRARY**

#1

Recurrent Streptococcal pharyngitis tonsillitis treatment in children

#2

Streptococcal pharyngitis treatment in children with penicillin allergy

#3

amoxicillin AND (dose OR interval OR every 12 hours OR every 8 hours) AND streptococcal tonsillopharyngitis AND children

#4

pharyngitis and amoxicillin and (children or pediatrics) and (intravenous or intramuscular)

#5

**Search strings for clinical studies**

**PUBMED**

#1

(((((("child"[MeSH Terms] OR "child"[All Fields] OR "children"[All Fields] OR "child s"[All Fields] OR "children s"[All Fields] OR "childrens"[All Fields] OR "childs"[All Fields] OR ("child"[MeSH Terms] OR "child"[All Fields] OR "children"[All Fields] OR "child s"[All Fields] OR "children s"[All Fields] OR "childrens"[All Fields] OR "childs"[All Fields]) OR ("paediatrics"[All Fields] OR "pediatrics"[MeSH Terms] OR "pediatrics"[All Fields] OR "paediatric"[All Fields] OR "pediatric"[All Fields]) OR ("paediatrics"[All Fields] OR "pediatrics"[MeSH Terms] OR "pediatrics"[All Fields] OR "paediatric"[All Fields] OR "pediatric"[All Fields]) OR ("paediatrics"[All Fields] OR "pediatrics"[MeSH Terms] OR "pediatrics"[All Fields] OR "paediatric"[All Fields] OR "pediatric"[All Fields])) AND ("streptococcus pyogenes"[MeSH Terms] OR ("streptococcus"[All Fields] AND "pyogenes"[All Fields]) OR "streptococcus pyogenes"[All Fields]) AND ("palatine tonsil"[MeSH Terms] OR ("palatine"[All Fields] AND "tonsil"[All Fields]) OR "palatine tonsil"[All Fields] OR "tonsil"[All Fields] OR "tonsils"[All Fields] OR "tonsilitis"[All Fields] OR "tonsillitis"[MeSH Terms] OR "tonsillitis"[All Fields] OR "tonsillitides"[All Fields] OR "tonsills"[All Fields])) OR ("tonsillopharyngeal"[All Fields] OR "tonsillopharyngitis"[All Fields])) AND "dose"[All Fields]) OR ("dosage"[All Fields] OR "dosages"[All Fields])) AND ("amoxicillin"[MeSH Terms] OR "amoxicillin"[All Fields] OR "amoxicilline"[All Fields] OR "amoxicillins"[All Fields]) AND (("clinical trial"[Publication Type] OR "randomized controlled trial"[Publication Type]) AND 2012/01/01:2022/12/31[Date - Publication])) AND ((clinicaltrial[Filter] OR randomizedcontrolledtrial[Filter]) AND (2020:2022[pdat]))

#2

(((("streptococcus"[MeSH Terms] OR "streptococcus"[All Fields]) AND ("pyogene"[All Fields] OR "pyogenes"[All Fields])) OR ("streptococcus"[MeSH Terms] OR "streptococcus"[All Fields] OR "streptococcal"[All Fields]) OR "gabhs"[All Fields]) AND (("palatine tonsil"[MeSH Terms] OR ("palatine"[All Fields] AND "tonsil"[All Fields]) OR "palatine tonsil"[All Fields] OR "tonsil"[All Fields] OR "tonsils"[All Fields] OR "tonsilitis"[All Fields] OR "tonsillitis"[MeSH Terms] OR "tonsillitis"[All Fields] OR "tonsillitides"[All Fields] OR "tonsills"[All Fields] OR "pharyngit*"[All Fields] OR "nasopharyngit*"[All Fields] OR "rhinopharyngit*"[All Fields] OR "tonsillopharyngit*"[All Fields] OR "sore"[All Fields]) AND ("pharynx"[MeSH Terms] OR "pharynx"[All Fields] OR "throat"[All Fields] OR "throats"[All Fields])) AND ("amoxicillin"[MeSH Terms] OR "amoxicillin"[All Fields] OR "amoxicilline"[All Fields] OR "amoxicillins"[All Fields] OR ("beta lactams"[MeSH Terms] OR "beta lactams"[All Fields] OR ("beta"[All Fields] AND "lactam"[All Fields]) OR "beta lactam"[All Fields]) OR ("amoxicillin"[MeSH Terms] OR "amoxicillin"[All Fields] OR "amoxycillin"[All Fields] OR "amoxycilline"[All Fields]) OR ("ampicillin"[MeSH Terms] OR "ampicillin"[All Fields] OR "ampicilline"[All Fields] OR "ampicillins"[All Fields]) OR ("betalactam"[All Fields] OR "betalactams"[All Fields])) AND "child*"[All Fields]) AND ((clinicaltrial[Filter] OR randomizedcontrolledtrial[Filter]) AND (2012:2020[pdat]))

#3

(("streptococcus pyogenes"[MeSH Terms] OR ("streptococcus"[All Fields] AND "pyogenes"[All Fields]) OR "streptococcus pyogenes"[All Fields]) AND ("palatine tonsil"[MeSH Terms] OR ("palatine"[All Fields] AND "tonsil"[All Fields]) OR "palatine tonsil"[All Fields] OR "tonsil"[All Fields] OR "tonsils"[All Fields] OR "tonsilitis"[All Fields] OR "tonsillitis"[MeSH Terms] OR "tonsillitis"[All Fields] OR "tonsillitides"[All Fields] OR "tonsills"[All Fields]) AND ("dose"[All Fields] OR ("dosage"[All Fields] OR "dosages"[All Fields]) OR "dosis"[All Fields]) AND ("anti bacterial agents"[Pharmacological Action] OR "anti bacterial agents"[MeSH Terms] OR ("anti bacterial"[All Fields] AND "agents"[All Fields]) OR "anti bacterial agents"[All Fields] OR ("anti"[All Fields] AND "bacterial"[All Fields] AND "agents"[All Fields]) OR "anti bacterial agents"[All Fields] OR ("amoxicillin"[MeSH Terms] OR "amoxicillin"[All Fields] OR "amoxicilline"[All Fields] OR "amoxicillins"[All Fields]))) AND ((clinicaltrial[Filter] OR randomizedcontrolledtrial[Filter]) AND (2012:2020[pdat]))

#3

("streptococc*"[All Fields] AND ("pharyngitis"[MeSH Terms] OR "pharyngitis"[All Fields] OR ("sore"[All Fields] AND "throat"[All Fields]) OR "sore throat"[All Fields]) AND ("amoxicillin"[MeSH Terms] OR "amoxicillin"[All Fields] OR "amoxicilline"[All Fields] OR "amoxicillins"[All Fields])) AND (clinicaltrial[Filter] OR meta-analysis[Filter] OR randomizedcontrolledtrial[Filter] OR systematicreview[Filter])

#4

(("pharyngitis"[MeSH Terms] OR "pharyngitis"[All Fields] OR "pharyngitides"[All Fields] OR ("palatine tonsil"[MeSH Terms] OR ("palatine"[All Fields] AND "tonsil"[All Fields]) OR "palatine tonsil"[All Fields] OR "tonsil"[All Fields] OR "tonsils"[All Fields] OR "tonsilitis"[All Fields] OR "tonsillitis"[MeSH Terms] OR "tonsillitis"[All Fields] OR "tonsillitides"[All Fields] OR "tonsills"[All Fields]) OR ("pharyngitis"[MeSH Terms] OR "pharyngitis"[All Fields] OR ("sore"[All Fields] AND "throat"[All Fields]) OR "sore throat"[All Fields])) AND ("amoxicillin"[MeSH Terms] OR "amoxicillin"[All Fields] OR "amoxicilline"[All Fields] OR "amoxicillins"[All Fields] OR ("cephalosporine"[All Fields] OR "cephalosporines"[All Fields] OR "cephalosporins"[MeSH Terms] OR "cephalosporins"[All Fields] OR "cephalosporin"[All Fields]) OR ("macrolid"[All Fields] OR "macrolides"[MeSH Terms] OR "macrolides"[All Fields] OR "macrolide"[All Fields] OR "macrolids"[All Fields])) AND ("child"[MeSH Terms] OR "child"[All Fields] OR "children"[All Fields] OR "child s"[All Fields] OR "children s"[All Fields] OR "childrens"[All Fields] OR "childs"[All Fields] OR ("paediatrics"[All Fields] OR "pediatrics"[MeSH Terms] OR "pediatrics"[All Fields] OR "paediatric"[All Fields] OR "pediatric"[All Fields])) AND "intramuscular"[All Fields]) AND (y_10[Filter])

# 5

((("child"[MeSH Terms] OR "child"[All Fields] OR "children"[All Fields] OR "child s"[All Fields] OR "children s"[All Fields] OR "childrens"[All Fields] OR "childs"[All Fields]) AND ("pharyngitis"[MeSH Terms] OR "pharyngitis"[All Fields] OR "pharyngitides"[All Fields])) OR (("palatine tonsil"[MeSH Terms] OR ("palatine"[All Fields] AND "tonsil"[All Fields]) OR "palatine tonsil"[All Fields] OR "tonsil"[All Fields] OR "tonsils"[All Fields] OR "tonsilitis"[All Fields] OR "tonsillitis"[MeSH Terms] OR "tonsillitis"[All Fields] OR "tonsillitides"[All Fields] OR "tonsills"[All Fields]) AND ("streptococcus pyogenes"[MeSH Terms] OR ("streptococcus"[All Fields] AND "pyogenes"[All Fields]) OR "streptococcus pyogenes"[All Fields]) AND ("benzylpenicillins"[All Fields] OR "penicillin g"[MeSH Terms] OR "penicillin g"[All Fields] OR "benzylpenicillin"[All Fields] OR "penicilline"[All Fields] OR "penicillines"[All Fields] OR "penicillins"[MeSH Terms] OR "penicillins"[All Fields] OR "penicillin"[All Fields]) AND ("allergie"[All Fields] OR "hypersensitivity"[MeSH Terms] OR "hypersensitivity"[All Fields] OR "allergies"[All Fields] OR "allergy"[All Fields] OR "allergy and immunology"[MeSH Terms] OR ("allergy"[All Fields] AND "immunology"[All Fields]) OR "allergy and immunology"[All Fields]) AND ("recurrance"[All Fields] OR "recurrence"[MeSH Terms] OR "recurrence"[All Fields] OR "recurrences"[All Fields] OR "recurrencies"[All Fields] OR "recurrency"[All Fields] OR "recurrent"[All Fields] OR "recurrently"[All Fields] OR "recurrents"[All Fields]) AND ("rheumatic fever"[MeSH Terms] OR ("rheumatic"[All Fields] AND "fever"[All Fields]) OR "rheumatic fever"[All Fields]) AND ("hospital s"[All Fields] OR "hospitalisation"[All Fields] OR "hospitalization"[MeSH Terms] OR "hospitalization"[All Fields] OR "hospitalising"[All Fields] OR "hospitality"[All Fields] OR "hospitalisations"[All Fields] OR "hospitalised"[All Fields] OR "hospitalizations"[All Fields] OR "hospitalized"[All Fields] OR "hospitalize"[All Fields] OR "hospitalizing"[All Fields] OR "hospitals"[MeSH Terms] OR "hospitals"[All Fields] OR "hospital"[All Fields]) AND ("suppurate"[All Fields] OR "suppurated"[All Fields] OR "suppurating"[All Fields] OR "suppuration"[MeSH Terms] OR "suppuration"[All Fields] OR "suppurations"[All Fields] OR "suppurative"[All Fields]) AND ("complicances"[All Fields] OR "complicate"[All Fields] OR "complicated"[All Fields] OR "complicates"[All Fields] OR "complicating"[All Fields] OR "complication"[All Fields] OR "complication s"[All Fields] OR "complications"[MeSH Subheading] OR "complications"[All Fields])))

# 6

(Tonsillitis OR pharyngitis) AND (Streptococcus pyogenes) AND (Recurrence) AND (Penicillin V OR Amoxicillin-Potassium Clavulanate Combination OR macrolide OR cephalosporins OR clindamycin OR rifampicin OR Trimethoprim, Sulfamethoxazole Drug Combination)

**EMBASE**

#1

('pharyngitis'/exp OR pharyngitis) AND ('amoxicillin'/exp OR amoxicillin) AND ('children'/exp OR children OR 'pediatrics'/exp OR pediatrics OR 'paediatrics'/exp OR paediatrics) AND ([cochrane review]/lim OR [systematic review]/lim OR [meta analysis]/lim OR [controlled clinical trial]/lim OR [randomized controlled trial]/lim) AND [2012-2022]/py

#2

('streptococcal tonsillopharyngitis' OR (streptococcal AND ('tonsillopharyngitis'/exp OR tonsillopharyngitis))) AND ('antibiotic therapy'/exp OR 'antibiotic therapy' OR (('antibiotic'/exp OR antibiotic) AND ('therapy'/exp OR therapy))) AND ('child'/exp OR child)#1 AND (2020:py OR 2021:py OR 2022:py) AND ('clinical article'/de OR 'clinical trial'/de OR 'cohort analysis'/de OR 'comparative study'/de OR 'controlled clinical trial'/de OR 'controlled study'/de OR 'cross sectional study'/de OR 'double blind procedure'/de OR 'drug comparison'/de OR 'major clinical study'/de OR 'multicenter study'/de OR 'observational study'/de OR 'open study'/de OR 'prospective study'/de OR 'quantitative study'/de OR 'randomized controlled trial'/de OR 'randomized controlled trial topic'/de OR 'retrospective study'/de OR 'single blind procedure'/de)

#3

('streptococcal pharyngitis'/exp OR 'streptococcal pharyngitis') AND ('amoxicillin'/exp OR 'amoxicillin' OR 'penicillin derivative'/exp OR 'penicillin derivative') AND ('dose'/exp OR 'dose') AND ('child'/exp OR 'child' OR 'pediatrics'/exp OR 'pediatrics') AND (2020:py OR 2021:py OR 2022:py) AND ('clinical article'/de OR 'clinical trial'/de OR 'clinical trial topic'/de OR 'comparative study'/de OR 'controlled clinical trial'/de OR 'controlled study'/de OR 'double blind procedure'/de OR 'drug comparison'/de OR 'drug dosage form comparison'/de OR 'major clinical study'/de OR 'multicenter study'/de OR 'open study'/de OR 'prospective study'/de OR 'randomized controlled trial'/de OR 'randomized controlled trial topic'/de OR 'retrospective study'/de OR 'single blind procedure'/de

#3

('pharyngitis'/exp OR pharyngitis) AND ('amoxicillin'/exp OR amoxicillin) AND ('children'/exp OR children OR 'pediatrics'/exp OR pediatrics OR 'paediatrics'/exp OR paediatrics) AND (2012:py OR 2013:py OR 2014:py OR 2015:py OR 2016:py OR 2017:py OR 2018:py OR 2019:py OR 2020:py OR 2021:py OR 2022:py) AND 'randomized controlled trial'/de

#4

('pharyngitis'/exp OR pharyngitis) AND intramuscular AND ('children'/exp OR children OR 'pediatrics'/exp OR pediatrics) AND [2012-2022]/py

#5

('child'/exp OR 'streptococcal pharyngitis'/exp) AND 'penicillin allergy'/exp AND ('macrolide'/exp OR 'cephalosporin'/exp) AND 'amoxicillin'/exp AND ('relapse'/exp OR 'rheumatic fever'/exp OR 'retropharyngeal abscess'/exp OR 'hospitalization'/exp) AND ('systematic review'/exp OR 'meta analysis'/exp)

#6

('tonsillitis'/exp OR tonsillitis OR 'pharyngitis'/exp OR pharyngitis) AND 'recurrent disease' AND ('streptococcus'/exp OR streptococcus) AND pyogenes AND [2012-2022]/py AND [english]/lim

**SCOPUS**

#1 ( streptococcal AND pharyngitis AND amoxicillin AND dosage ) AND ( LIMIT-TO ( PUBYEAR , 2021 ) ) AND ( LIMIT-TO ( LANGUAGE , "English" ) )

#2 (( streptococcus OR streptoccocal OR gabhs ) AND ( pharingitis OR tonsillitis ) AND amoxicillin ) AND ( LIMIT-TO ( PUBYEAR , 2022 ) OR LIMIT-TO ( PUBYEAR , 2021 ) OR LIMIT-TO ( PUBYEAR , 2020 ) ) AND ( LIMIT-TO ( LANGUAGE , "English" ) )

#3 ( TITLE-ABS-KEY ( pharyng* )  AND  TITLE-ABS-KEY ( streptococ*  AND pyogenes )  AND  TITLE-ABS-KEY ( antibiotic  AND therapy )

#4 TITLE-ABS-KEY ( pharyngitis AND streptococcus AND pyogenes AND recurrence ) AND PUBYEAR > 2011

**COCHRANE LIBRARY
(trials registry)**

#1 sore throat amoxicillin

5 Trials matching sore throat amoxicillin in All Text - with Publication Year from 2020 to 2022, in Trials (Word variations have been searched)

#2 streptococcal pharyngitis amoxicillin

2 Trials matching streptococcal pharyngitis amoxicillin in All Text - with Publication Year from 2020 to 2022, in Trials (Word variations have been searched)

#3 streptococcal tonsillitis amocixillin

#4 Streptococcal pharyngitis treatment in children with penicillin allergy

**Figure A2.1** **Flow diagram of literature search and data extraction for systematic reviews**

## Identification

SR excluded

(n = 80 )

SR included (n =5 )

Records screened after exclusion of duplicates

(n = 586)

## Screening

**Additional records identified by manual screening**
(n = 5 )

**Records identified from databases:**

PUBMED N= 1089

EMBASE N = 358

SCOPUS N= 389

COCHRANE LIBRARY N = 56

Systematic review (SR) assessed for eligibility

(n = 85 )

## Included

Records excluded by title and abstract

(n = 427)

1 esiti non pertinenti

1 intervento non pertinente

)

## Eligibility

Non- pertinent excluded studies

(n = 77)

1 esiti non pertinenti

1 intervento non pertinente

)

Full text articles screened

(n =159)

**Figure A2.2. Flow diagram of literature search and data extraction for clinical studies**

Full text articles screened
(n =61)

Non- pertinent excluded studies

(n=14)

Records excluded by title and abstract

(n=71)

Clinical studies excluded

(n = 43)

Included clinical studies

(n = 4)

**Records identified from databases:**

PUBMED n = 531

EMBASE n = 220

COCHRANE n =7

SCOPUS n=81

**Additional records identified by manual screening** (n = 2)

Records screened after exclusion of duplicates
(n =132)

## Identification

## Included

## Eligibility

## Screening

Full-text clinical studies assessed for eligibility
(n = 47)
