# Supplementary material for: Treatment of acute pharyngitis in children: an Italian intersociety consensus (SIPPS-SIP-SITIP-FIMP-SIAIP-SIMRI-FIMMG)
Source: Ital J Pediatr. 2024 Nov 6;50:235. doi: 10.1186/s13052-024-01789-5 (PMC11539554; doi:10.1186/s13052-024-01789-5)
Supplement: Supplementary file 3 — Additional file 3: Quality assessment of systematic reviews. The quality of systematin reviews has been assessed through the AMSTAR 2 checklist. The results are provided in the Additional file 3. [file 13052_2024_1789_MOESM3_ESM.doc]

**Additional File 3 – Quality assessment of systematic reviews**

| **AMSTAR 2** | **Altamimi et al. 2012** | **Munck et al. 2018** | **Spinks et al.**  **2021** | **Spurling et al. 2017** | **Van Driel et al. 2021** |
| --- | --- | --- | --- | --- | --- |
| 1. Did the research questions and inclusion criteria for the review include the PICO components? (Yes/No) | Yes | Yes | Yes | Yes | Yes |
| 2. Did the RS report contain an explicit statement that the review methods were established before the review, and did the report justify any significant deviations from the protocol? (Yes/Partial/No) | Yes | No | Yes | Yes | Yes |
| 3. Did the review authors justify their selection of the study designs included in the review? (Yes/No) | Yes | Yes | Yes | Yes | Yes |
| 4. Did the review authors use a comprehensive bibliographic search strategy? (Yes/Partial/No) | Yes | Partial | Partial | Yes | Yes |
| 5. Did the review authors perform study selection in duplicate? (Yes/No) | Yes | Yes | Yes | Yes | Yes |
| 6. Did the review authors perform data extraction in duplicate? (Yes/No) | Yes | Yes | Yes | Yes | Yes |
| 7. Did the review authors provide a list of excluded studies and justify the exclusions? (Yes/Partial/No) | Yes | No | Yes | Yes | Yes |
| 8. Did the review authors describe the included studies in adequate detail? (Yes/Partial/No) | Yes | Yes | Yes | Yes | Yes |
| 9. Did the review authors use a satisfactory technique to assess the risk of bias (RoB) in individual studies included in the RS? (Yes/Partial/No/Includes only NRSI-RCT) | Yes | Yes | Yes | Yes | Yes |
| 10. Did the review authors report the sources of funding for the studies included in the review? (Yes/No) | No | No | No | Yes | Yes |
| 11. If a meta-analysis was performed, did the review authors use appropriate methods for the statistical combination of results? (Yes/No/No meta-analysis conducted) | Yes | No meta-analysis conducted | Yes | Yes | Yes |
| 12. If a meta-analysis was performed, did the review authors assess the potential impact of RoB in individual studies on the results of the meta-analysis or other evidence syntheses? (Yes/No/No meta-analysis conducted) | Yes | No meta-analysis conducted | Yes | Yes | Yes |
| 13. Did the review authors consider the RoB in individual studies when interpreting/discussing the results of the review? (Yes/No) | Yes | Yes | Yes | Yes | Yes |
| 14. Did the review authors provide a satisfactory explanation and discussion of any observed heterogeneity in the results of the review? (Yes/No) | Yes | Yes | Yes | Yes | Yes |
| 15. If a quantitative synthesis was performed, did the review authors conduct an adequate investigation of publication bias (small study bias) and discuss its likely impact on the results of the review? (Yes/No/No meta-analysis conducted) | Yes | No meta-analysis conducted | Yes | Yes | Yes |
| 16. Did the review authors report potential sources of conflicts of interest, including any funding received for conducting the review? (Yes/No) | Yes | Yes | Yes | Yes | Yes |
| **Overall Assessment** | High Methodological Quality | Low Methodological Quality | High/Moderate Methodological Quality | High Methodological Quality | High Methodological Quality |

**CRITICAL DOMAINS AMSTAR 2**

- Protocol registered before the review (point 2)
- Adequacy of the bibliographic search (point 4)
- Justification for exclusion of individual studies (point 7)
- Risk of bias arising from individual studies included in the review (point 9)
- Appropriateness of meta-analytic methods (point 11)
- Consideration of the risk of bias in the interpretation of review results (point 13)
- Evaluation of the presence and probable impact of publication bias (point 15)

Excluded RS with Reason

| **EXCLUDED RS** | **Reason for Exclusion** |
| --- | --- |
| Bennet et al. 2021 | Narrative review |
| Holm et al. 2020 | Critically low methodological quality |
| Kalra et al. 2016 | Narrative review |
| Murray et al. 2012 | Narrative review |
| Mustafa et al. 2020 | Narrative review. |
| Oliver et al. 2018 | Critically low methodological quality |
| Radetsky et al. 2017 | Narrative review |
| Raghav et al. 2022 | Narrative review |
| Robinson et al. 2020 | Narrative review |
| Robinson et al. 2021 | Narrative review |
| Rojas-Ramírez et al. 2017 | Narrative review |
| Spurling et al. 2017 | Not pertinent. It evaluates IR for pharyngotonsillitis, includes only one adult study (De la Poza Abad 2016) |
| Stelter et al. 2014 | Narrative review |
| Van Brusselen et al. 2014 | Narrative review |
| Vazquez et al. 2017 | Narrative review |

|  | Altamimi S et al. 2012 | Not pertinent to the question |  |
| --- | --- | --- | --- |
|  | Anjos et al. 2014 | Review article. Outdated references (>10 years) |  |
|  | Brook I. 2017 | Narrative review |  |
|  | Hoare K.J. et al. | Not pertinent to the question |  |
|  | Hoban et al. 2019 | Critically low methodological quality. Funded by a pharmaceutical company, of which the 2 authors are employees |  |
|  | Mustafa et al. 2020 | Narrative review. |  |
|  | Norton L et al. 2021 | Narrative review |  |
|  | Oliver et al. 2018 | Not pertinent to the question. Low methodological quality |  |
|  | Spinks et al. 2021 | Not pertinent. It did not discuss alternative therapies to penicillin. Among the exclusion criteria of the studies there are children with a history of penicillin allergy. |  |
|  | Stelter K. Et al. 2014 | Narrative review |  |
|  | van Driel et al. 2013 | Update available |  |
|  | van Driel et al. 2016 | Update available |  |
|  | van Driel et al. 2021 | Not pertinent. It included studies reporting the type of adverse reaction in case of penicillin allergy without addressing the issue of alternative therapies. |  |
|  | Altamimi S et al. 2012 | Not pertinent. Different antibiotic therapies compared to penicillin in patients with recurrent pharyngotonsillitis |  |
|  | Brook I. 2017 | Narrative review |  |
|  | Burton et al. 2014 | Not pertinent. It does not include comparison studies between different antibiotic therapies |  |
|  | Ng et al. 2015 | Not pertinent. All selected studies excluded (no trial met inclusion criteria) |  |
|  | van Driel et al. 2021 | Not pertinent. Different antibiotic therapies compared to penicillin in patients with recurrent pharyngotonsillitis |  |
|  | Albrecht et al. 2018 | Not pertinent. No comparison study between two different dosages of amoxicillin. |  |
|  | Altamimi et al. 2012 | Not pertinent. No comparison study between two different dosages of amoxicillin. |  |
|  | Bateman et al. 2022 | Not pertinent. It evaluates the effectiveness of an antibiotic therapy in preventing post-streptococcal glomerulonephritis. No comparison study between two different dosages of amoxicillin. |  |
|  | Brook et al. 2017 | Not pertinent. No comparison study between two different dosages of amoxicillin. |  |
|  | Cirilli et al. 2013 | Not pertinent. No comparison study between two different dosages of amoxicillin. |  |
|  | Couic-Marinier et al. 2017 | Not pertinent. No comparison study between two different dosages of amoxicillin. |  |
|  | Dawson-Hahn et al 2017 | Not pertinent. No comparison study between two different dosages of amoxicillin. |  |
|  | Deng et al. 2013 | Not pertinent. No comparison study between two different dosages of amoxicillin. |  |
|  | Garazzino et al. 2013 | Not pertinent. No comparison study between two different dosages of amoxicillin. |  |
|  | Gualtieri et al. 2021 | Not pertinent. It evaluates streptococcal perianitis but not pharyngotonsillitis. |  |
|  | Hoban et al. 2019 | Not pertinent. It evaluates clarithromycin. |  |
|  | Leung et al. 2018 | Not pertinent. No comparison study between two different dosages of amoxicillin. |  |
|  | Llerena Santa Cruz et al. 2011 | Not pertinent. Comparison between amoxicillin administered once a day vs twice a day. |  |
|  | Oliver et al. 2018 | Not pertinent. It evaluates the necessity of antibiotic therapy in different settings. |  |
|  | Rojas-Ramírez et al. 2017 | Not pertinent. No comparison study between two different dosages of amoxicillin. |  |
|  | Seifert et al. 2019 | Not pertinent. It evaluates whether treatment with EPs 7630 reduces paracetamol consumption in children with acute pharyngotonsillitis or acute bronchitis. |  |
|  | Van Brusselen et al. 2014 | Not pertinent. No comparison study between two different dosages of amoxicillin. |  |
|  | van Driel et al. 2021 | Not pertinent. No comparison study between two different dosages of amoxicillin. |  |
|  | Wilcox et al. 2019 | Not pertinent. Evaluates the effectiveness of the probiotic Streptococcus salivarius K12 for the treatment/prevention of pharyngitis. |  |
|  | Brook et al. 2013 | Not pertinent. No comparison between intramuscular and oral route. |  |
|  | Chan et al. 2015 | Not pertinent. Practical recommendations. No comparison between intramuscular and oral route. |  |
|  | Cohen et al. 2017 | Not pertinent. No comparison between intramuscular and oral route. |  |
|  | Espadas et al. 2018 | Not pertinent. No comparison between intramuscular and oral route. |  |
|  | Homme et al. 2019 | Not pertinent. No comparison between intramuscular and oral route. |  |
|  | Kalra et al. 2016 | Not pertinent. No comparison between intramuscular and oral route. |  |
|  | Robinson et al. 2021 | Narrative review |  |
|  | Shulman et al. 2012 | Not pertinent. Clinical practice guidelines. No comparison between intramuscular and oral route. |  |
|  | Zacharioudaki et al. 2017 | Not pertinent. It evaluates eradication of GABHS in carriers. |  |
|  | Zeng et al. 2014 | Not pertinent. No comparison between intramuscular and oral route. |  |
|  | Bai Y et al. 2020 | Not pertinent. It evaluates the effectiveness of Pudilan Xiaoyan Oral Liquid (PDL) |  |
|  | Bian LL et al. 2017 | Not pertinent. It evaluates the effectiveness of Pudilan Xiaoyan Oral Liquid (PDL) |  |
|  | Bird et al. 2014 | Narrative review |  |
|  | Burton MJ et al. 2014 | Not pertinent. Only surgery |  |
|  | Cavalcanti et al. 2019 | Original article. Not SR |  |
|  | Centor et al. 2022 | Narrative review |  |
|  | De Cassan et al. 2020 | Not pertinent. It evaluates non-corticosteroid anti-inflammatory therapy |  |
|  | Eilbert et al. 2013 | Narrative review |  |
|  | Georgalas CC et al. 2014 | Not pertinent. Only surgical techniques |  |
|  | Gunnarsson RK et al. 2020 | Not pertinent. It investigates the prevalence of Streptococcus C infection/colonization |  |
|  | Hoare KJ et al. 2016 | Not pertinent. SR on guidelines application modes for pharyngotonsillitis in New Zealand |  |
|  | Holm et al. 2020 | Not pertinent. Critically low methodological quality |  |
|  | Hu XY et al 2017 | Not pertinent. It evaluates the effectiveness of Andrographis paniculata |  |
|  | Kamfose et al. 2020 | Not pertinent. It evaluates injectable therapy |  |
|  | Kannan et al. 2015 | Narrative review |  |
|  | Kizhner, V et al. 2013 | Narrative review |  |
|  | Kostić et al. 2020 | Narrative review |  |
|  | Klug et al. 2016 | Not pertinent |  |
|  | Kuppali et al. 2012 | Narrative review |  |
|  | Lee et al. 2020 | Narrative review |  |
|  | Malmberg et al. 2021 | Not pertinent. Epidemiological study |  |
|  | Marais et al. 2019 | Narrative review |  |
|  | Marchello et al. 2016 | Not pertinent. Epidemiological study |  |
|  | Morad A et al. 2017 | Not pertinent. Only surgical treatment discussed. |  |
|  | Munck et al. 2018 | Not pertinent |  |
|  | Norton et al. 2021 | Not pertinent |  |
|  | Pallon et al. 2018 | Observational study |  |
|  | Patel et al. 2020 | Narrative review |  |
|  | Patini et al. 2020 | Not pertinent: adults, purpose to assess antibiotic resistance rates |  |
|  | Rosanova MT et al. 2016 | Not pertinent. Only comparison between sulfamethoxazole and wait and see strategy |  |
|  | Sayad et al. 2021 | ​​ |  |
|  | |  | |
| Lee et al. 2020 | | Narrative review | |
| Malmberg et al. 2021 | | Not pertinent. Epidemiological study | |
| Marais et al. 2019 | | Narrative review | |
| Marchello et al. 2016 | | Not pertinent Epidemiological study | |
| Morad A et al. 2017 | | Not pertinent. Only surgical treatment discussed. | |
| Munck et al. 2018 | | Not pertinent | |
| Norton et al. 2021 | | Not pertinent | |
| Pallon et al. 2018 | | Not pertinent | |
| Patel et al. 2020 | | Narrative review | |
| Patini et al. 2020 | | Not pertinent: adults, aims to evaluate antibiotic resistance rates | |
| Rosanova MT et al. 2016 | | Not pertinent. Only comparison between sulfamethoxazole and wait and see strategy | |
| Sayad et al. 2021 | | Critically low methodological quality | |
| Wilkinson et al 2012 | | Narrative review | |
| Wright et al. 2012 | | Narrative review | |
| Zeng et al. 2014 | | Not pertinent | |
