# Supplementary material for: Treatment of acute pharyngitis in children: an Italian intersociety consensus (SIPPS-SIP-SITIP-FIMP-SIAIP-SIMRI-FIMMG)
Source: Ital J Pediatr. 2024 Nov 6;50:235. doi: 10.1186/s13052-024-01789-5 (PMC11539554; doi:10.1186/s13052-024-01789-5)
Supplement: Supplementary file 4 — Additional file 4: Quality of evidence. For each question the quality of evidence was assessed according to the GRADE methodology and results are provided as tables in the Additional file 4 (Tables A4.1- A4.7). [file 13052_2024_1789_MOESM4_ESM.docx]

**Additional File 4– Quality of evidence**

**Question 1.**

**Should Group A β-haemolytic streptococcus (GABHS) pharyngotonsillitis be treated with antibiotics?**

**Question:** [antibiotic therapy] compared to [no therapy or symptomatic therapy] for [streptococcal pharyngotonsillitis in pediatric patients]

**Setting:** Outpatient

**Table A4.1. Quality of evidence for Question 1**

| **Certainty assessment** | | | | | | | **№ of patients** | | **Effect** | | **Certainity** | **Importance** |
| --- | --- | --- | --- | --- | --- | --- | --- | --- | --- | --- | --- | --- |
| **Number ot study** | **Study design** | **Risk of Bias** | **Inconsistency** | **Indirecteness** | **Imprecision** | **Other considerations** | **Antibiotic therapy** | **No therapy** | **RR (95% CI)** | **Absolute (95% CI)** |  |  |
| **Symptoms on day 3 (follow-up: average 3 days; assessed with: % of patients with symptoms)** | | | | | | | | | | | | |
| 11^1^ | Randomized study | serious^a^ | not important | serious^b^ | not important | strong association | 471/1073 (43.9%) | 544/766 (71.0%) | **RR 0.58** (0.48 to 0.71) | **298 less per 1.000** (from 369 less to 206 less) | ⨁⨁⨁◯ Moderate | IMPORTANT |
| **Symptoms on day 7 – GABHS positive swab (follow-up: range 6-8 days; assessed with: % of patients with symptoms)** | | | | | | | | | | | | |
| 7^2^ | Randomized study | not important | not important | serious^b^ | not important | strong association | 22/650 (3.4%) | 57/467 (12.2%) | **RR 0.29** (0.12 to 0.70) | **87 less per 1.000** (from 107 less to 37 less) | ⨁⨁⨁⨁ HIgh | IMPORTANT |
| **Complications: Acute Rheumatic Fever (ARF) within 2 months (follow-up: average 2 months; assessed with: % of patients with ARF)** | | | | | | | | | | | | |
| 14^3^ | Randomized study | not important | not important | serious^b^ | not important | strong association | 22/4332 (0.5%) | 74/3843 (1.9%) | **RR 0.27** (0.14 to 0.50) | **14 less per 1.000** (from 17 less to 10 less) | ⨁⨁⨁⨁ HIgh | CRITICAL |
| **Complications: Peritonsillar abscess (follow-up: average 2 months; assessed as % of patients with abscess)** | | | | | | | | | | | | |
| 8^4^ | Randomized study | serious^a^ | not important | serious^b^ | not important | strong association | 2/1438 (0.1%) | 23/995 (2.3%) | **RR 0.15** (0.05 to 0.47) | **20 less per 1.000** (from 22 less to 12 less) | ⨁⨁⨁◯ Moderate | CRITICAL |

**CI:** Confidence interval; **RR:** Risk ratio

#### Explanations:

#### a. Data aggregated from patients with sore throat of various etiologies;

#### b. Data aggregated from studies on pediatric and adult patients

#### References

1. RS_Spinks,et al.2013 (Brink 1951, Brumfitt 1957, Chappel 1956, De,Meyere 1992, Fromnny 1953, El-Fromher 1991, Krober 1985, MacDonald 1951, Middleton1 988, Zwart 2000, , Zwart 2003. . 2013.

2. RS_Spinks,et al.2013 (Brink, 1951, Brumfitt1957, Fromgnelie 1996, De Meyere1992, Fromnny 1953, MacDonald 1951, 2003), Zwart. . 2013.

3. RS_Spinks,et al.2013 (Bennike, 1951, Brink, 1957, Brumfitt, 1954, Catanzaro, 1954, Chamovitz, 1956, Chappel, 1996, Fromgnelie, 1992, De,Meyere, Fromnny 1950 e Fromnny 1953, Pichichero 1987, 1961, Siegle, 1951, Wannamaker, 2000), Zwart 2013.

4. RS_Spinks,et al.2013 (Bennike, 1951, Fromgnelie 1996, De,Meyere 1992, Howe 1997, Landsman, 1951, Little 1997, Pichichero, 1987, Zwart 2000)

**Question 2.** **Should amoxicillin be considered the antibiotic of choice in the treatment of GABHS pharyngotonsillitis besides penicillin V?**

**Question:** [antibiotic therapy with antibiotics other than amoxicillin (amoxi-clav, macrolides, cephalosporins, etc)] compared to [amoxicillin] for [streptococcal pharyngotonsillitis in pediatric patients]

**Setting:** Outpatient

**Table A4.2. Quality of evidence for Question 2**

| **Certainty assessment** | | | | | | | **№ of patients** | | **Effect** | | **Certainity** | **Importance** |  |
| --- | --- | --- | --- | --- | --- | --- | --- | --- | --- | --- | --- | --- | --- |
| **Number ot study** | **Study design** | **Risk of Bias** | **Inconsistency** | **Indirecteness** | **Imprecision** | **Other considerations** | **Other Antibiotic therapy** | **amoxicillin** | **RR (95% CI)** | **Absolute (95% CI)** |  |  |  |
| **Amoxicillin-clavu**lanate **(3 d**ays**) vs. Amoxicillin (10 d**ays**)- Resolution of symptoms at the end of treatment (follow-up: 10 days; assessed with: % difference in patients)** | | | | | | | | | | | | |  |
| 1^1^ | Randomized study | serious^a^ | serious^b^ | not important | not important | none | 53/54 (98.1%) | 40/43 (93.0%) | **RR 1.06** (0.96 to 1.15) | **56 less per 1.000** (from 37 less to 140 less) | ⨁⨁◯◯ Low | CRITICAL |  |
| **Eradication of GABHS after 15 days (follow-up: 15 days; assessed with: % of patients with negative swab for GABHS)** | | | | | | | | | | | | |  |
| 1^1^ | Randomized study | serious^a^ | serious^b^ | not important | not important | none | 35/54 (64.8%) | 37/43 (86.0%) | **RR 0.73** (0.53 to 0.92) | **232 less per 1.000** (from 404 less to 69 less) | ⨁⨁◯◯ Low |  |  |
| **Cephalosporins vs. Penicillin V - Resolution of symptoms after treatment (ITT subgroup analysis of 855 pediatric patients) (follow-up: 2 to 15 days; assessed with: % of patients with persistent symptoms)** | | | | | | | | | | | | |  |
| 3^2^ | Randomized study | very serious^a,c^ | not important | not important | not important | Publication bias suspected^a^ |  |  | **OR 0.83** (0.40 to 1.73) | **1 less per 1.000** (from 2 less to 0 less) | ⨁◯◯◯ Very low | CRITICAL |  |
| **Risk of recurrence in 1386 patients (follow-up: 15 to 90 days; assessed as % of patients with recurrence)** | | | | | | | | | | | | |  |
| 4^2^ | Randomized study | serious^a,d^ | not important | serious^e^ | not important | none |  |  | **OR 0.55** (0.30 to 0.99) | **1 less per 1.000** (from 1 less to 0 less) | ⨁⨁◯◯ Low | IMPORTANT |  |
| **Risk of adverse events - ITT analysis on 1279 patients (follow-up: 15 days; assessed with: % of patients with adverse events)** | | | | | | | | | | | | |  |
| 3^2^ | Randomized study | serious^a^ | not important | serious^e^ | serious^f^ | none |  |  | **OR 0.94** (0.27 to 3.25) | **1 less per 1.000** (from 3 less to 0 less) | ⨁◯◯◯ Very low | IMPORTANT |  |
| **MACROLIDES vs. PENICILLIN V - Clinical efficacy (ITT analysis on 1728 patients) (follow-up: 2 to 20 days; assessed with: % of patients with symptom resolution post-treatment)** | | | | | | | | | | | | |  |
| 6^2^ | Randomized study | serious^a,f^ | not important | serious^e^ | serious^f^ | none |  |  | **OR 1.11** (0.92 to 1.35) | **1 less per 1.000** (from 1 less to 1 less) | ⨁◯◯◯ Very low | CRITICAL |  |
| **Risk of Adverse Events (ITT analysis on 1727 patients) (follow-up: 2 to 20 days; assessed with: % of patients with adverse events)** | | | | | | | | | | | | |  |
| 6^2^ | Randomized study | serious^a^ | not important | serious^e^ | not important | none |  |  | **OR 1.19** (0.82 to 1.73) | **1 less per 1.000** (from 2 less to 1 less) | ⨁⨁◯◯ Low | IMPORTANT |  |

#### CI: Confidence interval; OR: Odds ratio; RR: Risk ratio

#### Explanations:

#### a. Low methodological quality

#### b. Single study

#### c. Subgroup analysis

#### d. Very low methodological quality

#### e. Aggregated data from adult and pediatric patients

#### f. Wide 95% CI

#### References

1.al., Kuroki,et. al 2013.

2.VanDriel, RS 2021.

**Question 2. Should amoxicillin be considered the antibiotic of choice in the treatment of GABHS pharyngotonsillitis besides penicillin V?**

**Question:** [antibiotic therapy with antibiotics other than amoxicillin (amoxi-clav, macrolides, cephalosporins, etc)] compared to [amoxicillin] for [streptococcal pharyngotonsillitis in pediatric patients]

**Setting:** Outpatient

| **Certainty assessment** | | | | | | | **№ of patients** | | **Effect** | | **Certainity** | **Importance** |  |
| --- | --- | --- | --- | --- | --- | --- | --- | --- | --- | --- | --- | --- | --- |
| **Number ot study** | **Study design** | **Risk of Bias** | **Inconsistency** | **Indirecteness** | **Imprecision** | **Other considerations** | **Other Antibiotic therapy** | **amoxicillin** | **RR (95% CI)** | **Absolute (95% CI)** |  |  |  |
| **AZITHROMYCIN (10 mg/kg/day for 3 days) vs. AMOXICILLIN (30 mg/kg/day in 3 divided doses for 10 days) (follow-up: 14 to 30 days; assessed with: % of patients with symptom remission)** | | | | | | | | | | | | |  |
| 1^1^ | Randomized study | serious^e^ | serious^b^ | not important | not important | none | 80/83 (96.4%) | 71/78 (91.0%) | **RR 0.40** (0.11 to 1.50) | **546 less per 1.000** (from 810 less to 455 less) | ⨁⨁◯◯ Low | CRITICAL |  |
| **Bacterial eradication (follow-up: 14 to 30 days; assessed with: % of patients with a negative swab at the end of treatment)** | | | | | | | | | | | | |  |
| 1^1^ | Randomized study | serious^e^ | serious^b^ | not important | not important | none | 78/83 (94.0%) | 69/78 (88.5%) | **RR 0.52** (0.18 to 1.49) | **425 less per 1.000** (from 725 less to 433 less) | ⨁⨁◯◯ Low | IMPORTANT |  |
| **Adverse events (follow-up: 14 to 30 days; assessed with: % of patients with adverse events during treatment)** | | | | | | | | | | | | |  |
| 1^1^ | Randomized study | serious^e^ | serious^b^ | not important | not important | none | 2/83 (2.4%) | 9/79 (11.4%) | **RR 0.21** (0.05 to 0.95) | **90 less per 1.000** (from 108 less to 6 less) | ⨁⨁◯◯ Low | IMPORTANT |  |
| **Azithromycin (10 mg/kg/day for 3 days) vs. Penicillin v for 10 days - clinical efficacy on 1366 patients (follow-up: 1 to 10 days; assessed with: % of patients with symptom remission at the end of treatment)** | | | | | | | | | | | | |  |
| 6^2^ | Randomized study | serious^c^ | not important | serious^d^ | not important | none |  |  | **OR 1.05** (0.66 to 1.66) | **1 less per 1.000** (from 2 less to 1 less) | ⨁⨁◯◯ Low | CRITICAL |  |
| **Bacterial eradication (failure) on 1354 patients (follow-up: 1 to 10 days; assessed with: % of patients with a positive swab at the end of treatment)** | | | | | | | | | | | | |  |
| 6^2^ | Randomized study | serious^a^ | not important | serious^d^ | not important | none |  |  | **OR 3.25** (2.47 to 4.27) | **3 less per 1.000** (from 4 less to 2 less) | ⨁⨁◯◯ Low | IMPORTANT |  |
| **Adverse events in 1538 patients (assessed with: % of patients experiencing adverse events during treatment)** | | | | | | | | | | | | |  |
| 6^2^ | Randomized study | serious^a^ | not important | serious^d^ | not important | none |  |  | **OR 2.20** (1.49 to 3.24) | **2 less per 1.000** (from 3 less to 1 less) | ⨁⨁◯◯ Low | IMPORTANT |  |

#### CI: Confidence interval; OR: Odds ratio; RR: Risk ratio

#### Explanations:

#### a. Low methodological quality

#### b. Single study

#### c. Very low methodological quality

#### d. Aggregated data from adult and pediatric patients

#### e. Risk of bias due to lack of allocation concealment and blinding

#### References

1.al, Li,et. . 2019.

2.al, HIghmimi,et. . 2012.

**Question 2. Should amoxicillin be considered the antibiotic of choice in the treatment of GABHS pharyngotonsillitis besides penicillin V?**

**Question:** [antibiotic therapy with antibiotics other than amoxicillin (amoxi-clav, macrolides, cephalosporins, etc)] compared to [amoxicillin] for [streptococcal pharyngotonsillitis in pediatric patients]

**Setting:** Outpatient

| **Certainty assessment** | | | | | | | **№ of patients** | | **Effect** | | **Certainity** | **Importance** |
| --- | --- | --- | --- | --- | --- | --- | --- | --- | --- | --- | --- | --- |
| **Number ot study** | **Study design** | **Risk of Bias** | **Inconsistency** | **Indirecteness** | **Imprecision** | **Other considerations** | **Other antibiotic therapy** | **Amoxicillin** | **RR (95% CI)** | **Absolute (95% CI)** |  |  |
| **AZITHROMYCIN (20 mg/kg/die for 3 days) vs. PENICILLIN V for 10 days- 520 patients (follow up: 3 to 10 days; assessed by: % patients with clinical resolution)** | | | | | | | | | | | | |
| 2^1^ | Randomized study | serious^a,b^ | not important | not important | not important | none |  |  | **OR 0.80** (0.67 to 0.94) | **1 less per 1.000** (from 1 less to 1 less) | ⨁⨁⨁◯ Moderate | CRITICAL |
| **Bacterial eradication in 520 patients (follow-up: 1 to 10 days; assessed by: % of patients with a negative swab at the end of treatment)** | | | | | | | | | | | | |
| 2^1^ | Randomized study | serious^a^ | not important | not important | not important | none |  |  | **OR 0.29** (0.14 to 0.61) | **0 less per 1.000** (from 1 less to 0 less) | ⨁⨁⨁◯ Moderate | IMPORTANT |
| **Late clinical recurrence in 465 patients (follow-up: 2 weeks to 1 year; assessed with: % of patients with recurrence of GABHS pharyngotonsillitis)** | | | | | | | | | | | | |
| 2^1^ | Randomized study | serious^a,b^ | not important | not important | not important | none |  |  | **OR 0.95** (0.83 to 1.08) | **1 less per 1.000** (from 1 less to 1 less) | ⨁⨁⨁◯ Moderate | IMPORTANT |
| **Adverse events in 653 patients (assessed with: % of patients experiencing adverse events)** | | | | | | | | | | | | |
| 2^1^ | Randomized study | serious^a,b^ | not important | not important | not important | none |  |  | **OR 5.13** (2.76 to 9.54) | **5 less per 1.000** (from 10 less to 3 less) | ⨁⨁⨁◯ Moderate | IMPORTANT |

**CI:** Confidence interval; **OR:** Odds ratio; **RR:** Risk ratio

**Explanations:**

**- a. Low methodological quality**

**- b. Aggregated data from adult and pediatric patients**

#### References

1.1995, RS,HIghmimi,(Aujard, 2004), Kafetzis. . 2012.

**Question 3.** **Should the duration of antibiotic therapy for GABHS pharyngotonsillitis be shorter than 10 days?**

**Question:** [amoxicillin therapy lasting < 10 days] compared to [amoxicillin therapy for 10 days] for [streptococcal pharyngotonsillitis in pediatric patients] **Setting:** Outpatient

**Table A4.3 Quality of evidence for question 3**

| **Certainty assessment** | | | | | | | **№ of patients** | | | **Effect** | | **Certain** | | **Importance** | |  |
| --- | --- | --- | --- | --- | --- | --- | --- | --- | --- | --- | --- | --- | --- | --- | --- | --- |
| **N° of studies** | **Study design** | **Risk of bias** | **Risk of bias** | **Inconsistency** | **Imprecision** | **Other considerations** | **amoxicillin < 10 days** | **amoxicillin 10 days** | | **Relative (95% CI)** | **Absolute (95% CI)** |  |  |  |  |  |
| **Remission of symptoms (follow-up: 10 days; assessed with: % of patients with symptom remission at the end of treatment)** | | | | | | | | | | | | | | | |  |
| 1^1^ | Randomized study | serious^a^ | serious^a^ | not important | not important | none | 128/141 (90.8%) | 121/136 (89.0%) | | **RR 1.02** (0.94 to 1.10) | **18 less per 1.000** (from 53 less to 89 less) | ⨁⨁◯◯ Low | | | CRITICAL |  |
| **Risk of recurrence (follow-up: 30 days; assessed with: % of patients with positive swab recurrence)** | | | | | | | | | | | | | | | |  |
| 1^1^ | Randomized study | serious^a^ | serious^a^ | not important | not important | none | 11/141 (7.8%) | 6/136 (4.4%) | | **RR 1.77** (0.67 to 4.65) | **34 less per 1.000** (from 15 less to 161 less) | ⨁⨁◯◯ Low | | IMPORTANT | |  |
| **Safety (follow-up: 10 days; assessed with: % of patients with adverse events)** | | | | | | | | | | | | | | | |  |
| 1^1^ | Randomized study | serious^a^ | serious^a^ | not important | not important | none | 4/160 (2.5%) | 8/158 (5.1%) | | **RR 0.53** (0.16 to 1.53) | **24 less per 1.000** (from 43 less to 27 less) | ⨁⨁◯◯ Low | | IMPORTANT | |  |
| **Adherence (follow-up: 10 days; assessed by: % of patients non-adherent to the treatment)** | | | | | | | | | | | | | | | |  |
| 1^1^ | Randomized study | serious^a^ | serious^a^ | not important | not important | none | 20/159 (12.6%) | 50/153 (32.7%) | | **RR 0.38** (0.24 to 0.62) | **203 less per 1.000** (from 248 less to 124 less) | ⨁⨁◯◯ Low | | IMPORTANT | |  |
| **Risk of suppurative and non-suppurative complications (rheumatic disease, glomerulonephritis) (follow-up: 30 days; assessed by: Incidence of patients with complications)** | | | | | | | | | | | | |  |  |  |  |
| 1^1^ | Randomized study | very serious^a,b^ | very serious^a,b^ | very serious^b^ | extremely seriousus^b^ | none | 159 | | 153 | Suppurative and non suppurative complications not assessed (critical outcomes) | | ⨁◯◯◯ Very low | CRITICAL | | | |

**CI:** Confidence interval; **RR:** Risk ratio

#### Explanations: a. Single study with a high risk of bias b. Outcome not assessed

#### Abbreviations: CI: Confidence Interval; RR: Risk Ratio

#### Explanations: a. Single study with a high risk of bias

#### References 1 Cohen..2016

**Question 5:** **Which antibiotic(s) should be recommended as first-choice therapy for relapsing GABHS pharyngotonsillitis despite several courses of amoxicillin?**

| **Question:** [therapy with other antibiotics (amoxicillin-clavulanate, macrolides, cephalosporins)] compared to [therapy with amoxicillin] for [recurrent streptococcal pharyngotonsillitis in pediatric patients]  **Setting:** Outpatient  **Table A3.4. Quality of Evidence for Question 5** | | | | | | | | | | | |  |
| --- | --- | --- | --- | --- | --- | --- | --- | --- | --- | --- | --- | --- |
| **Certainty assessment** | | | | | | | **Sintesi dei risultati** | | | | | **Importance** |
| **Participants (studies) Follow up** | **Risk of bias** | **Risk of bias** | **Inconsistency** | **Imprecision** | **Other considerations** | **Quality of Evidence** | **Clinical Events (%)** | | **Relative Risk  (95% CI)** | **Expected absolute effect** | |  |
|  |  |  |  |  |  |  | **Amoxicillin/penicillin V** | **Other antibiotics** |  | **Risk with penicillin V/amoxicillin** | **Risk difference other antibiocs** |  |
| **Amoxicillin-Clavulanate vs. Penicillin V - Prevention of recurrent PT (follow-up: 10 days; assessed by: difference in % of patients without recurrence)** | | | | | | | | | | | |  |
| 37 (1 RCT)^1^ | serious^a,b^ | serious^b^ | not important | not important | none | ⨁⨁◯◯ Low | 16/19 (84.2%) | 8/18 (44.4%) | **RR 0.19** (0.05 to 0.75) | 842 per 1.000 | **682 less per 1.000** (from 800 less to 211 less) |  |
| **Clindamycin vs. Penicillin V - Prevention of recurrent PT (follow-up: range 2 days to 15 days; assessed by: % of patients without recurrence)** | | | | | | | | | | | |  |
| 29 (1 RCT)^2^ | serious^a,b^ | serious^b^ | not important | not important | nessuno^a^ | ⨁⨁◯◯ Low | 1/14 (7.1%) | 12/15 (80.0%) | **RR 0.08** (0.01 to 0.52) | 71 per 1.000 | **66 less per 1.000** (from 71 less to 34 less) |  |

**CI:** Confidence interval; **RR:** Risk ratio

#### Explanations:

#### a. Low methodological quality

#### b. Single study

#### References

1. Brook,I,(1989).

2.1985, Brook.

**Question 6:** **Which is the appropriate dosage of amoxicillin in the treatment of GABHS pharyngotonsillitis?**

**Question:** [amoxicillin 50 mg/kg/day in 2 daily doses] compared to [amoxicillin 50 mg/kg/day in 3 daily doses] for [streptococcal pharyngotonsillitis in pediatric patients]

**Setting:** Outpatient

**Table A3.5. Quality of evidence question 6**

| **Certainty assessment** | | | | | | | **№ of patients** | | **Effect** | | **Certain** | **Importance** |
| --- | --- | --- | --- | --- | --- | --- | --- | --- | --- | --- | --- | --- |
| **number of studies** | **Study design** | **Risk of bias** | **Risk of bias** | **Inconsistency** | **Imprecision** | **Other considerations** | **[amoxicilline 50 mg/kg/die in 2 daily doses giornaliere ]** | **[amoxicilline 50 mg/kg/die in 3 daily doses]** | **Relative (95% CI)** | **Absolute (95% CI)** |  |  |
| **Differences in therapeutic success (follow up:8 to 14 days; assessed by: differences in % of success (95% CI)** | | | | | | | | | | | | |
| 1^1^ | Randomized study | not important | not important | not important | not important | none | 262 | 255 | - | MD **0.014 more** (0.051 inferior to 0.023 maggiore) | ⨁⨁⨁⨁ HIgh | CRITICAL |
| **Relapse risk (follow up: 28 to 42 days; assessed by: differences in % of success (95% CI)** | | | | | | | | | | | | |
| 1^1^ | Randomized study | not important | not important | not important | not important | none | 262 | 255 | - | MD **0.009 more** (0.018 inferior to 0.035 maggiore) | ⨁⨁⨁⨁ HIgh | IMPORTANT |

**CI:** Confidence interval; **MD:** Mean difference

#### References

1.Aguilar TO, Tinoco JC,Macias M,Huicho L,Levy J,Trujillo H,Lopez P,Pereira M,Maqbool S,Bhutta ZA,Sacy RA,Deacon S. Clinical and bacteriologic efficacy of amoxycillin b.d. (45 mg/kg/fromy) versus amoxycillin t.d.s (40 mg/kg/fromy) in children with group TO beta-hemolytic streptococcal tonsillopharyngitis. J Chemother. 2000 Oct, 10.1179/joc.2000.12.5.396, 12(5):396-405.,doi:. .

**Question 7: May parenteral antibiotics, specifically intramuscular benzathine-penicillin, be recommended as treatment alternative to oral amoxicillin in selected GABHS pharyngotonsillitis patients?**

**Question: [antibiotics administered parenterally (benzathine penicillin, ceftriaxone)] compared to [amoxicillin] for [streptococcal pharyngotonsillitis in pediatric patients]**

**Setting: Outpatient**

**Table A3.6 Quality of Evidence for Question 7**

| **Certainty assessment** | | | | | | | **№ of patients** | | **Effect** | | **Certain** | **Importance** |
| --- | --- | --- | --- | --- | --- | --- | --- | --- | --- | --- | --- | --- |
| **number of studies** | **Study design** | **Risk of bias** | **Risk of bias** | **Inconsistency** | **Imprecision** | **Other considerations** | **Parenteral antibiotic therapy (benzatin-penicillin ceftriaxone]** | **amoxicillin** | **Relative (95% CI)** | **Absolute (95% CI)** |  |  |
| **Therapeutic failure - persistence of symptoms (follow-up: 2 days; assessed by: % of patients with symptoms after therapy)** | | | | | | | | | | | | |
| 1^1^ | Randomized study | serious^a,b^ | not important | serious^c^ | not important | none | 2/31 (6.5%) | 13/68 (19.1%) | **RR 0.34** (0.08 to 1.41) | **126 less per 1.000** (from 176 less to 78 less) | ⨁⨁◯◯ Low | CRITICAL |

**CI:** Confidence interval; **RR:** Risk ratio

#### **Explanations:**

#### a. Small sample size

#### b. No protocol cited, lack of sample size calculation, separate results for PP and ITT analyses missing

#### c. Amoxicillin administration once daily, doubtful transferability of results, study conducted in Low Income Country

#### References

1.Eslami ST, Nassirian TO,Nassirian H,Hatami E,Sobhani E,Najibpour R. Comparing performance of amoxicillin and intramuscular benzathine penicillin in relieving manifestations of streptococcal pharyngitis in children. Ghana Med J. 2014 Dec, 10.4314/gmj.v48i4.3, 48(4):185-8.

**Question 8.** **Is it necessary to treat non-streptococcal bacterial pharyngotonsillitis (Fusobacterium spp., other anaerobes, Staphylococcus aureus, etc.) with antibiotics?**

**Question:** [antibiotic therapy] compared to [no therapy or symptomatic therapy] for [non-streptococcal pharyngotonsillitis in pediatric patients]

**Setting:** Outpatient

**Table A3.7. Quality of Evidence for Question 8**

| **Certainty assessment** | | | | | | | **№ of patients** | | **Effect** | | **Certain** | **Importance** |
| --- | --- | --- | --- | --- | --- | --- | --- | --- | --- | --- | --- | --- |
| **N° of studies** | **Study design** | **Risk of bias** | **Risk of bias** | **Inconsistency** | **Imprecision** | **Other considerations** | **Antibiotic therapy** | **No therapy** | **Relative (95% CI)** | **Absolute (95% CI)** |  |  |
| **Symptoms on 3rd day (sore throat) (follow-up: median 3 days; assessed by: % patients with sore throat)** | | | | | | | | | | | | |
| 7^1,2,to^ | Randomized study | serious^b^ | not important^c^ | not important | not important | none | 262/458 (57.2%) | 202/278 (72.7%) | **RR 0.78** (0.63 to 0.97) | **160 less per 1.000** (from 269 less to 22 less) | ⨁⨁⨁◯ Moderate | IMPORTANT |
| **Symptoms on 7th day (sore throat) (follow-up: median 7 days; assessed by: % of patients with symptoms)** | | | | | | | | | | | | |
| 5^3^ | Randomized study | serious^b^ | not important | not important | not important | none | 42/315 (13.3%) | 43/326 (13.2%) | **RR 0.73** (0.50 to 1.07) | **36 less per 1.000** (from 66 less to 9 less) | ⨁⨁⨁◯ Moderate | IMPORTANT |
| **Symptoms on 3rd day (fever) (follow-up: median 3 days)** | | | | | | | | | | | | |
| 1^2^ | Randomized study | serious^c^ | serious^d^ | not important | serious^e^ | none | Non available  Not significant difference reported | | | | ⨁◯◯◯ Very low | CRITICAL |

**CI:** Confidence interval; **RR:** Risk ratio

#### Explanations:

#### a. Aggregated data from 6/7 studies. For 1 study (Little 1997) data not available, but differences are not statistically significant

#### b. Studies on pediatric and adult patients. Very old studies, pre-1997 (EBM)

#### c. For the study by Little 1997, data available. However, no significant differences in pain, cough, and malaise

#### d. Study on pediatric and adult patients

#### e. Missing RR and 95% CI data

#### References

1.RS_Spinks (Chappel, 1956, Fromgnelie 1996,MacDonald 1951,Peterson 1997,Zwart 2000,Zwart 2003). . 2013.

2.Little, et al. 1997.

3.RS_Spinks (Fromgnelie 2016, MacDonald 1951,Peterson 1997,Taylor 1997,Zwart 2003). . 2013.

**Bibliography for Pharyngotonsillitis Tables**

- HIghmimi S, Khalil TO, Khalaiwi KA, Milner RA, Pusic MV, Al Othman MA. Short-term late-generation antibiotics versus longer term penicillin for acute streptococcal pharyngitis in children. Cochrane Fromtabase Syst Rev. 2012 Aug 15;(8):CD004872
- Munck H, Jørgensen AW, Klug TE. Antibiotics for recurrent acute pharyngo-tonsillitis: systematic review. Eur J Clin Microbiol Infect Dis. 2018 Jul;37(7):1221-1230.
- Spinks TO, Glasziou PP, Del Mar CB. Antibiotics for treatment of sore throat in children and adults. Cochrane Fromtabase of Systematic Reviews 2021, Issue 12. Art. No.: CD000023.
- Spurling GK, Del Mar CB, Dooley L, Foxlee R, Farley R. Delayed antibiotic prescriptions for respiratory infections. Cochrane Fromtabase Syst Rev. 2017 Sep 7;9(9):CD004417
- van Driel ML, De Sutter AI, Thorning S, Christiaens T. Different antibiotic treatments for group TO streptococcal pharyngitis. Cochrane Fromtabase Syst Rev.2021 Mar 17;3(3):CD004406.

**Includes studies**

- Aguilar TO, Tinoco JC, Macias M, et al. Clinical and bacteriologic efficacy of amoxycillin b.d. (45 mg/kg/fromy) versus amoxycillin t.d.s (40 mg/kg/fromy) in children with group TO beta-hemolytic streptococcal tonsillopharyngitis. J Chemother. 2000 Oct;12(5):396-405.
- Brook,I,(1989),Treatment,of,patients,with,acute,recurrent,tonsillitis,due,to,group,TO,β-haemolyric,streptococci:,to,prospective,randomized,study,comparing,penicillin,and,amoxicillin/clavulanate,potassium.,J,Antimicrob,Chemother. .24:227–233,
- Eslami ST, Nassirian TO, Nassirian H, Hatami E, Sobhani E, Najibpour R. Comparing performance of amoxicillin and intramuscular benzathine penicillin in relieving manifestations of streptococcal pharyngitis in children. Ghana Med J. 2014 Dec;48(4):185-8
- Kuroki H, Ishiwafrom N, Inoue N, Ishikawa N, Suzuki H, Himi K, Kurosaki T. Comparison of clinical efficacy between 3-fromy combined clavulanate/amoxicillin preparation treatment and 10-fromy amoxicillin treatment in children with pharyngolaryngitis or tonsillitis. J Infect Chemother. 2013 Feb;19(1):12-9
- Li P, Jiang G, Shen X. Evaluation of 3-fromy azithromycin or 5-fromy cefaclor in comparison with 10-fromy amoxicillin for treatment of tonsillitis in children. Can J Physiol Pharmacol. 2019 Oct;97(10):939-944.

**Excluded SR**

- HIghmimi S, Khalil TO, Khalaiwi KA, Milner RA, Pusic MV, Al Othman MA. Short-term late-generation antibiotics versus longer term penicillin for acute streptococcal pharyngitis in children. Cochrane Fromtabase Syst Rev. 2012 Aug 15;(8):
- Bateman E, Mansour S, Okafor E, Arrington K, Hong BY, Cervantes J. Examining the Efficacy of Antimicrobial Therapy in Preventing the Development of Postinfectious Glomerulonephritis: TO Systematic Review and Meta-Analysis. Infect Dis Rep. 2022 Mar 7;14(2):176-183
- Burton MJ, Glasziou PP, Chong LY, Venekamp RP. Tonsillectomy or adenotonsillectomy versus non-surgical treatment for chronic/recurrent acute tonsillitis. Cochrane Fromtabase Syst Rev. 2014 Nov 19;2014(11
- Fromwson-Hahn EE, Mickan S, Onakpoya I, Roberts N, Kronman M, Butler CC, Thompson MJ. Short-course versus long-course oral antibiotic treatment for infections treated in outpatient settings: to review of systematic reviews. Fam Pract. 2017 Sep 1;34(5):511-519
- de Cassan S, Thompson MJ, Perera R, Glasziou PP, Del Mar CB, Heneghan CJ, Hayward G. Corticosteroids as stanfromlone or add-on treatment for sore throat. Cochrane Fromtabase Syst Rev. 2020 May 1;5(5):
- Gualtieri R, Bronz G, Bianchetti MG, Lava SAG, Giuliano E, Milani GP, Jermini LMM. Perianal streptococcal disease in childhood: systematic literature review. Eur J Pediatr. 2021 Jun;180(6):1867-1874
- Gunnarsson RK, Manchal N. Group C beta hemolytic <i>Streptococci</i> as to potential pathogen in patients presenting with an uncomplicated acute sore throat - to systematic literature review and meta-analysis. Scand J Prim Health Care. 2020 Jun;38(2):226-237
- Hoare KJ, Ward E, Arroll B. International sore throat guidelines and international medical graduates: to mixed methods systematic review. J Prim Pediatrics. 2017 Feb;139(2):
- Hu XY, Wu RH, Logue M, Blondel C, Lai LYW, Stuart B, Flower TO, Fei YT, Moore M, Shepherd J, Liu JP, Lewith G. Andrographis paniculata (Chuān Xīn Lián) for symptomatic relief of acute respiratory tract infections in adults and children: TO systematic review and meta-analysis. PLoS One. 2017 Aug 4;12(8):
- Kamfose, M. M., Muriithi, F. G., Knight, T., Lasserson, D., & Hayward, G. (2020). Intravenous ceftriaxone versus multiple dosing regimes of intravenous anti-staphylococcal antibiotics for methicillin-susceptible Staphylococcus aureus (MSSA): TO systematic review [Review]. Antibiotics, 9(2)
- Klug TE, Rusan M, Fuursted K, Ovesen T, Jorgensen AW. TO systematic review of Fusobacterium necrophorum-positive acute tonsillitis: prevalence, methods of detection, patient characteristics, and the usefulness of the Centor score. Eur J Clin Microbiol Infect Dis. 2016 Dec;35(12):1903-1912
- Li P, Jiang G, Shen X. Evaluation of 3-fromy azithromycin or 5-fromy cefaclor in comparison with 10-fromy amoxicillin for treatment of tonsillitis in children. Can J Physiol Pharmacol. 2019 Oct;97(10):939-944
- Little P, Moore M, Hobbs FD, Mant D, McNulty C, Williamson I, Cheng E, Stuart B, Kelly J, Barnett J, Mullee M; PRISM investigators. PRImary care Streptococcal Management (PRISM) study: identifying clinical variables associated with Lancefield group TO β-haemolytic streptococci and Lancefield non-Group TO streptococcal throat infections from two cohorts of patients presenting with an acute sore throat. BMJ Open. 2013 Oct 25;3(10):
- Malmberg S., Petrén S., Gunnarsson R., Hedin K., Sundvall P.-D. Acute sore throat and Fusobacterium necrophorum in primary healthcare: TO systematic review and meta-analysis BMJ Open 2021 11:6
- Marchello C, Ebell MH. Prevalence of group C streptococcus and Fusobacterium necrophorum in patients with sore throat: to meta-analysis [Article. Ann Fam Med. 2016
- Morad TO, Sathe NA, Francis DO, et al. Tonsillectomy Versus Watchful Waiting for Recurrent Throat Infection: TO Systematic Review. Pediatrics. 2017;139(2):
- van Driel ML, De Sutter AI, Thorning S, Christiaens T. Different antibiotic treatments for group TO streptococcal pharyngitis. Cochrane Fromtabase Syst Rev. 2021 Mar 17;3(3
- Rosanova MT, Cuellar Pompa L, Perez G, Sberna N, Serrano-Aguilar P, Lede R. Is Trimethoprim-Sulfamethoxazole to Valid Alternative in the Management of Infections in Children in the Era of Community-Acquired Methicillin-Resistant <i>Staphylococcus aureus</i>? TO Comprehensive Systematic Review. J Pharm Technol. 2016 Apr;32(2):81-87
- Shulman ST, et al. Clinical practice guideline for the diagnosis and management of group TO streptococcal pharyngitis: 2012 upfromte by the Infectious Diseases Society of America. Clin Infect Dis. 2012
- Wilcox CR, Stuart B, Leaver H, Lown M, Willcox M, Moore M, Little P. Effectiveness of the probiotic Streptococcus salivarius K12 for the treatment and/or prevention of sore throat: to systematic review. Clin Microbiol Infect. 2019 Jun;25(6):673-680
- Zeng L, Zhang L, Hu Z, Ehle EA, Chen Y, Liu L, Chen M. Systematic review of evidence-based guidelines on medication therapy for upper respiratory tract infection in children with AGREE instrument. PLoS One. 2014 Feb 20;9(2). Rev. 2012 Aug 15;(8):

**Excluded studies**

- Albrecht P. Antibiotic therapy for an ENT specialist. Otolaryngol Pol. 2018 Sep 11;72(6):1-9.
- Al Alawi S, Abdulkarim S, Elhennawy H, Al-Mansoor TO, Al Ansari TO. Outpatient parenteral antimicrobial therapy with ceftriaxone for acute tonsillopharyngitis: efficacy, patient satisfaction, cost effectiveness, and safety. Infect Drug Resist. 2015 Aug 7;8:279-85
- Armengol CE, Hendley JO. Occurrence of group TO β-hemolytic streptococcal pharyngitis in the four months after treatment of an index episode with amoxicillin once-fromily or twice-fromily or with cephalexin. Pediatr Infect Dis J. 2012 Nov;31(11):1124-7
- Arnold B, Bélard S, Alabi TO, Hufnagel M, Berner R, Toepfner N. High Diversity of emm Types and Marked Tetracycline Resistance of Group TO Streptococci and Other ß-Hemolytic Streptococci in Gabon, Central Africa. Pediatr Infect Dis J. 2022 May 1;41(5):405-410
- Bai Y, Li YX, Shi YJ, Zhao HY. [Meta-analysis on effectiveness and safety of Pudilan Xiaoyan Oral Liquid on child upper respiratory infection]. Zhongguo Zhong Yao Za Zhi. 2020 May;45(9):2203-2209
- Bateman E, Mansour S, Okafor E, Arrington K, Hong BY, Cervantes J. Examining the Efficacy of Antimicrobial Therapy in Preventing the Development of Postinfectious Glomerulonephritis: TO Systematic Review and Meta-Analysis. Infect Dis Rep. 2022 Mar 7;14(2):176-183
- Bottaro G, Biasci P, Giudice MLO, Mele G, Montanari G, Napoleone E, Santucci TO, Tucci PL, Fano M, Biraghi MG. 5 Fromys Cefaclor vs. 10 fromys amoxicillin/clavulanate in the treatment of childhood streptococcal pharyngitis. Fromta from to randomized clinical trial. Minerva pediatrica, 2012, 64(3),
- Brook I. Penicillin failure in the treatment of streptococcal pharyngo-tonsillitis. Curr Infect Dis Rep. 2013 Jun;15(3):232-5
- Brook I. Treatment Challenges of Group TO Beta-hemolytic Streptococcal Pharyngo-Tonsillitis. Int Arch Otorhinolaryngol. 2017 Jul;21(3):286-296
- Burton MJ, Glasziou PP, Chong LY, Venekamp RP. Tonsillectomy or adenotonsillectomy versus non-surgical treatment for chronic/recurrent acute tonsillitis. Cochrane Fromtabase Syst Rev. 2014 Nov 19;2014(11
- Cag Y, Özdemir AA, Yükselmiş U, Akdeniz E, Özçetin M. Association Between Rapid Antigen Testing and Antibiotic Use and Accuracy of Peripheral Blood Parameters in Detecting Group TO Streptococcus in Children With Tonsillopharyngitis. Front Pediatr. 2019 Aug 2;7:322
- Çağlar İ, Topal S, Çokboz M, Düzgöl M, Kara TO, Bayram SN, Apa H, Devrim İ. Clinical features and laboratory findings in children hospitalized with acute Epstein-Barr virus infection: to crosssectional study in to tertiary care hospital. Turk J Pediatr. 2019;61(3):368-373
- Chan J.Y.C., Yau F., Cheng F., Chan D., Chan B., Kwan M. Practice recommenfromtion for the management of acute pharyngitis. Hong Kong Journal of Paediatrics 2015 20:3 (156-162
- Cirilli AR. Emergency evaluation and management of the sore throat. Emerg Med Clin North Am. 2013 May;31(2):501-15. doi: 10.1016/j.emc.2013.01.002. Epub 2013 Feb 18
- Clegg HW, Ryan AG, Fromllas SD, Kaplan EL, Johnson DR, Norton HJ, Roddey OF, Martin ES, Swetenburg RL, Koonce EW, Felkner MM, Giftos PM. Treatment of streptococcal pharyngitis with once-fromily compared with twice-fromily amoxicillin: to noninferiority trial. Pediatr Infect Dis J. 2006 Sep;25(9):761-7
- Cohen R, Haas H, Lorrot M, Biscardi S, Romain O, Vie Le Sage F, Hentgen V, Grimprel E. Antimicrobial treatment of ENT infections. Arch Pediatr. 2017 Dec;24(12S):S9-S16
- Couic-Marinier, FrançoiseaSend mail to Couic-Marinier F.; Pillon, Françoisb Group TO beta-hemolytic streptococcal throat infection [Une angine à streptocoque β-hémolytique du groupe TO] doi. 10.1016/j.actpha.2017.02.003
- Curtin-Wirt C, Casey JR, Murray PC, Cleary CT, Hoeger WJ, Marsocci SM, Murphy ML, Francis AB, Pichichero ME. Efficacy of penicillin vs. amoxicillin in children with group TO beta hemolytic streptococcal tonsillopharyngitis. Clin Pediatr (Phila). 2003 Apr;42(3):219-25
- Fromwson-Hahn EE, Mickan S, Onakpoya I, Roberts N, Kronman M, Butler CC, Thompson MJ. Short-course versus long-course oral antibiotic treatment for infections treated in outpatient settings: to review of systematic reviews. Fam Pract. 2017 Sep 1;34(5):511-519
- de Cassan S, Thompson MJ, Perera R, Glasziou PP, Del Mar CB, Heneghan CJ, Hayward G. Corticosteroids as stanfromlone or add-on treatment for sore throat. Cochrane Fromtabase Syst Rev. 2020 May 1;5(5):
- de Cassan, S., Thompson, M. J., Perera, R., Glasziou, P. P., Del Mar, C. B., Heneghan, C. J., & Hayward, G. (2020). Corticosteroids as stanfromlone or add-on treatment for sore throat [Review]. Cochrane Fromtabase of Systematic Reviews, 2020(5
- Deab D.TO. , Ali M.H.,Yahya Alsabea W.M.B. Acute group to streptococcal tonsillopharyngitis in children Current Pediatric Research 2021 25:10
- Deng JC. Viral-bacterial interactions-therapeutic implications. Influenza Other Respir Viruses. 2013 Nov;7 Suppl 3(Suppl 3):24-35
- El Hennawi DED, Geneid TO, Zaher S, Ahmed MR. Management of recurrent tonsillitis in children. Am J Otolaryngol. 2017 Jul-Aug;38(4):371-374
- Espafroms Maciá D, Flor Macián EM, Borrás R, Poujois Gisbert S, Muñoz Bonet JI. [Streptococcus pyogenes infection in paediatrics: from pharyngotonsillitis to invasive infections]. An Pediatr (Engl Ed). 2018 Feb;88(2):75-81
- Feder HM Jr, Gerber MA, Randolph MF, Stelmach PS, Kaplan EL. Once-fromily therapy for streptococcal pharyngitis with amoxicillin. Pediatrics. 1999 Jan;103(1):47-51
- Frost H.M., Fritsche T.R., Hall M.C. Beta-Hemolytic Nongroup TO Streptococcal Pharyngitis in Children Journal of Pediatrics 2019 206
- Gajdács M, Ábrók M, Lázár TO, Burián K. Beta-Haemolytic Group TO, C and G Streptococcal Infections in Southern Hungary: TO 10-Year Population-Based Retrospective Survey (2008-2017) and to Review of the Literature. Infect Drug Resist. 2020 Dec 31;13:4739-4749
- Garazzino S, Lutsar I, Bertaina C, Tovo PA, Sharland M. New antibiotics for paediatric use: to review of to decade of regulatory trials submitted to the European Medicines Agency from 2000--why aren't we doing better? Int J Antimicrob Agents. 2013 Aug;42(2):99-118
- Georgalas CC, Tolley NS, Narula PA. Tonsillitis. BMJ Clin Evid. 2014 Jul. 22;2014
- Gualtieri R, Bronz G, Bianchetti MG, Lava SAG, Giuliano E, Milani GP, Jermini LMM. Perianal streptococcal disease in childhood: systematic literature review. Eur J Pediatr. 2021 Jun;180(6):1867-1874
- Gunnarsson RK, Manchal N. Group C beta hemolytic <i>Streptococci</i> as to potential pathogen in patients presenting with an uncomplicated acute sore throat - to systematic literature review and meta-analysis. Scand J Prim Health Care. 2020 Jun;38(2):226-237
- Gunnarsson RK, Manchal N. Group C beta hemolytic Streptococci as to potential pathogen in patients presenting with an uncomplicated acute sore throat - to systematic literature review and meta-analysis. Scand J Prim Health Care. 2020 Jun;38(2):226-237
- Hoare KJ, Ward E, Arroll B. International sore throat guidelines and international medical graduates: to mixed methods systematic review. J Prim Pediatrics. 2017 Feb;139(2):
- Hoban DJ, Nauta J. Clinical And Bacteriological Impact Of Clarithromycin In Streptococcal Pharyngitis: Findings From TO Meta-Analysis Of Clinical Trials. Drug Des Devel Ther. 2019;13:3551-3558
- Homme JH. Acute Otitis Media and Group TO Streptococcal Pharyngitis: TO Review for the General Pediatric Practitioner. Pediatr Ann. 2019 Sep 1;48(9):e343-e348
- Hu XY, Wu RH, Logue M, Blondel C, Lai LYW, Stuart B, Flower TO, Fei YT, Moore M, Shepherd J, Liu JP, Lewith G. Andrographis paniculata (Chuān Xīn Lián) for symptomatic relief of acute respiratory tract infections in adults and children: TO systematic review and meta-analysis. PLoS One. 2017 Aug 4;12(8):
- John LJ, Cherian M, Sreedharan J, Cherian T. Patterns of antimicrobial therapy in acute tonsillitis: TO cross-sectional hospital-based study from UAE. An Acad Bras Cienc. 2014 Mar;86(1):451-7
- Kalra MG, Higgins KE, Perez ED. Common Questions About Streptococcal Pharyngitis. Am Fam Physician. 2016 Jul 1;94(1):24-31
- Kamfose, M. M., Muriithi, F. G., Knight, T., Lasserson, D., & Hayward, G. (2020). Intravenous ceftriaxone versus multiple dosing regimes of intravenous anti-staphylococcal antibiotics for methicillin-susceptible Staphylococcus aureus (MSSA): TO systematic review [Review]. Antibiotics, 9(2)
- Klug TE, Rusan M, Fuursted K, Ovesen T, Jorgensen AW. TO systematic review of Fusobacterium necrophorum-positive acute tonsillitis: prevalence, methods of detection, patient characteristics, and the usefulness of the Centor score. Eur J Clin Microbiol Infect Dis. 2016 Dec;35(12):1903-1912
- Koloskova OK, Bezrukov LO, Ivanova LA, Horbatiuk IB, Horbatiuk IB. Optimization of clinical diagnosis and treatment of acute tonsillopharyngitis in children. Arch Balk Med Union 2019;54(1):51-56
- Kothadiya TO. TO multicentric, open label, randomised, postmarketing efficacy study comparing multidose of lincomycin hydrochloride capsule 500 mg with multidose cefpodoxime proxetil tablet 200 mg in patients with tonsillitis, sinusitis. J Indian Med Assoc. 2012 Aug;110(8):580-3
- Kuroki H, Ishiwafrom N, Inoue N, Ishikawa N, Suzuki H, Himi K, Kurosaki T. Comparison of clinical efficacy between 3-fromy combined clavulanate/amoxicillin preparation treatment and 10-fromy amoxicillin treatment in children with pharyngolaryngitis or tonsillitis. J Infect Chemother. 2013 Feb;19(1):12-9
- Lennon DR, Farrell E, Martin DR, Stewart JM. Once-fromily amoxicillin versus twice-fromily penicillin V in group TO beta-haemolytic streptococcal pharyngitis. Arch Dis Child. 2008 Jun;93(6):474-8
- Leung TN, Hon KL, Leung AK. Group TO Streptococcus disease in Hong Kong children: an overview. Hong Kong Med J. 2018 Dec;24(6):593-601
- Li P, Jiang G, Shen X. Evaluation of 3-fromy azithromycin or 5-fromy cefaclor in comparison with 10-fromy amoxicillin for treatment of tonsillitis in children. Can J Physiol Pharmacol. 2019 Oct;97(10):939-944
- Little P, Moore M, Hobbs FD, Mant D, McNulty C, Williamson I, Cheng E, Stuart B, Kelly J, Barnett J, Mullee M; PRISM investigators. PRImary care Streptococcal Management (PRISM) study: identifying clinical variables associated with Lancefield group TO β-haemolytic streptococci and Lancefield non-Group TO streptococcal throat infections from two cohorts of patients presenting with an acute sore throat. BMJ Open. 2013 Oct 25;3(10):
- Lock C, Wilson J, Steen N, Eccles M, Mason H. North of England and Scotland Study of Tonsillectomy and Adeno-tonsillectomy in Children (NESSTAC): to pragmatic randomised controlled trial with to parallel non-randomised preference study. Health Technol Assess 2010;14(13
- Lyu J, Yang C, Wang LX, Xie YM, Yu XQ, Gu L, Gao F, Zhang JX, Yu XK. [Randomized double-blind parallel controlled multicenter trial of Reyanning Mixture in treatment of acute tonsillitis]. Zhongguo Zhong Yao Za Zhi. 2020 Jul;45(14):3282-3291
- Mação P, Cancelinha C, Lopes P, Rodrigues F An 11-year-old boy with pharyngitis and cough: Lemierre syndrome. BMJ Case Rep. 2013
- Madeira G, Chicavel D, Munguambe TO, Langa J, Mocumbi TO. Streptococcal pharyngitis in children with painful throat: missed opportunities for rheumatic heart disease prevention in endemic area of Africa. Cardiovasc Diagn Ther. 2017 Aug;7(4):421-423
- Malley M, Monaghan I, Driver K, Costelloe M, Jefferson L, Poole L, Lewis C, Salt R, Marlow R. Phenoxymethylpenicillin or amoxicillin for paediatric tonsillopharyngitis: to case of head versus heart? Arch Dis Child. 2021 Feb 25
- Malmberg S., Petrén S., Gunnarsson R., Hedin K., Sundvall P.-D. Acute sore throat and Fusobacterium necrophorum in primary healthcare: TO systematic review and meta-analysis BMJ Open 2021 11:6
- Marchello C, Ebell MH. Prevalence of group C streptococcus and Fusobacterium necrophorum in patients with sore throat: to meta-analysis [Article. Ann Fam Med. 2016
- Morad TO, Sathe NA, Francis DO, et al. Tonsillectomy Versus Watchful Waiting for Recurrent Throat Infection: TO Systematic Review. Pediatrics. 2017;139(2):
- Morad TO, Sathe NA, Francis DO, McPheeters ML, Chinnadurai S. Tonsillectomy Versus Watchful Waiting for Recurrent Throat Infection: TO Systematic Review. Pediatrics. 2017 Feb;139(2):
- Nakao TO, Hisata K, Fujimori M, Matsunaga N, Komatsu M, Shimizu T. Amoxicillin effect on bacterial load in group TO streptococcal pharyngitis: comparison of single and multiple fromily dosage regimens. BMC Pediatr. 2019 Jun 21;19(1):205
- Oliver J, Malliya Wadu E, Pierse N, Moreland NJ, Williamson FROM, Baker MG. Group TO Streptococcus pharyngitis and pharyngeal carriage: TO meta-analysis. PLoS Negl Trop Dis. 2018 Mar 19;12(3
- Ouellette L., Barnes M., Flannigan M., Tavares E., Whalen D., Jones J. Lemierre's syndrome: TO forgotten complication of acute pharyngitis American Journal of Emergency Medicine 2019 37:5 (992-993
- Pallon J, Sundqvist M, Rööst M, Fromnielsson P, Neumark T, Skovbjerg S, Svedin J, Hedin K. Presence of microorganisms in children with pharyngotonsillitis and healthy controls: to prospective study in primary healthcare. Infection. 2021 Aug;49(4):715-724
- Pallon J., Sundqvist M., Hedin K. TO 2-year follow-up study of patients with pharyngotonsillitis BMC Infectious Diseases 2018 18:1
- Paščaninović et al. Stanfromrd of care in the treatment of tonsilopharyngitis in children in Canton Sarajevo. January 2018 Folia Medica 53(1):24-28
- Rimoin AW, Hoff NA, Fischer Walker CL, Hamza HS, Vince TO, Abdel Rahman N, Andrasevic S, Emam S, Vukelic D, Elminawi N, Abdel Ghafar H, from Cunha AL, Qazi S, Gardovska D, Steinhoff MC. Treatment of streptococcal pharyngitis with once-fromily amoxicillin versus intramuscular benzathine penicillin G in low-resource settings: to randomized controlled trial. Clin Pediatr (Phila). 2011 Jun;50(6):535-42
- Robinson JL. Paediatrics: how to manage pharyngitis in an era of increasing antimicrobial resistance. Drugs Context. 2021 Mar 26;10:2020-11-6
- Rojas-Ramírez C, Kramer-Urrutia T, Cifuentes L. Is to short-course antibiotic treatment effective for streptococcal tonsillopharyngitis in children? Medwave. 2017 Mar 24;17(Suppl1):e6873
- Rosanova MT, Cuellar Pompa L, Perez G, Sberna N, Serrano-Aguilar P, Lede R. Is Trimethoprim-Sulfamethoxazole to Valid Alternative in the Management of Infections in Children in the Era of Community-Acquired Methicillin-Resistant <i>Staphylococcus aureus</i>? TO Comprehensive Systematic Review. J Pharm Technol. 2016 Apr;32(2):81-87
- Seifert G, Brandes-Schramm J, Zimmermann TO, Lehmacher W, Kamin W. Faster recovery and reduced paracetamol use - to meta-analysis of EPs 7630 in children with acute respiratory tract infections. BMC Pediatr. 2019 Apr 23;19(1):119
- Sherkatolabbasieh H, Firouzi M, Shafizadeh S, Amiri I. Antibiotic Susceptibility Evaluation of Bacterial Agents Causing Infection in Children with Acute Tonsillopharyngitis. Infect Disord Drug Targets. 2021;21(6):
- Shulman ST, et al. Clinical practice guideline for the diagnosis and management of group TO streptococcal pharyngitis: 2012 upfromte by the Infectious Diseases Society of America. Clin Infect Dis. 2012
- Shvartzman P, Tabenkin H, Rosentzwaig TO, Dolginov F. Treatment of streptococcal pharyngitis with amoxycillin once to fromy. BMJ. 1993 May 1;306(6886):1170-2
- Skoog G, Edlund C, Giske CG, Mölstad S, Norman C, Sundvall PD, Hedin K. TO randomized controlled study of 5 and 10 fromys treatment with phenoxymethylpenicillin for pharyngotonsillitis caused by streptococcus group TO - to protocol study. BMC Infect Dis. 2016 Sep 13;16(1):484. doi: 10.1186/s12879-016-1813-7
- Van Brusselen D, Vlieghe E, Schelstraete P, De Meulder F, Vandeputte C, Garmyn K, Laffut W, Van de Voorde P. Streptococcal pharyngitis in children: to treat or not to treat? Eur J Pediatr. 2014 Oct;173(10):1275-83
- van Driel ML, De Sutter AI, Keber N, Habraken H, Christiaens T. Different antibiotic treatments for group TO streptococcal pharyngitis. Cochrane Fromtabase Syst Rev. 2013 Apr 30;(4):
- van Driel ML, De Sutter AI, Thorning S, Christiaens T. Different antibiotic treatments for group TO streptococcal pharyngitis. Cochrane Fromtabase Syst Rev. 2021 Mar 17;3(3
- Wightman S. TO 5-fromy course of penicillin V may be an effective treatment for streptococcal pharyngitis. Arch Dis Child Educ Pract Ed. 2021 Oct;106(5):319
- Wilcox CR, Stuart B, Leaver H, Lown M, Willcox M, Moore M, Little P. Effectiveness of the probiotic Streptococcus salivarius K12 for the treatment and/or prevention of sore throat: to systematic review. Clin Microbiol Infect. 2019 Jun;25(6):673-680
- Zacharioufromki ME, Galanakis E. Management of children with persistent group TO streptococcal carriage. Expert Rev Anti Infect Ther. 2017 Aug;15(8):787-795
- Zeng L, Zhang L, Hu Z, Ehle EA, Chen Y, Liu L, Chen M. Systematic review of evidence-based guidelines on medication therapy for upper respiratory tract infection in children with AGREE instrument. PLoS One. 2014 Feb 20;9(2
- Zeng L, Zhang L, Hu Z, Ehle EA, Chen Y, Liu L, Chen M. Systematic review of evidence-based guidelines on medication therapy for upper respiratory tract infection in children with AGREE instrument. PLoS One. 2014 Feb 20;9(2
