# Supplementary material for: Treatment of acute pharyngitis in children: an Italian intersociety consensus (SIPPS-SIP-SITIP-FIMP-SIAIP-SIMRI-FIMMG)
Source: Ital J Pediatr. 2024 Nov 6;50:235. doi: 10.1186/s13052-024-01789-5 (PMC11539554; doi:10.1186/s13052-024-01789-5)
Supplement: Supplementary file 5 — Additional file 5: Characteristics, Results and Conclusions of the included studies. Characteristics, Results and Conclusions of the included studies for each question are summarized in tables A3.1 – A3.8. [file 13052_2024_1789_MOESM5_ESM.docx]

**Additional file 5- Characteristics, Results and Conclusions of the included studies**

| ***Question 1. Should Group A β-haemolytic streptococcus (GABHS) pharyngotonsillitis be treated with antibiotics?*** | **P** children with GABHS pharyngotonsillitis  **I** antibiotic therapy  **C** no therapy or simptomatic therapy alone  **O1** severity and duration of symptoms  **O2** relapsing pharyngotonsillitis  **O3** suppurative complications  **O4** non suppurative complications (acute rheumatic fever [ARF], acute post-streptococcal glomerulonephritis [APSGN]) |
| --- | --- |

### Table A5.1- PICO 1- Characteristics, Results, and Conclusions of the Systematic Reviews

| **Author/year** | **Population and Purpose of the Review** | **Results** | **Conclusions** |
| --- | --- | --- | --- |
| Spinks et al. 2021 | SR of RCTs or quasi-RCTs conducted on pediatric and/or adult patients.  To evaluate the impact of antibiotic treatment in patient with pharyngotonsillitis in the outpatient setting. | 11 studies included GABHS-positive patients (Brink 1951; Brumfitt 1957; Chappel 1956; De Meyere 1992; Danny 1953; El-Daher 1991; Krober 1985; MacDonald 1951; Middleton 1988; Zwart 2000; Zwart 2003). One study reported separately the results from GABHS-positive and negative patients (Dagnelie 1996), and two studies excluded GABHS-positive patients (Petersen 1997; Taylor 1977).  Antibiotics were more effective in alleviating symptoms by day 3 in GABHS-positive patients (RR=0.58; 95% CI 0.48 to 0.71;15 high-quality RCTs, 3621 patients). Similarly, by the first week, the RR was 0.29 (95% CI 0.12 to 0.70) in GABHS-positive patients and 0.73 (95% CI 0.50 to 1.07, statistically NOT significant) in negative ones. (13 high-quality RCTs, 2974 patients).  Non-suppurative complications: antibiotic therapy was associated with a reduction in the incidence of ARF by over two-thirds within a month (RR=0.27; 95% CI 0.12 to 0.60). No conclusion was drawn for ASPGN due to the lack of evidence (low-quality RCT, 2 cases/5,147 patients).  Suppurative complications: antibiotics reduced the incidence of acute otitis media within 14 days (RR 0.30; 95% CI 0.15 to 0.58), acute sinusitis within 14 days (RR 0.48; 95% CI 0.08 to 2.76, statistically not significant), and peritonsillar abscess within two months (RR=0.15; 95% CI 0.05 to 0.47) compared to those taking a placebo. | In GABHS pharyngotonsillitis, antibiotics are effective in reducing symptoms by the 3rd day (NNT=6) and 7th day.  The reduction in the risk of ARF (RR=0.27; 95% CI 0.12 to 0.60) and suppurative complications such as AOM (RR=0.30; 95% CI 0.15 to 0.58) and peritonsillar abscess (RR=0.15; 95% CI 0.05 to 0.47) was statistically significant. |

| ***Question 2. Should amoxicillin be considered the antibiotic of choice in the treatment of GABHS pharyngotonsillitis besides penicillin V?*** | **P** children with GABHS pharyngotonsillitis  **I** antibiotic therapy (amoxicillin-clavulanate, macrolides, cephalosporyns)  **C** amoxicillin  **O1** severity and duration of symptoms  **O2** relapsing pharyngotonsillitis  **O3** suppurative complications  **O4** non suppurative complications (acute rheumatic fever [ARF], acute post-streptococcal glomerulonephritis [APSGN]) |
| --- | --- |

**Table A5.2- PICO 2-Characteristics, Results, and Conclusions of the Systematic Reviews**

| **Author/year** | **Population and Purpose of the Review** | **Results** | **Conclusions** |
| --- | --- | --- | --- |
| Altamimi et al. 2012 | Population: children 1-18 years with GABHS pharyngotonsillitis  To evaluate the impact of a short term antibiotic regimen (2-6 days) compared to the standard regimen (penicillin V for 10 days) | 20 RCTs were included (13.102 GABHS positive pharyngotonsillitis)  3 studies assessed the complication rate (Adam 2009 – 4728 patients – several antibiotics, Schaad 2002 – 292 patients– AZT 10 mg/kg, Scholtz 2004 - 1975 patients- cefuroxime 20 mg/kg/die (max 500 mg) twice daily for 5 days)  Early clinical treatment failure  Compared to standard penicillin V treatment, only Azithromycin (AZT) administered at 20 mg/kg was more effective (OR=0.08 [95% CI=0.01 0.64]). No statistically significant difference was found for AZT administered at 10 mg/kg, Clarithromycin, Cefuroxime and other antibiotics  Late clinical relapse  No statistically significant difference (OR=0.95 [0.83-1.08]).  Complications  Dalla metanalisi dei 3 studi non è risultata una differenza statisticamente significativa della probabilità di MR e glomerulonefrite (OR=0.53 [0.17,1.64]) | A three to six-day course of oral antibiotics showed comparable efficacy to the standard ten-day course of oral penicillin in the treatmente of children with GABHS pharyngotonsillitis.  In regions with a high prevalence of rheumatic heart disease, these findings should be interpreted with caution. |
| Van Driel et al. 2021 | Patients with proven GABHS pharyngotonsillitis aged 1 month to 80 years.  To compare the efficacy of different antibiotics in: (a) alleviating symptoms (pain, fever); (b) shortening the duration of symptoms; (c) preventing clinical relapses (i.e., recurrence of symptoms after initial resolution); and (d) preventing complications (suppurative complications, ARF,ASPGN).  To evaluate the incidence of adverse effects among different antibiotic regimens.  To assess the risk-benefit ratio of antibiotic therapy in treating GABSH pharyngotonsillitis. | 19 studies reported in 18 papers (5839 randomized participants): 6 studies compared penicillin with cephalosporins; 6 compared penicillin and macrolides; 3 studies compared penicillin and carbacefem; 1 study compared penicillin and sulfonamides; 1 clindamicin and ampicillin; 1 AZT and amoxicillin in children.  *Cephalosporins versus penicillin*  No significant differences were reported regarding symptom resolution for cephalosporins compared to penicillin (OR for lack of symptom resolution 0.79, 95%CI = 0.55 to 1.12; 5 studies; 2018 participants; low-quality evidence). Sensitivity analysis results: OR=0.51, 95% CI = 0.27 to 0.97 (5 studies; 1660 participants; very low-quality evidence). ITT analysis results on 855 children: OR=0.83, 95% CI = 0.40 to 1.73.  The clinical recurrence was not significantly lower for cephalosporins compared to penicillin (OR= 0.55, 95% CI 0.30 to 0.99; NNT = 50; 4 studies; 1,386 participants; low-quality evidence). No difference in the adverse event rates among groups was reported (Very low-quality evidence).  *Macrolides versus penicillin*  The difference in symptom resolution between macrolides and penicillin was not statistically significant across groups (OR=1.11, 95%CI=0.92 to 1.35; 6 studies; 1,728 participants; low-quality evidence). Sensitivity analysis results: OR 0,79; 95% CI 0,57 to 1,09; (6 studies; 1159 participants)  The risk of clinical recurrence did not differ significantly (OR=1.21, 95% CI 0.48 to 3.03; 6 studies; 802 participants; low-quality evidence).  *Azithromycin versus amoxicillin*.  Symptom resolution did not show a significant difference when comparing a single dose of AZT with a 10-day course of amoxicillin (OR = 0.76, 95% CI 0.55 to 1.05; 1 study; 673 participants; very low-quality evidence).  Per protocol sensitivity analysis results: OR = 0,29, CI95% 0,11 to 0,73; 1 trial; 482 participants; very low certainty).  Adin patients receiving AZT than those treated with amoxicillin (OR 2,67, CI95% 1,78 to 3,99; 1 study; 673 participants; very low quality). | The considered antibiotic regimens showed similar efficacy, and no relevant differences in the adverse event rates. No conclusion could be drawn for long-term complications since they were rarely reported.  All studies were conducted in high-income countries with a low-incidence of GABHS related complications. Studies from low-income countries and including participants from aborigenal community with increased risk of GABHS-related complications are needed.  The review supports the use of penicillin as first choice antibiotic drug in children with GABHS pharyngotonsillitis. |

**Table A5.3- PICO 2- Characteristics, Results, and Conclusions of clinical studies**

| **Author/Year** | **Study design** | **Population (N°, Country, Setting, Condition)** | **Intervention/Exposure** | **Primary Outcome** | **Effect measures** | **Secondary Outcomes** | **Follow-up** | **Results** | **Funding** |
| --- | --- | --- | --- | --- | --- | --- | --- | --- | --- |
| Kuroki et al. 2013 | RCT | Japan; outpatient setting; 119 patients aged <15 years (2-13 years, mean age 5.6 years) with acute pharyngotonsillitis or pharyngolaryngitis, positive RADT for GABHS.  Culture form throat swab, including identification, and quantification of GABHS and other bacteria (*S.pneumoniae, H.influenzae, M.catarrhalis, Neisseria* spp., alpha-hemolytic streptococci).  Minimum inhibitory concentration (MIC) of each antimicrobial drug (8 drugs) for each isolated strain was measured. | Amoxicillin-clavulanate for 3 days vs. amoxicillin for 10 days | Evaluate and compare the clinical efficacy of azithromycin or cefaclor with amoxicillin | Statistical significance of chi-squared test | Bacteriological efficacy (bacterial eradication in culture after treatment).  Adverse events rate. | 1-2 weeks after the end of treatment | Patients included in the efficacy assessment: 54 in the clavulanate/amoxicillin group and 43 in the amoxicillin group.  Response rate at the end of treatment = 98.1% in the clavulanate/amoxicillin group and 92.9% in the amoxicillin group.  GABHS eradication rate at follow-up: 65.4% in the clavulanate/amoxicillin group and 85.4% in the amoxicillin group. Even in the case of persistent positive cultere clinical symptoms rarely recurred.  Urinalysis: no abnormalities in any patient. | The principal investigator received financial support from Glaxo-SmithKline K.K. |
| Li et al. 2019 | RCT | 256 children with proven GABHS tonsillitis confirmed by culture. Age 2-12 years.  Azithromycin (n = 85), cefaclor (n = 88), and amoxicillin (n = 83) groups | \| Azithromycin (10 mg/kg daily for 3 days) or cefaclor (20 mg/kg daily in 3 divided doses for 5 days) vs. amoxicillin (30 mg/kg daily in 3 divided doses for 10 days). \| \| --- \| | To evaluate and compare the clinical efficacy of azithromycin or cefaclor with amoxicillin |  |  | 14 and 30 days | Clinical success was reported in 96,4% of patients treated with AZT, 92,4% of those receiveing cefaclor and 91,0% in the amoxicillin group.  No statistically significant differences were reported for AZT, cefaclor and amoxicillin, when the following outcomes were considered:  -Bacteriological eradication rates at the end of therapy: 94,0%, 89,9% and 88,5%, respectively  -Recurrence rate: 2,6%, 7,0% and 5,9% respectively  Regarding adverse events, a lower overall risk was reported for AZT (2.4% of patients) compared to cefaclor (11.3% of patients; p=0.030) and amoxicillin (11.4% of patients; p=0.029). | Not reported |

| ***Question 3. Should the duration of antibiotic therapy for GABHS pharyngotonsillitis be shorter than 10 days?*** | **P** children with GABHS pharyngotonsillitis  **I** antibiotic therapy < 10 days  **C** antibiotic therapy > 10 days  **O1** severity and duration of symptoms  **O2** relapsing pharyngotonsillitis  **O3** suppurative complications  **O4** non suppurative complications (acute rheumatic fever [ARF], acute post-streptococcal glomerulonephritis [APSGN]) |
| --- | --- |

**Table A5.4-PICO 3- Characteristics, Results, and Conclusions of Systematic Reviews**

| **Author/Year** | **Population and purpose of the review** | **Outcome** | **Results** | **Conclusions** |
| --- | --- | --- | --- | --- |
| Altamimi et al. 2012 | 1 study (Cohen 1996) including 277 children compared amoxicillin (25 mg/kg/dose twice a day) for 6 days and penicillin V for 10 days  321 children aged 3-15 years (mean age 5.9 years). Of these 318 (160 receiving amoxicillin, 158 penicillin V) were evaluable for safety and 277 (86,3%) for efficacy. | Clinical and bacteriological efficacy of amoxicillin and penicillin V treatment in children with GABHS pharyngotonsillitis.  Safety of amoxicillin and penicillin V treatment in children with GABHS pharyngotonsillitis | OR= 0.82 [0.37,1.79] | No statistically significant difference was reported for efficacy and safety of amoxicillina (50 mg/kg/die administered in 2 doses) for 6 days compared to penicillin (45 mg/kg/die administered in 3 doses) for 3 days in the treatmente of GABHS pharyngotonsillitis. |

| ***Question 4. In children allergic to penicillin, which antibiotics can be administered for the treatment of GABHS pharyngotonsillitis?*** | **P** children with GABHS pharyngotonsillitis and penicillin allergy  **I** macrolides, cephalosporyns  **C** amoxicillin  **O1** severity and duration of symptoms  **O2** relapsing pharyngotonsillitis  **O3** suppurative complications  **O4** non suppurative complications (acute rheumatic fever [ARF], acute post-streptococcal glomerulonephritis [APSGN]) |
| --- | --- |

No pertinent studies retrieved

| ***Question 5: Which antibiotic(s) should be recommended as first-choice therapy for relapsing GABHS pharyngotonsillitis despite several courses of amoxicillin?*** | **P** children with relapsing GABHS pharyngotonsillitis after amoxicillin (50 mg/kg/die)  **I1** Amoxicillin 80-90 mg/kg/die  **I2** Amoxicillin-clavulanate  **I3** cephalosporin  **I4** macrolides  **I5** other (i.e. clindamycin, Rifampicin, Cotrimoxazole, etc.)  **C** Phenoxymethylpenicillin (penicillin V)  **O1** severity and duration of symptoms  **O2** relapsing pharyngotonsillitis  **O3** suppurative complications  **O4** non suppurative complications (acute rheumatic fever [ARF], acute post-streptococcal glomerulonephritis [APSGN]) |
| --- | --- |

**Table A5.5-PICO 5- Characteristics, Results, and Conclusions of Systematic Reviews**

| **Author/Year** | **Population and purpose of the SR** | **Results** | **Conclusions** |
| --- | --- | --- | --- |
| Munck et al. 2018 | **Population:** Children and adults in any clinical setting with recurrent acute pharyngotonsillitis (RAPT) (with or without active symptoms) or with recurrence of pharyngotonsillitis  **Purpose:** to evaluate the current available evidence regarding antibiotic treatment in 3 clinical scenarios: (1) patients with RAPT without ongoing infection, (2) patients with RAPT with ongoing infection and (3) patients with RAPT shortly after the end of antibiotic therapy.  Q1. Can antibiotic treatment prevent future episodes of pharyngotonsillitis in patients with RAPT? Q2. Which antibiotic therapy is preferable for treating pharyngotonsillitis in patients with RAPT? Q3. Which antibiotic therapy is preferable for treating recurrence of pharyngotonsillitis? | No RCTs met the inclusion criteria for the third question of the review (Q3). However, current literature suggests that penicillin may not be the most appropriate choice for treating recurrent pharyngotonsillitis.  In the study by Lildholdt et al. (2013) no significant difference was reported for AZT compared to placebo in reducing the number of recurrences. Two studies conducted by Brook et al. in 1989, including 19 and 18 patients respectively, showed that clindamycin and amoxicillin/clavulanic acid were superior to oral penicillin in resolving symptoms and eradicating beta-lactamase-producing bacteria from the pharynx, supporting a possible role of these bacterua in the pathogenesis of recurrences (RR = 0.15, 95% CI 0.04–0.56, p = 0.005 for clindamycin; RR = 0.19, 95% CI 0.05–0.75, p = 0.018 for amoxicillin/clavulanic acid) (Brook et al. 1989a, Brook et al. 1989b).  The included RCTs reported a high risk of bias and heterogeneity regarding the age of the study population, the number of episodes per year, and the antibiotic regimen. | Penicillin may not be the most appropriate treatment for recurrent pharyngotonsillitis; however, no pertinent studies have been conducted. |

| ***Question 6: Which is the appropriate dosage of amoxicillin in the treatment of GABHS pharyngotonsillitis?*** | **P** children with GABHS pharyngotonsillitis  **I** amoxicillin 50 mg/kg/die divided in 2 doses q12h  **C** amoxicillin 50 mg/kg/die divided in 3 doses q8h  **O1** severity and duration of symptoms  **O2** relapsing pharyngotonsillitis  **O3** suppurative complications  **O4** non suppurative complications (acute rheumatic fever [ARF], acute post-streptococcal glomerulonephritis [APSGN]) |
| --- | --- |

| **Table A5.6- PICO 6- Characteristics, Results, and Conclusions of clinical studies**   \| **Author/Year** \| **Study design** \| **Population (N°, Country, Setting, Condition)** \| **Intervention/Exposure** \| **Primary Outcome** \| **Effect measure** \| **Secondary Outcomes** \| **Follow-up** \| **Results** \| **Funding** \| \| --- \| --- \| --- \| --- \| --- \| --- \| --- \| --- \| --- \| --- \| \| Aguilar et al. 2000 \| Randomized, observer-blind, parallel-group study \| 517 children aged 2 to 12 years. Inclusion criteria; at least one of the following signs of acute bacterial pharyngotonsillitis: sore throat, pharyngeal erythema, tonsillar hypertrophy, purulent exudate, painful cervical adenitis;  at least one of the following symptoms: fever, nausea/vomiting, malaise, anorexia, abdominal pain, headache;  proven GABHS infection through enzyme immunoassay. \| amoxicillin 45 mg/kg/die administered in 2 doses (n=262) or 40 mg/kg/die ijn 3 doses (n=255), for 7 days. \| Efficacy of amoxicillin 45 mg/kg/die in 2 doses comapred to the standard regimenn (40 mg /kg/die in 3 doses) in the treatment of children with acute GABHS pharyngotonsillitis \| Clinical response at the end of treatment (day 11). \| clinical response at follow-up (Day 35±7), bacteriological response at the end of treatment (Days 8-14) and at follow-up (Day 35). \| At day 35±7 to assess clinical and bacteriological response. \| A positive clinical response was reported, at the end of treatment, in more than 96% of each group. A similar results was found at follow-up ( day 35±7).  A microbiological positive response was reported, at the end of the treatmente, in over 94% of children, who underwent a microbiological test, in both groups. Both regimen were well tolerated.  Amoxicillin administered at 45 mg/kg/die divided in 2 doses is as efficacious and as well tolerated as amoxicillin administered at 40 mg/kg/die in 3 doses in the treatment of GABHS pharyngotonsillitis. \| Grant by SmithKline Becham International. \| | | |  |
| --- | --- | --- | --- | --- | --- | --- | --- | --- | --- | --- | --- | --- | --- | --- | --- | --- | --- | --- | --- | --- | --- | --- | --- |
| ***Question 7: May parenteral antibiotics, specifically intramuscular benzathine-penicillin, be recommended as treatment alternative to oral amoxicillin in selected GABHS pharyngotonsillitis patients?*** | **P** children with GABHS pharyngotonsillitis  **I** parenteral antibiotictheraoy, specifically intramuscular administration (benzathine penicillin G, ceftriaxone)  **C** oral amoxicillin  **O1** severity and duration of symptoms  **O2** relapsing pharyngotonsillitis  **O3** suppurative complications  **O4** non suppurative complications (acute rheumatic fever [ARF], acute post-streptococcal glomerulonephritis [APSGN]) |  |  |
|  |  |  |  |
| **Table A5.7- PICO 7- Characteristics, Results, and Conclusions of clinical studies** |  |  |  |

| **Author/Year** | **Study design** | **Population (N°, Country, Setting, Condition)** | **Intervention/Exposure** | **Primary Outcome** | **Effect measure** | **Secondary Outcomes** | **Follow-up** | **Results** | **Funding** |
| --- | --- | --- | --- | --- | --- | --- | --- | --- | --- |
| Eslami et al. 2014 | RCT | 99 children aged 6 to 15 years with acute pharyngitis from 45 primary schools in 7 regions in Mashhad (Iran).  Children were excluded in case of previous antibiotic treatment,culture negative for GABHS, or drug allergy. | intramuscular benzathine penicillin G (BPG) (n=31) and a single dose of amoxicillin (n=68) for GABHS pharyngitis treatment | Clinical and microbiological efficacy | Clinical and microbiological response | None | Children were re-evaluated after 48 hours from the start of treatment.  Signs and symptoms were evaluated and compared betweenpatients with GABHS positive and negative culture.  In those with proven GABHS pharyngititis, the efficacy of the two antibiotic regimen was compared. | The treatment failure rate was higher in those treated with amoxicillin comapred to those receiveing BPG (18,9% vs. 6,4%, respectively). However the difference was not statistically signicant (P <0,05).  Both regimen were efficacious in the reduction of pharyngotonsillitis symptoms. | Not reported |

| ***Question 8. Is it necessary to treat non-streptococcal bacterial pharyngotonsillitis (Fusobacterium spp., other anaerobes, Staphylococcus aureus, etc.) with antibiotics?*** | **P** children with non-GABHS pharyngotonsillitis  **I** antibiotic therapy  **C** no therapy or simptomatic therapy alone  **O1** severity and duration of symptoms  **O2** relapsing pharyngotonsillitis  **O3** suppurative complications |
| --- | --- |

**Table A5.8- PICO 8- Characteristics, Results, and Conclusions Systematic Reviews**

| **Author/Year** | **Population and purpose of the SR** | **Results** | **Conclusions** |
| --- | --- | --- | --- |
| Spinks et al. 2013 | SR of RCT or quasi-RCTs conducted in adult and/or children.  To evaluate the impact of antibiotic treatment in patients with acute pharyngotonsillitis in an outpatient setting | 7 studies included, or separately analysed, only GABHS positive patients. (Catanzaro 1954; De Meyere 1992; El-Daher 1991; Krober 1985; Middleton 1988; Nelson 1984; Pichichero 1987), 1 study compared GABHS positive and negative patients (Dagnelie 1996) and 2 studies excluded GABHS positive patients (Petersen 1997; Taylor 1977).  Antibiotics were more effective against symptoms on day three (RR = 0.58; 95% CI 0.48 to 0.71) for GABHS pharyngotonsillitis with a positive throat culture (NNT = 6=, compared to negative ones (RR = 0.78; 95% CI 0.63 to 0.97 NNT = 21; Fifteen RCTs, high quality, 3621 patients).  Similarly, at the first week, the RR was 0.29 (95% CI 0.12 to 0.70) for GABHS pharyngotonsillitis with a positive throat culture and 0.73 (95% CI 0.50 to 1.07; statistically non-significant) for those with a negative culture. (Thirteen RCTs, high quality, 2974 patients.)  **Suppurative complications**  One study assessed the acute otitis media incidence at 14 days (Taylor 1977) RR= 0.06 [CI 95% 0,1 to 03] | In non-streptococcal bacterial pharyngotonsillitis, antibiotics are effective in reducing symptoms by day 3 (RR = 0.78; 95% CI 0.63 to 0.97 – NNT = 21), but there is no statistically significant difference by day 7 (RR = 0.73; 95% CI 0.50 to 1.07).  Only one study evaluated the incidence of acute otitis media within 14 days (Taylor 1977) with RR = 0.06 [95% CI 0.01 to 0.30]. |
| Spurling et al. 2017 | SR of RCTs conducted in pediatric and/or adult patients.  To evaluate the clinical benefits, bacterial resistance, and patient satisfaction associated with delayed or no use of antibiotics for upper respiratory tract infections (fever, sore throat, cough, etc.) in primary care settings and emergency departments. | 11 studies included studi of which 5 included only children (Chao 2008; El-Daher 1991; Little 2001; Pichichero 1987; Spiro 2006) 4 included both children and adults (Arroll 2002a; Gerber 1990; Little 1997; Little 2005a).  4 studies evaluated sore thoat in children (Pichichero 1987; Gerber 1990; El Daher 1991; Little 1997). | The results for clinical outcomes were often heterogeneous. For most outcomes, there was no evidence of a difference between delayed or immediate antibiotic administration or no antibiotic therapy. It was not possible to aggregate the study data for the comparison between delayed antibiotic administration and no antibiotic therapy due to insufficient data. |
